# Evaluating surrogates of genetic diversity for conservation planning

**DOI:** 10.1111/cobi.13602

**Published:** 2020-10-22

**Authors:** Jeffrey O. Hanson, Ana Veríssimo, Guillermo Velo‐Antón, Adam Marques, Miguel Camacho‐Sanchez, Íñigo Martínez‐Solano, Helena Gonçalves, Fernando Sequeira, Hugh P. Possingham, Silvia B. Carvalho

**Affiliations:** ^1^ CIBIO/InBIO, Centro de Investigação em Biodiversidade e Recursos Genéticos Universidade do Porto Campus de Vairão, Rua Padre Armando Quintas, no. 7 Vairão 4485‐661 Portugal; ^2^ Museo Nacional de Ciencias Naturales‐CSIC Calle de José Gutiérrez Abascal 2 Madrid 28006 Spain; ^3^ Museu de História Natural e da Ciência Universidade do Porto Praça Gomes Teixeira Porto 4099‐002 Portugal; ^4^ The Nature Conservancy Minneapolis MN 55415 U.S.A.; ^5^ Centre for Biodiversity and Conservation Science, School of Biological Sciences The University of Queensland Brisbane QLD 4072 Australia

**Keywords:** allelic richness, evolutionary processes, microsatellites, prioritization, protected‐area systems, reserve selection, systematic conservation planning, refugia, microsatélites, planeación sistemática de la conservación, priorización, procesos evolutivos, refugios, riqueza de alelos, selección de reservas, sistemas de áreas protegidas, 等位基因丰度, 演化过程, 微卫星, 优先次序, 保护区系统, 保护区选择, 系统保护规划, 避难所

## Abstract

Protected‐area systems should conserve intraspecific genetic diversity. Because genetic data require resources to obtain, several approaches have been proposed for generating plans for protected‐area systems (prioritizations) when genetic data are not available. Yet such surrogate‐based approaches remain poorly tested. We evaluated the effectiveness of potential surrogate‐based approaches based on microsatellite genetic data collected across the Iberian Peninsula for 7 amphibian and 3 reptilian species. Long‐term environmental suitability did not effectively represent sites containing high genetic diversity (allelic richness). Prioritizations based on long‐term environmental suitability had similar performance to random prioritizations. Geographic distances and resistance distances based on contemporary environmental suitability were not always effective surrogates for identification of combinations of sites that contain individuals with different genetic compositions. Our results demonstrate that population genetic data based on commonly used neutral markers can inform prioritizations, and we could not find an adequate substitute. Conservation planners need to weigh the potential benefits of genetic data against their acquisition costs.

## Abstract 2

*Article impact statement*: Conservation plans based on supposed surrogates of genetic data can perform much worse than those based directly on genetic data.

## Abstract (es)

Evaluación de los Sustitutos de la Diversidad Genética para la Planeación de la Conservación

**Resumen**

Los sistemas de áreas protegidas deberían conservar la diversidad genética intraespecífica. Ya que para obtener datos genéticos se requieren recursos, se han propuesto distintas estrategias para generar los planes para los sistemas de áreas protegidas (priorizaciones) cuando los datos genéticos no están disponibles. A pesar de lo anterior, dichas estrategias basadas en sustitutos han sido poco evaluadas. Evaluamos la efectividad del potencial de las estrategias basadas en sustitutos cuya base son los datos genéticos de microsatélites obtenidos en toda la Península Ibérica y correspondientes a siete especies de anfibios y a tres de reptiles. La idoneidad ambiental a largo plazo no representó efectivamente los sitios que contienen una diversidad genética alta (riqueza de alelos). Las priorizaciones basadas en la idoneidad ambiental a largo plazo tuvieron un desempeño similar a las priorizaciones aleatorias. Las distancias geográficas y las distancias de resistencia basadas en la idoneidad ambiental contemporánea no siempre fueron sustitutos efectivos para la identificación de las combinaciones de sitios que contienen individuos con composiciones genéticas diferentes. Nuestros resultados demuestran que los datos genéticos de una población basados en marcadores neutrales de uso común pueden informar a las priorizaciones y que no pudimos encontrar un sustituto adecuado. Los planificadores de la conservación necesitan sopesar los beneficios potenciales de los datos genéticos contra sus costos de adquisición.

## 摘要

保护区系统应当保护物种种内的遗传多样性。然而, 由于遗传数据的获取需要较多资源, 因此, 当不能获得遗传数据时, 有几种方法可用于制定保护区系统规划 (优先排序) 。目前, 这些基于替代指标的方法尚未得到深入检测。本研究基于在伊比利亚半岛收集的7种两栖动物和3种爬行动物的微卫星遗传数据, 评估了替代方法的有效性。长期环境适宜性不能有效地代表含有高遗传多样性 (等位基因丰度) 的位点。因此, 基于长期环境适宜性进行保护优先排序的效果类似于随机排序。地理距离和基于当前环境适宜性的阻隔距离并不总能有效地作为识别含有不同遗传背景个体的位点组合的替代指标。以上研究结果表明, 基于常用中性标记的群体遗传数据可以为保护优先排序提供信息, 而我们未能找到合适的替代指标。因此, 保护规划者需要权衡基因数据的潜在效益和获取成本。**【翻译: 胡怡思; 审校: 聂永刚】**

## Introduction

Protected areas are needed to maximize the long‐term persistence of biodiversity (e.g., Watson et al. 2014). Because resources are limited, plans for protected‐area systems (prioritizations) need to fulfill conservation objectives for minimum cost (Margules & Pressey 2000). Genetic diversity reflects historical demographic processes and determines the fitness, long‐term persistence, and adaptive potential of populations (Smith et al. 2014). Although genetic diversity can inform reserve selection (e.g., Diniz‐Filho et al. 2012; Nielsen et al. 2017; Paz‐Vinas et al. 2018), the resources required to obtain and analyze genetic data for multiple species across a large geographic extent are not trivial (Puckett 2017). As a consequence, there has been growing interest in identifying surrogates for genetic diversity (Carvalho et al. 2011; Ponce‐Reyes et al. 2014; Hanson et al. 2017).

Prioritizations should ideally represent intraspecific genetic diversity at multiple scales (Beger et al. 2014; Carvalho et al. 2019). At the site level, prioritizations should represent sites that each have a diverse group of genotypes (hereafter, site‐level genetic diversity [akin to alpha diversity]) (Petit et al. 1998) because such sites can harbor demographically stable populations and replenish declining populations (Smith et al. 2014). At broader scales, prioritizations should secure a representative sample of the combinations of genotypes that exist (hereafter, broad‐scale genetic diversity [akin to gamma diversity]) (Carvalho et al. 2017). This is important to prevent loss of local adaptations and erosion of biodiversity (Moritz 2002). Thus, an optimally sited prioritization would, for a given species, select a subset of sites that have sufficient site‐level genetic diversity and represent the broad‐scale genetic diversity among all sites (Beger et al. 2014). Such a prioritization would ideally reflect genome‐wide patterns of genetic diversity (i.e., adaptive and neutral components) (Moritz 2002).

Conservation planners have been advised to employ surrogate‐based approaches when genetic data are not available (Carvalho et al. 2011; Ponce‐Reyes et al. 2014; Hanson et al. 2017). These approaches aim to enhance site‐level or broad‐scale genetic diversity separately. Because site‐level genetic diversity accumulates in stable populations over long periods, conserving sites that have contained highly suitable environmental conditions for a long period (e.g., climatic refugia) could potentially secure site‐level genetic diversity (Comps et al. 2001; Carnaval et al. 2009; Abellán & Svenning 2014). In contrast, broad‐scale genetic diversity accumulates with reductions in gene flow between sites (Moritz 2002; Smith et al. 2014). Because populations often experience less gene flow if they are further apart (per isolation by distance; Wright 1943) or separated by unsuitable areas (per isolation by resistance; McRae et al. 2008), spreading conservation priorities across candidate sites based on such criteria (i.e., geographic and resistance distances, respectively) could secure broad‐scale genetic diversity (Ponce‐Reyes et al. 2014; Hanson et al. 2017). Yet these surrogate‐based approaches for conserving genetic diversity remain poorly tested in systems where complex phylogeographic histories could hinder their effectiveness.

We assessed surrogate‐based approaches for conserving genetic diversity with 7 amphibian and 3 reptilian species in the Iberian Peninsula. This region has an extraordinarily complex phylogeographic history, where climate‐induced cycles of retreat to refugia and subsequent expansion have created a mosaic of intraspecific genetic diversity (Carvalho et al. 2017). We used neutrally evolving nuclear microsatellite data to approximate genome‐wide patterns of genetic diversity for the study species. We then generated and evaluated prioritizations for site‐level genetic diversity (allelic richness) based on genetic data, long‐term environmental suitability (over the last 17,000 years), and random selection. We also generated and evaluated prioritizations for broad‐scale genetic diversity based on genetic distances, geographic distances, resistance distances (based on contemporary environmental suitability), and random selection.

## Methods

### Data

Our study area encompassed the Iberian Peninsula. We compiled nuclear microsatellite genotype and sampling site data—encompassing most of each species’ geographic range—for 7 amphibian and 3 reptilian species from previous studies (Table 1) (Sequeira et al. 2008; Gonçalves et al. 2009; Remón et al. 2013; Dias et al. 2015; Ferchaud et al. 2015; Gutiérrez‐Rodríguez et al. 2017a, 2017b; Maia‐Carvalho et al. 2018; Pereira et al. 2018; Valbuena‐Ureña et al. 2018) (Supporting Information). These species are endemic—or very nearly endemic—to the Iberian Peninsula and have different environmental affinities, with Mediterranean or Eurosiberian‐Atlantic distributions. They also have different evolutionary histories and, due to species‐specific responses to climatic oscillations during the Pleistocene, exhibit contrasting patterns of genetic diversity and genetic structure across their spatial distributions.

**Table 1:** Summary statistics of species considered in the examination of surrogates of genetic diversity for conservation planning

Species	Range size (km^2^)^a^	Conservation status^b^	*n* (% of samples)^c^	No. of microsatellite markers (%)	No. of sites (%)^c^	Median no. of samples per site (range)
*Alytes cisternasii*	118,600	NT	225 (100)	6	11 (100)	19 (15–30)
*Alytes dickhilleni*	18,600	VU	278 (57)	20	20 (26)	13 (10–26)
*Alytes obstetricans*	243,700	LC	652 (85)	12	37 (66)	18 (10–30)
*Calotriton asper*	21,500	NT	756 (84)	19	31 (70)	20 (10–69)
*Chioglossa lusitanica*	42,800	VU	288 (100)	7	13 (100)	22 (16–27)
*Emys orbicularis occidentalis*	74,600	NT	368 (81)	7	14 (67)	27 (12–36)
*Iberolacerta bonnali*	3,300	NT	372 (100)	12	13 (100)	30 (14–31)
*Iberolacerta monticola*	16,500	VU	316 (100)	11	14 (100)	18 (11–43)
*Pelobates cultripes*	238,100	NT	220 (49)	14	19 (39)	10 (10–19)
*Pleurodeles waltl*	195,100	NT	247 (60)	10	23 (55)	10 (10–19)

^a^ Based on national atlases.

^b^ Conservation status is for *Emys orbicularis* (Tortoise & Freshwater Turtle Specialist Group 1996).

^c^ Percentages are reported relative to original data set.

After cleaning the genetic data (Supporting Information), we calculated genetic diversity metrics for each species (Supporting Information). Specifically, we calculated rarefied allelic richness for each site for each species to estimate site‐level genetic diversity. We also calculated pairwise genetic distances (Jost's *D*) between sites for each species to estimate broad‐scale genetic diversity (Jost 2008). All analyses were performed with the R statistical environment (version 3.5.3) (R Core Team 2019). Genetic data were processed with the adegenet, hierfstat, mmod, and related R packages (Jombart 2008; Winter 2012; Goudet & Jombart 2015; Pew et al. 2015).

### Surrogate Variables

We collated data for fitting and projecting environmental niche models (Supporting Information). First, we created a grid with 10 × 10 km cells over the study area to standardize spatial analyses (UTM Zone 30N). Second, we obtained species distribution data from national atlases (10 × 10 km resolution) (Pleguezuelos et al. 2002; Loureiro et al. 2008). To reduce commission errors, these data were refined by excluding locations outside of the species’ known geographic ranges (buffered by 30 km), except for *Emys orbicularis occidentalis* for which range data were not yet available (NatureServe & IUCN 2019). Third, we obtained data for 8 bioclimatic variables to characterize contemporary (1979–2013) climate regimes (BIO1, annual mean temperature; BIO3, isothermality; BIO4, temperature seasonality; BIO8, mean temperature of wettest quarter; BIO9, mean temperature of driest quarter; BIO13, precipitation of wettest month; BIO15, precipitation seasonality; and BIO18, precipitation of warmest quarter; $2.5^{\prime }$ resolution [Karger et al. 2017]). These variables were selected from the 19 available bioclimatic variables by minimizing multicollinearity (with the usdm R package) (Naimi et al. 2014). We also obtained soil bedrock data to characterize the potential distribution of vegetation communities across space and time because bedrock conditions are expected to remain relatively constant over time (1 km^2^ resolution) (missing data were interpolated based on the modal values of adjacent areas) (Panagos 2006; van Liedekerke et al. 2006).

Fourth, we obtained projections of the bioclimatic variables for 6 historical periods (300–4,200, 4,200–8,326, 8,326–11,700, 11,700–12,900, 12,900–14,700, and 14,700–17,000 years ago) (Fordham et al. 2017; Karger et al. 2017; Brown et al. 2018). These periods were chosen because microsatellite markers have relatively fast evolutionary rates and are more likely to reflect historical rather than ancient periods (e.g., Last Interglacial Period, approximately 120,000–140,000 years ago) (Jarne & Lagoda 1996). Although climatic data were available for the Last Glacial Maximum (approximately 21,000 years ago), we excluded this period because it had such different climatic conditions that environmental niche model predictions would have been sensitive to extrapolation issues (Supporting Information). The data sets were then reprojected to the spatial grid. Spatial analyses were completed with the sf and raster R packages (Pebesma 2018; Hijmans 2019).

We generated environmental niche models for each species (via the biomod2 R package) (Thuiller et al. 2020) based on the atlas and contemporary environmental data (10 × 10 km resolution) (Supporting Information). Because historical climate data are not available at finer spatial resolutions without sacrificing temporal resolution, the models were used to produce continuous maps of contemporary and historic environmental suitability at 10 × 10 km resolution to predict environmental suitability over time. They were also used to produce continuous maps of contemporary environmental suitability at 1 × 1 km resolution.

We generated potential surrogate variables for genetic diversity (Supporting Information). For site‐level genetic diversity, we computed the long‐term environmental suitability of each species’ sites with the harmonic mean of the contemporary (1979–2013) and historical environmental suitability (300–17,000 years ago) data at 10 × 10 km resolution. Harmonic means were used because they penalize sites with large variation in environmental suitability over time, and such sites would be expected to harbor less genetic diversity than temporally stable sites (Carnaval et al. 2009; Abellán & Svenning 2014). For broad‐scale genetic diversity, we used geographic distances (i.e., Euclidean distances with the projected coordinate system) and resistance distances derived with Circuitscape (version 4.0.5) (McRae et al. 2008) from inverse maps of contemporary environmental suitability (1979–2013) at 1 × 1 km resolution. To avoid numerical instability issues, contemporary environmental suitability maps were clamped to $1\;\times 10^{-5}$ prior to calculating their inverse.

### Spatial Prioritizations

We generated prioritizations to simulate conservation decisions based on different approaches. Because species had different sites and spatially interpolating data would have biased our analyses by introducing artificial spatial autocorrelation, all prioritizations were generated for each species separately. Specifically, prioritizations were generated by selecting a set of sampling sites—not 10 × 10 km grid cells—that maximized some measure of performance subject to a limit on the number of selected sites (similar to Faith & Walker 1996).

We generated prioritizations to examine surrogates for site‐level genetic diversity (Supporting Information). For each species, we generated a series of prioritizations by incrementing the number of selected sites from 1 to the maximum number of sites for that species. For a given number of sites, we generated a prioritization by selecting the set of sites with the top ranked scores of allelic richness. In a similar manner, we generated prioritizations with the long‐term environmental suitability scores. We also generated 1,000 random solutions for each incremental number of sites for each species to evaluate efficiency (per standard practices) (e.g., Ponce‐Reyes et al. 2014; Sutcliffe et al. 2015). Prioritizations were then evaluated in terms of the proportion of selected sites with high levels of allelic richness (≥80th percentile threshold) compared with the total number of sites with these levels of genetic diversity. To ensure that our results were not overly sensitive to this particular threshold, we also conducted a sensitivity analysis in which we examined performance with 2 other thresholds (i.e., ≥70th and ≥90th percentiles) (Supporting Information).

We also generated prioritizations to examine surrogates for broad‐scale genetic diversity (Supporting Information). These prioritizations aimed to identify a set of sites that secure a representative sample of the diversity between sites based on pairwise distances (i.e., genetic, geographic, or resistance distances). They were generated with the environmental diversity formulation of the reserve‐selection problem (Faith & Walker 1996) (Supporting Information) and solved to optimality with Gurobi (version 8.1.0) (Gurobi Optimization, LLC 2018). For each species, we generated a series of optimal prioritizations—each containing a different number of sites ranging from 1 to the maximum number of sites—based on the genetic distances. In a similar manner, we then generated a series of prioritizations based on geographic distances and a series of prioritizations based on resistance distances. We also generated 1,000 random solutions per incremental number of sites per species. Prioritizations were then evaluated according to the percentage of broad‐scale genetic diversity they secured based on genetic distances (Hanson et al. 2018). Because particularly poor prioritizations can yield negative percentages (akin to negative $R^{2}$ statistics), values were clamped to 0 to facilitate statistical analyses.

### Statistical Analyses

One‐sided Spearman's rank correlation tests were used to test for positive associations between the allelic richness and long‐term environmental suitability of sites for each species. Maximum likelihood population effects (MLPE) models were fitted to genetic distances (Jost's *D*) between sites to examine isolation by distance and resistance processes (Clarke et al. 2002), and model fits were assessed with $R^{2}\beta$ statistics (Edwards et al. 2008). To analyze the performance of prioritizations, generalized linear mixed‐effects models were fitted with predictor variables indicating the number of selected sites in a given prioritization, the approach used to generate it, and the interaction between these effects. Because performances were expressed as proportions, these models were fitted with logit link functions. Prediction intervals (95%) for these models were then estimated with mean estimates from 10,000 simulations. Post hoc analyses (*Z* tests) were used to determine whether the surrogate‐based prioritizations performed better than the randomly generated prioritizations when analyzing all species together. Analyses were completed with the lme4, merTools, multcomp, and r2glmm R packages (Hothorn et al. 2008; Bates et al. 2015; Jaeger 2017; Knowles & Frederick 2019).

## Results

**Figure 1:**
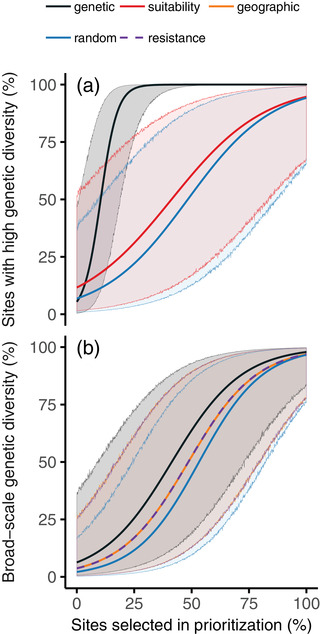
Performance of prioritizations for representing (a) high site‐level genetic diversity and (b) broad‐scale genetic diversity (estimated with Jost's *D*) (shading, 95% prediction intervals for modeled estimates of performance). Curves show modeled performance of prioritizations based on genetic data and potential surrogate‐based approaches, including long‐term environmental suitability, geographic distances, and resistance distances. They also show the performance of prioritizations generated by randomly selecting sites. In (b) lines for geographic and resistance distances are almost entirely overlapping.

Prioritizations based on long‐term environmental suitability had much poorer performance than those based on genetic data (Fig. 1a) (Supporting Information). For instance, prioritizations based on long‐term environmental suitability secured, on average, 66.34% (SD 0.22) of the sites with high site‐level genetic diversity compared with prioritizations based directly on genetic data. In the worst case, a prioritization generated for *Calotriton asper* based on long‐term environmental suitability contained 55% of its sites and none of them had high site‐level genetic diversity. The overall poor performance of this approach was likely due to the fact that long‐term environmental suitability was significantly positively correlated with site‐level genetic diversity for only 1 species (Supporting Information).

Prioritizations based on long‐term environmental suitability did not perform significantly better than randomly establishing conservation priorities when analyzing all species together (*Z* = −0.47, *p* = 0.64). They only secured, on average, 15.81% (SD 0.38) more sites with high genetic diversity than randomly generated prioritizations—a mere fraction of the performance improvement of genetic‐based prioritizations (mean 80.41% [SD 0.25]) (Supporting Information). These surrogate‐based prioritizations performed, on average, worse than random for 3 species (*Alytes obstetricans*, *Calotriton asper*, and *Chioglossa lusitanica*). When considering only prioritizations containing a realistic fraction of sites for real‐world planning (i.e., 0–25% of the sites for a given species), prioritizations based on long‐term environmental suitability had, on average, 4.35% (SD 0.32) worse performance than randomly generated prioritizations.

Prioritizations based on geographic and resistance distance data secured less broad‐scale genetic diversity than those based on genetic data (Fig. 1b) (Supporting Information). Spreading conservation priorities evenly across the geographic distribution of sites secured, on average, 81.76% (SD 0.29) of the broad‐scale genetic diversity secured by prioritizations based on genetic data. In a similar manner, spreading conservation priorities evenly across potential dispersal barriers secured, on average, 81.7% (SD 0.29) of the broad‐scale genetic diversity secured by prioritizations based on genetic data. These results are likely due to statistically significant isolation by distance and isolation by resistance processes that were detected for most species ($R^{2}\beta$ mean = 0.33 [SD 0.19] and $R^{2}\beta$ mean = 0.2 [SD 0.16] respectively) (Supporting Information).

Prioritizations based on geographic and resistance distances secured, on average, 44.31% (SD 0.29) and 41.95% (SD 0.29) (respectively) more broad‐scale genetic diversity than randomly generated prioritizations across all species (Supporting Information). They had much better performance (i.e., mean performance improvement ≥70%) than randomly generated prioritizations for 1 species (*Alytes dickhilleni*) and had near‐equivalent performance (i.e., mean performance improvement ≤ 10%) for 2 species (*Iberolacerta monticola* and *Pleurodeles waltl*). As a consequence, they did not significantly outperform the randomly generated prioritizations when analyzing all species together (geographic distances: *Z* = −0.39, *p* = 0.9; resistance distances: *Z* = −0.37, *p* = 0.91). Furthermore, when considering only prioritizations containing a realistic fraction of sites (i.e., 0–25% of the sites for a given species), prioritizations based on geographic and resistance distances secured, on average, 81.31% (SD 1.12) and 72.59% (SD 1.08) (respectively) more broad‐scale genetic diversity than random.

## Discussion

Genetic data provide insights for conserving biodiversity (Allendorf 2017). Because genetic data require resources to obtain (Puckett 2017), we investigated surrogate‐based approaches for securing genetic diversity when genetic data are not available. In Iberia none of our surrogate‐based approaches yielded prioritizations that performed as well as those based on genetic data. Prioritizations based on geographic and resistance distances generally performed better than randomly selecting reserves, so we recommend these surrogates when genetic data are not available and rapid implementation is vital. Conversely, prioritizations based on long‐term environmental suitability did not perform better than random, so we do not recommend such data as a surrogate for site‐scale genetic diversity at this point. Our findings further highlight the capacity for genetic data to inform conservation decisions (Thomassen et al. 2011; Beger et al. 2014; Hanson et al. 2019). In some cases, genetic data may already be available—meaning that genetic diversity can be integrated into conservation plans for little cost.

We found little evidence to suggest that long‐term environmental suitability is an effective surrogate for site‐level genetic diversity in conservation planning. This result contrasts with previous studies in which substantial site‐level genetic diversity in climatic refugia was detected (Comps et al. 2001; Carnaval et al. 2009; Abellán & Svenning 2014). One explanation is that our estimates of long‐term environmental suitability were not derived over a sufficiently long period. For example, although the northernmost populations of *Chioglossa lusitanica* (288 samples) and *Emys orbicularis occidentalis* (368 samples) have relatively low allelic richness (likely) due to rapid postglacial expansion (Sequeira et al. 2008; Pereira et al. 2018), our estimates could have underestimated the long‐term environmental suitability of such places because they did not account for conditions during glacial periods. Indeed, studies on other Iberian species have detected positive correlations between genetic diversity and long‐term environmental suitability when considering conditions during glacial periods (e.g., Martínez‐Freiría et al. 2015). Another possibility is that our estimates of long‐term environmental suitability did not account for historical barriers (e.g., rivers) or contemporary threatening processes (e.g., habitat fragmentation). Additionally, isolation by distance and resistance processes were detected that could limit this potential surrogate. Another explanation, as observed in *Alytes cisternasii* (225 samples) (Gonçalves et al. 2009), is that hybrid (admixed) populations of genetic lineages can have high site‐level genetic diversity. In such cases, sites with low long‐term environmental suitability could have high site‐level genetic diversity—the antithesis to our initial expectations. Because multiple mechanisms could underpin our results, further research is needed to provide more detailed recommendations.

Geographic and resistance distances were not highly effective surrogates for representing broad‐scale genetic diversity in most of our studied species. Within our study species, distant populations can have more similar genetic characteristics than nearby populations depending on the genetic lineage to which they belong (e.g., Gonçalves et al. 2009; Maia‐Carvalho et al. 2018; Valbuena‐Ureña et al. 2018), admixed populations can exhibit distinct combinations of genetic characteristics (Sequeira et al. 2008; Gonçalves et al. 2009; Pereira et al. 2018), and—particularly for amphibian species with limited dispersal abilities—populations can exhibit fine‐scale subdivision within genetic lineages (Sequeira et al. 2008; Pereira et al. 2018; Valbuena‐Ureña et al. 2018). As a consequence—despite statistically significant patterns of isolation by distance and resistance—our prioritizations based on geographic and resistance distances often missed genetically distinct groups of populations. This explanation reconciles our results with previous studies showing that geographic distances are effective surrogates for genetic diversity between sites (Ponce‐Reyes et al. 2014; Hanson et al. 2017) because these studies investigated systems in which isolation by distance processes are expected to be strong. Although our findings suggest that geographic distances may not be highly effective for species with complex phylogeographic histories, they could still be effective in systems that have strong isolation by distance processes (e.g., island networks). Moreover, these surrogate‐based approaches performed better than randomly selected sites, so geographic and resistance distances may be useful when little is known about species’ patterns of genetic diversity.

Our findings are limited to the extent that our microsatellite markers approximate genome‐wide patterns of genetic diversity. Microsatellite markers have been used extensively to assess site‐level (e.g., population diversity) and broad‐scale (e.g., genetic structure across populations) patterns of genetic diversity (Allendorf 2017). As such, large‐scale microsatellite data sets are becoming increasingly available which could help inform conservation decisions (Lawrence et al. 2019). Although they are especially well suited for characterizing recent demographic processes (10–100 generations ago), microsatellite markers can fail to reflect biologically meaningful patterns of genetic differentiation between populations, genome‐wide patterns of genetic diversity, and adaptive genetic diversity (Hedrick 1999; Selkoe & Toonen 2006; Väli et al. 2008). Despite this, several studies from which we obtained data noted congruence between the spatial patterns of microsatellite genetic diversity and hypotheses of geographic range expansion (Pereira et al. 2018), postglacial recolonization patterns (Sequeira et al. 2008; Ferchaud et al. 2015), and climatic refugia (Gutiérrez‐Rodríguez et al. 2017a). Additionally, they found strong similarities between the spatial patterns of genetic diversity revealed by microsatellite markers and mitochondrial DNA (Sequeira et al. 2008; Gonçalves et al. 2009; Gutiérrez‐Rodríguez et al. 2017a; Maia‐Carvalho et al. 2018).

Our study has further limitations. First, our findings are limited to the specific microsatellite markers, environmental data, genetic diversity metrics, and surrogate metrics we examined. Second, our sample sizes were relatively limited (Sánchez‐Montes et al. 2017), which may have affected the performance of the potential surrogate‐based approaches. Future studies could build on this by spatially interpolating estimates of genetic diversity across species’ entire geographic ranges, although they will need to carefully account for spatial autocorrelation. Third, although our environmental niche models adequately predicted the species’ contemporary distributions (Supporting Information), our estimates of long‐term environmental suitability and landscape resistance could be inaccurate if important factors are missing (e.g. land cover, microhabitat conditions). Fourth, we were unable to assess surrogates in a multispecies planning context because species were sampled at different locations. Fifth, further research is needed to explore surrogates for adaptive genetic diversity with other markers. Sixth, further work is needed to explore surrogates for other metrics derived from genetic data (e.g., phylogeographic hotspots, effective population size). Finally, our prioritizations lack details to directly inform land‐use policy within the study area.

We outline considerations for effectively incorporating genetic data into conservation planning exercises. Genetic data should ideally be available for all species of interest at each candidate site. They should ideally be produced with genomic scans (e.g., single nucleotide polymorphisms). If such data do not exist, microsatellite markers and mitochondrial DNA could be used. Additionally, value of information analyses could help optimize data collection (Grantham et al. 2008). Furthermore, statistically validated spatial models could be used to predict the genetic compositions of missing sites (e.g., allelic richness, genetic lineage membership) (Thomassen et al. 2011; Carvalho et al. 2017). After collating genetic data, prioritizations should ideally be generated to secure site‐level and broad‐scale genetic diversity. In cases where genetic data (or modeled predictions thereof) are available at all candidate sites, broad‐scale genetic diversity can be maximized based on genetic distances between selected sites (this study) or the number of genetic variants in selected sites (Diniz‐Filho et al. 2012). If existing genetic data are too limited and additional data cannot be collected, we recommend using geographic‐based surrogates. Depending on available management actions, planners should focus on populations with low or high site‐scale genetic diversity (e.g., for supplementation or protection, respectively). Planners could achieve this by applying thresholds or allele representation targets (Beger et al. 2014; Nielsen et al. 2017). Indeed, criteria for identifying Key Biodiversity Areas involve such thresholds (IUCN 2016). Furthermore, although not explored here, prioritizations should ideally consider gene flow. Planners could incorporate gene flow with spatial penalties or constraints enforcing contiguity (Beger et al. 2014; Hanson et al. 2019).

## Supporting information

Sampling localities (Appendix S1), genetic data cleaning procedures (Appendix S2), genetic diversity metrics (Appendices S3–S6), multivariate environmental similarity surface map comparing contemporary and Last Glacial Maximum conditions (Appendix S7), contemporary environmental data (Appendix S8), historical climatic data (Appendices S9–S14), environmental niche models (Appendices S15–S20), contemporary, historic, and long‐term environmental suitability maps (Appendices S21–S29), sites with high allelic richness (Appendix S30), distance‐based reserve selection procedure (Appendix S31), relationships between the genetic diversity metrics and their supposed surrogates (Appendices S32–S34), spatial prioritizations (Appendices S35–S84), and data that underpin results for site‐scale genetic diversity (Appendices S85–S89) and broad‐scale genetic diversity (Appendices S90–S94) are available online. The authors are solely responsible for the content and functionality of these materials. Queries (other than absence of the material) should be directed to the corresponding author. Code and data (except for atlas, climatic, genetic, geographic range, and soil bedrock data) are archived in a Zenodo digital repository (Hanson et al. 2020).
