# Supplementary material for: Evaluating surrogates of genetic diversity for conservation planning
Source: Conserv Biol. 2020 Oct 22;35(2):634–42. doi: 10.1111/cobi.13602 (PMC8048567; doi:10.1111/cobi.13602)
Supplement: Supplementary file 1 — Sampling localities (Appendix S1), genetic data cleaning procedures (Appendix S2), genetic diversity metrics (Appendices S3–S6), multivariate environmental similarity surface map comparing contemporary and Last Glacial Maximum conditions (Appendix S7), contemporary environmental data (Appendix S8), historical climatic data (Appendices S9–S14), environmental niche models (Appendices S15–S20), contemporary, historic, and long‐term environmental suitability maps (Appendices S21–S29), sites with high allelic richness (Appendix S30), distance‐based reserve selection procedure (Appendix S31), relationships between the genetic diversity metrics and their supposed surrogates (Appendices S32–S34), spatial prioritizations (Appendices S35–S84), and data that underpin results for site‐scale genetic diversity (Appendices S85–S89) and broad‐scale genetic diversity (Appendices S90–S94) are available online. The authors are solely responsible for the content and functionality of these materials. Queries (other than absence of the material) should be directed to the corresponding author. Code and data (except for atlas, climatic, genetic, geographic range, and soil bedrock data) are archived in a Zenodo digital repository (Hanson et al. 2020). [file COBI-35-634-s001.pdf]

Evaluating surrogates of genetic diversity for conservation planning

Jeffrey O. Hanson[*1], *Ana Veríssimo*[1], *Guillermo Velo-Antón*[1], *Adam Marques*[1]
Miguel Camacho-Sanchez[1], *Íñigo Martínez-Solano*[1,2], *Helena Gonçalves*[1,3],
Fernando Sequeira[1], *Hugh P. Possingham*[4,5], *Silvia B. Carvalho*[1]

[1] *CIBIO/InBIO, Centro de Investigação em Biodiversidade e Recursos Genéticos, Universidade do Porto, Campus de Vairão, Rua Padre Armando Quintas, nº 7 4485-661 Vairão, Portugal*

[2] *Museo Nacional de Ciencias Naturales-CSIC, Calle de José Gutiérrez Abascal, 2, 28006 Madrid, Spain*

[3] *Museu de História Natural e da Ciência, Universidade do Porto, Praça Gomes Teixeira 4099-002 Porto, Portugal*

[4] *The Nature Conservancy, Minneapolis, MN, 55415 USA*

[5] *Centre for Biodiversity and Conservation Science, School of Biological Sciences, The University of Queensland, Brisbane, QLD, 4072 Australia*

**email jeffrey.hanson@uqconnect.edu.au*

01 September 2020

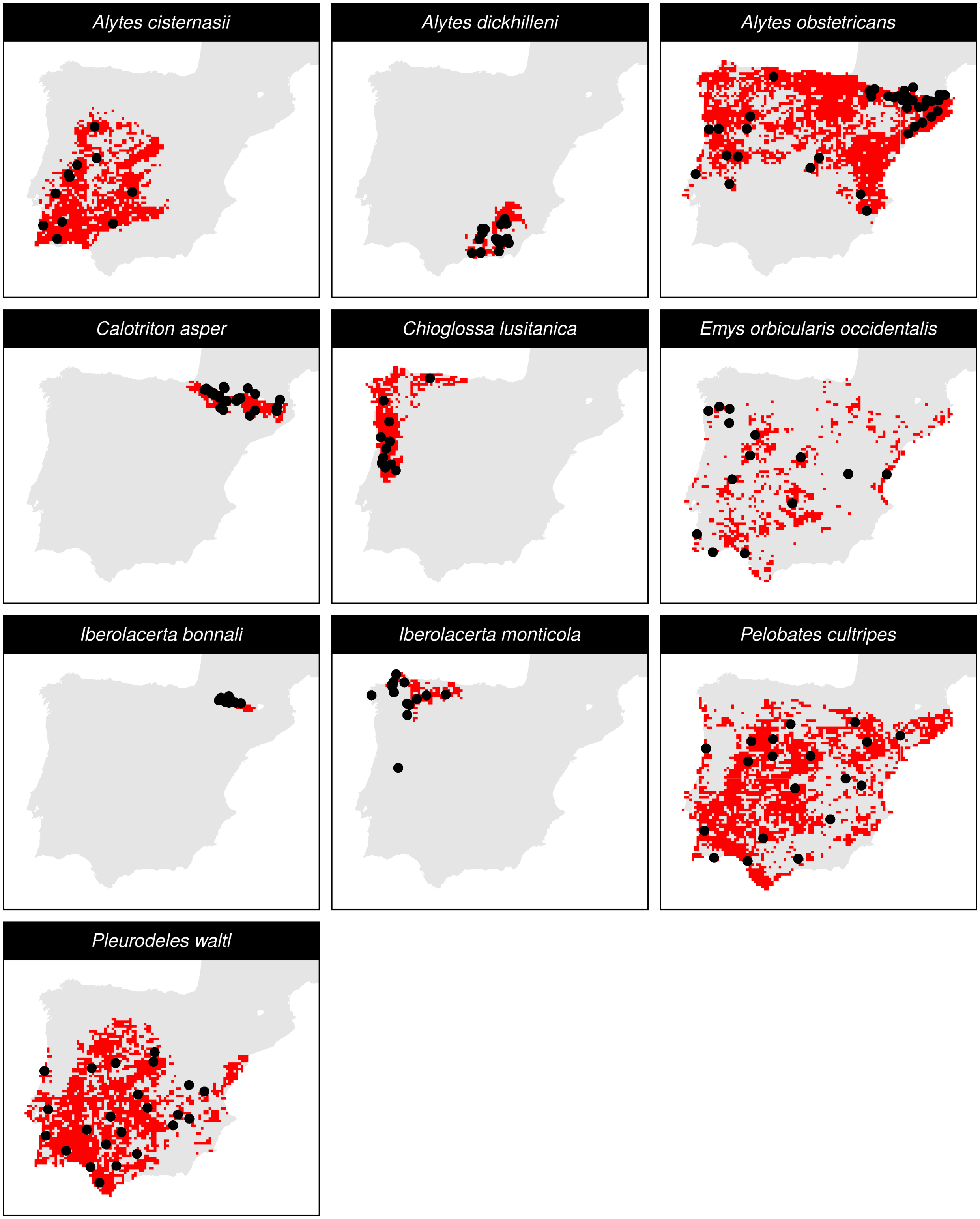

Appendix S1: Spatial distribution (red) and sampling localities (black points) for each species. Note that the spatial distributions are based on atlas data.

Appendix S2: We prepared the genetic data for each species separately. Specifically, samples collected outside the study area were excluded and sites with fewer than ten samples were also excluded to prevent such sites from biasing the analyses (Pereira et al. 2018). We then verified that our estimates of genetic diversity would not be biased by highly related individuals (e.g. sibling tadpoles sampled from the same pond). To achieve this, pairwise individual relatedness statistics were calculated for all studied species using the `related` `R` package. Next, for each species, the maximum relatedness statistic between pairs of sampled individuals from different sites was calculated, and this statistic was used as a threshold to identify highly related pairs of individuals from the same site. After comparing the pairwise relatedness statistics between individuals sampled from the same site to the species' threshold statistic, we found that none of the individuals sampled from the same site exceeded this threshold for any of the studied species. Furthermore, to ensure that our findings are not due to insufficient sampling or biased genetic diversity estimates, we verified that allelic richness (Appendix S3) and expected heterozygosity (Appendix S4)—an alternative site-level genetic diversity metric—were significantly correlated for nearly all species (Appendices S5 and S6).

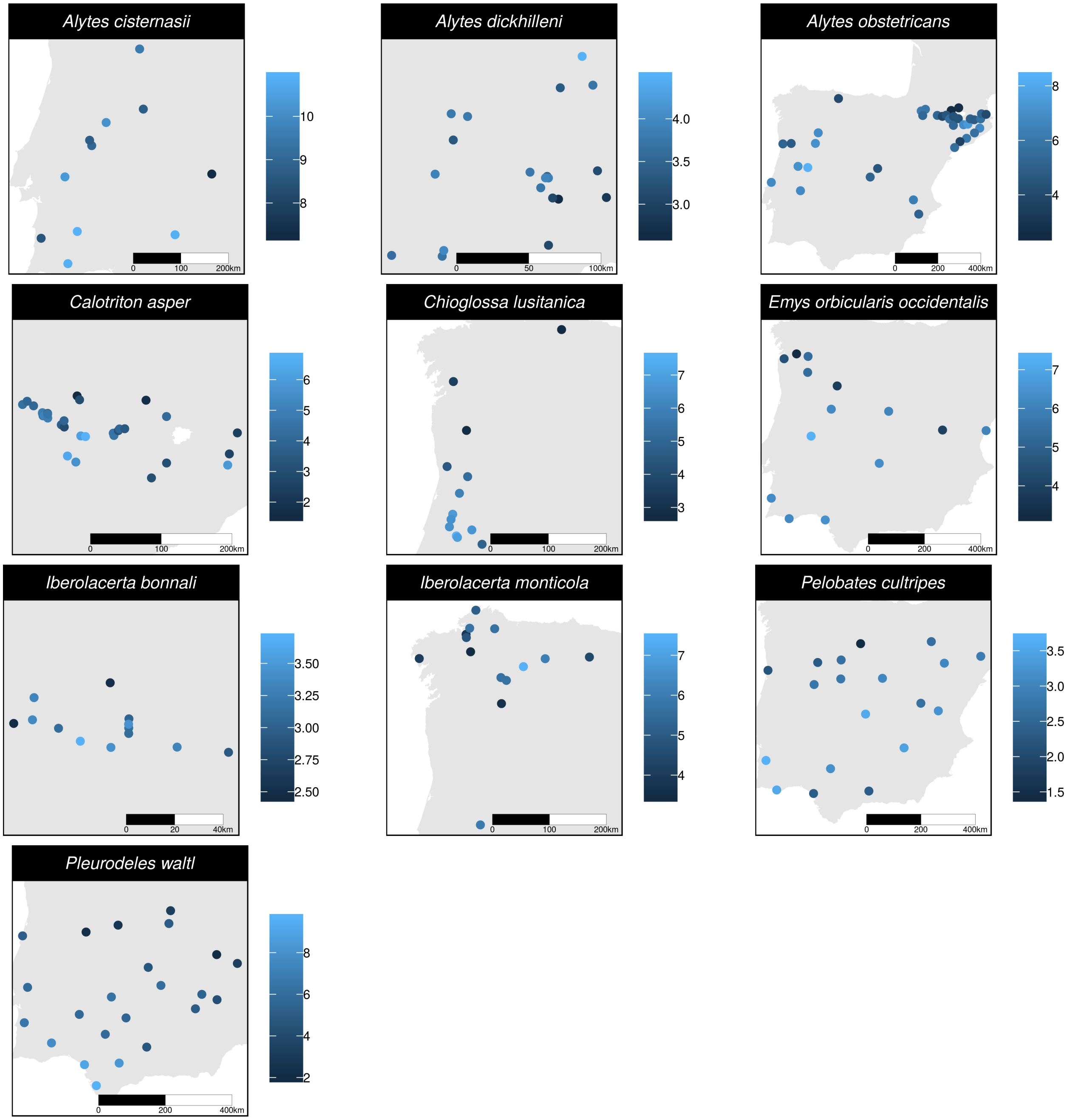

Appendix S3: Spatial distribution of allelic richness for each species. Each panel corresponds to a different species, and points denote sampling sites. The color of each point indicates the allelic richness of samples collected at the site.

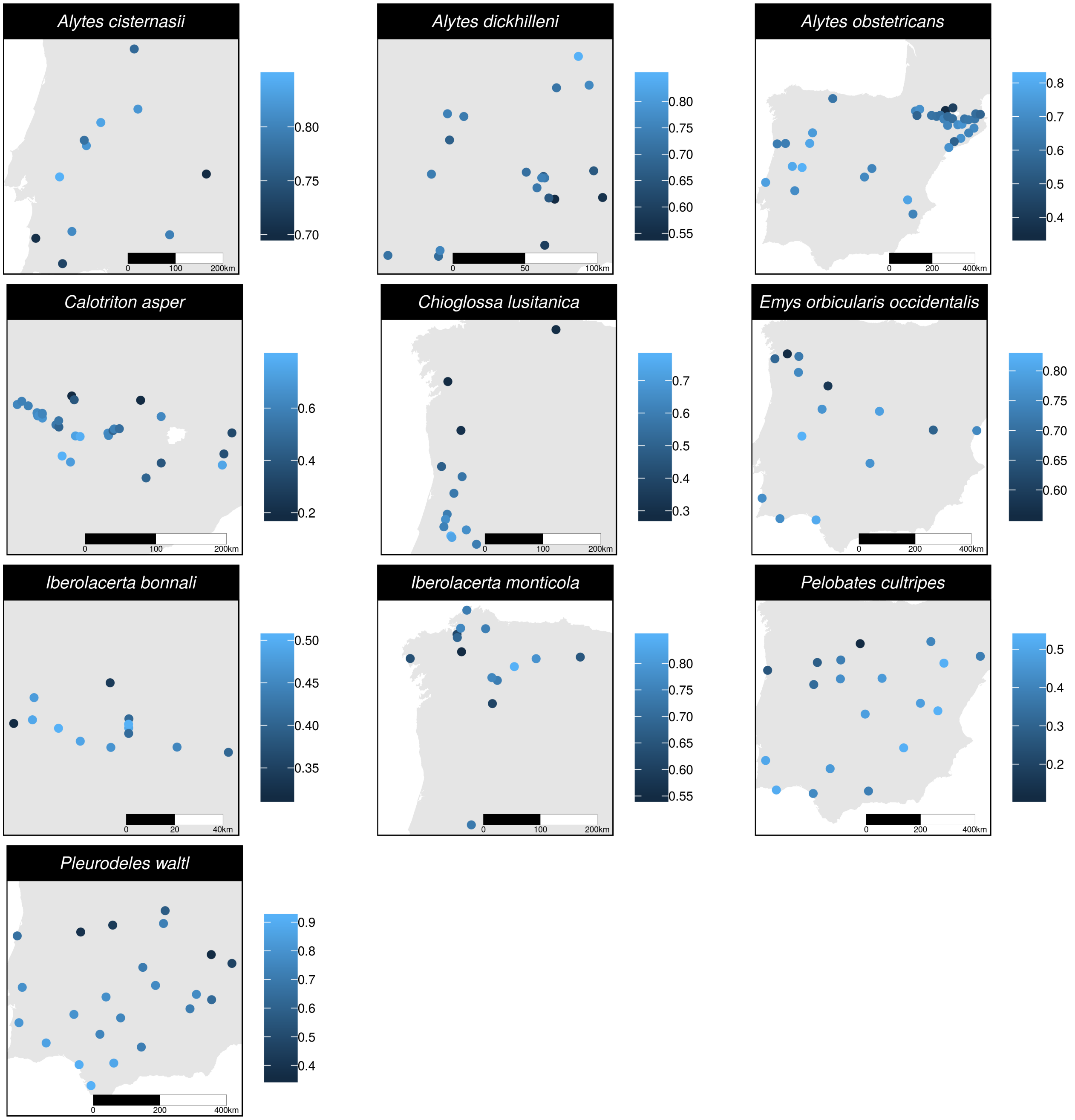

Appendix S4: Spatial distribution of expected heterozygosity for each species. Each panel corresponds to a different species, and points denote sampling sites. The color of each point indicates the expected heterozygosity of samples collected at the site.

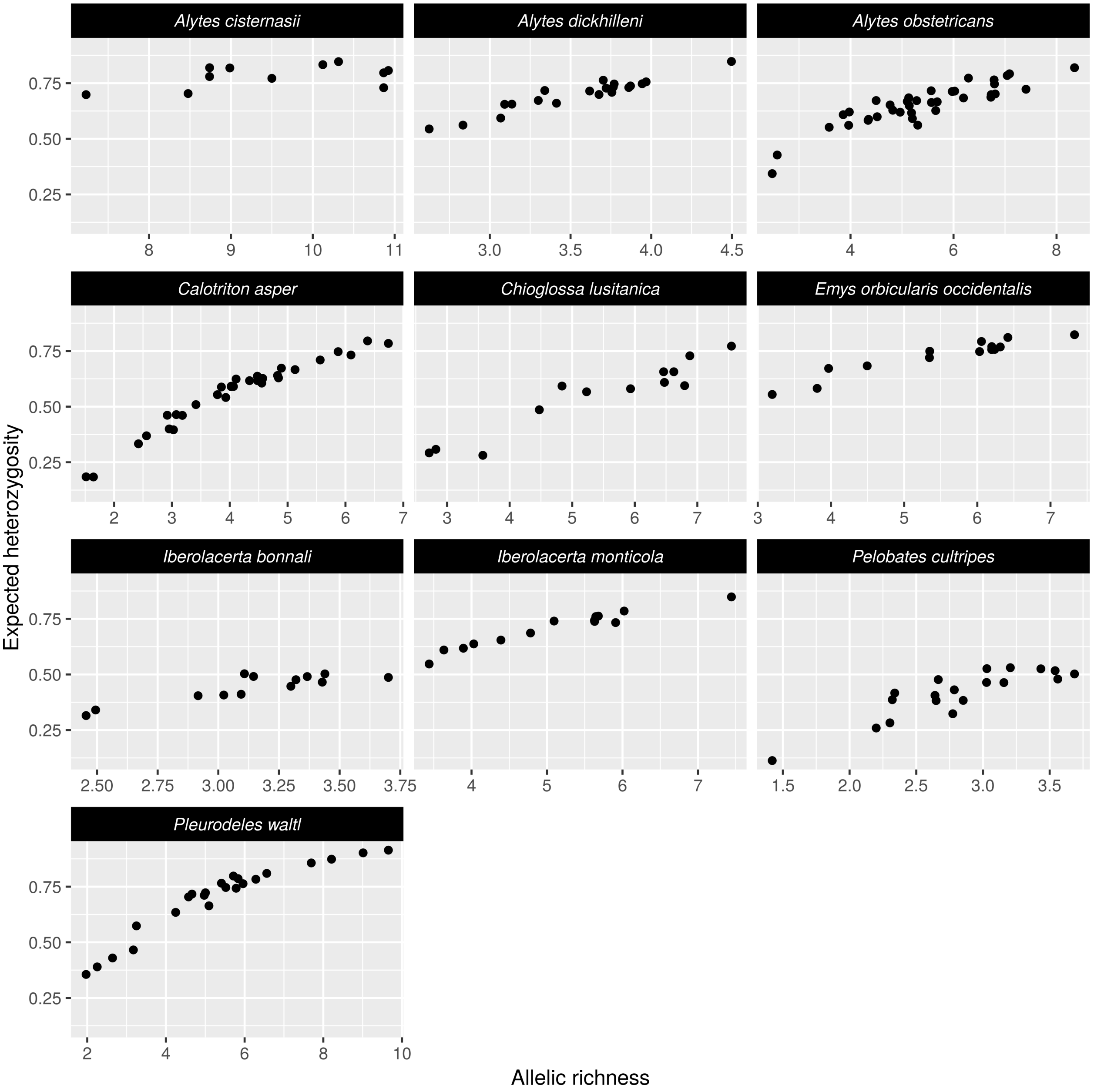

Appendix S5: The relationship between the allelic richness and the expected heterozygosity measured at a site. Each panel corresponds to a different species, and points correspond to sites.

Appendix S6: One-sided Spearman's rank correlation tests between the allelic richness and expected heterozygosity of individuals at different sites.

Species	r	Statistic	P
Alytes cisternasii	0.39	134.00	0.12
Alytes dickhilleni	0.91	122.00	$<$ **0.001**
Alytes obstetricans	0.86	1158.00	$<$ **0.001**
Calotriton asper	0.98	82.00	$<$ **0.001**
Chioglossa lusitanica	0.92	27.54	$<$ **0.001**
Emys orbicularis occidentalis	0.94	28.00	$<$ **0.001**
Iberolacerta bonnali	0.72	102.00	**0.004**
Iberolacerta monticola	0.92	36.00	$<$ **0.001**
Pelobates cultripes	0.83	192.00	$<$ **0.001**
Pleurodeles waltl	0.97	70.00	$<$ **0.001**

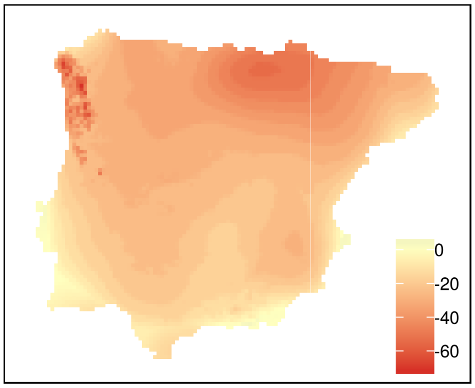

Appendix S7: Multivariate environmental similarity surface (MESS; Elith et al. 2010) map showing differences between contemporary climatic conditions (1979–2013) and climatic conditions during the Last Glacial Maximum (*c.* 21 thousand years ago). Specifically, this map was generated with the same eight bioclimatic variables used to fit the environmental niche models reported in the main text (10 × 10 km resolution). Greater values denote areas with greater climatic similarity between the two time periods. Negative values denote climatic conditions that are not observed in the contemporary dataset.

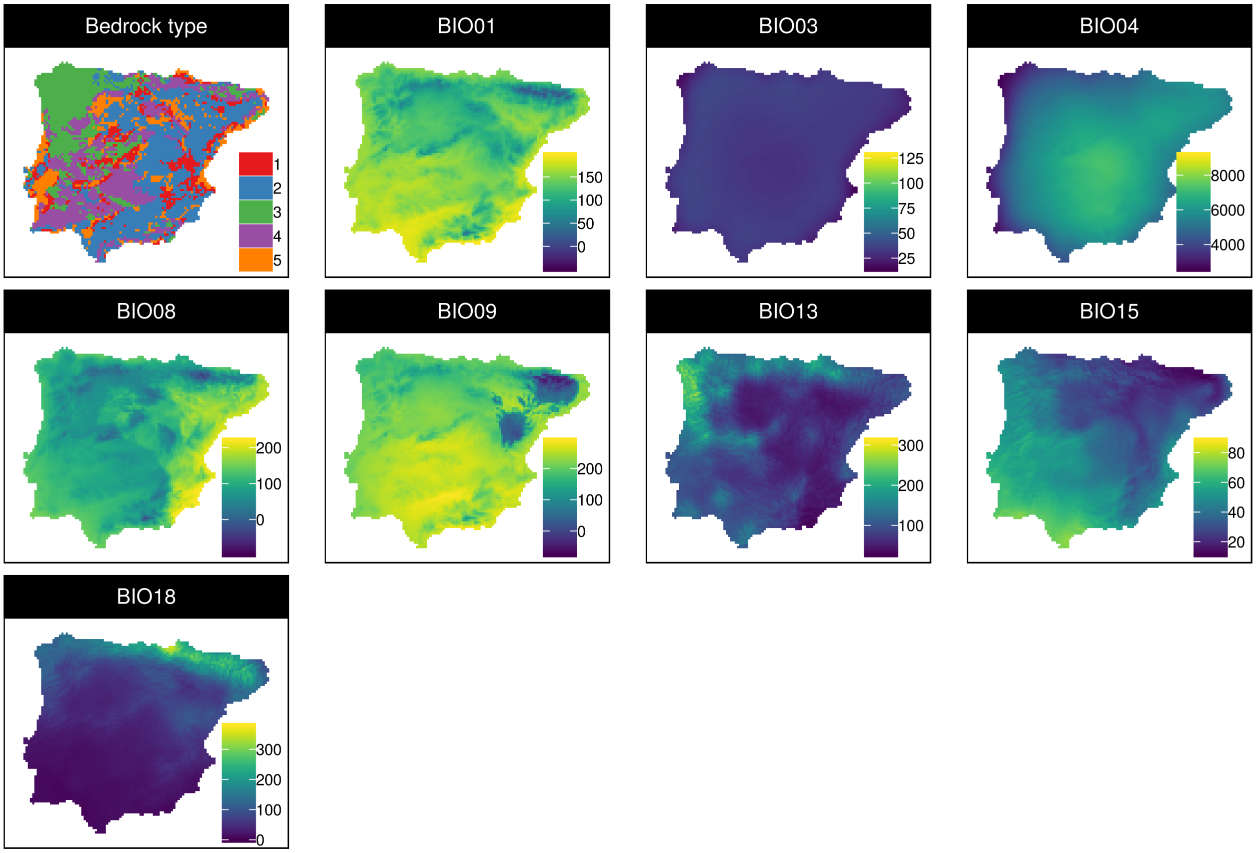

Appendix S8: Environmental variables used for fitting species distribution models and estimating contemporary environmental suitability. These variables include bioclimatic and bedrock classification data (10 × 10 km resolution). Bedrock classes are: (1) consolidated-clastic-sedimentary rocks, (2) a composite of eolian deposits and sedimentary rocks (chemically precipitated, evaporated, or organogenic or biogenic in origin), (3) igneous rocks, (4) metamorphic rocks, (5) unconsolidated deposits (alluvium, weathering residuum and slope deposits) .

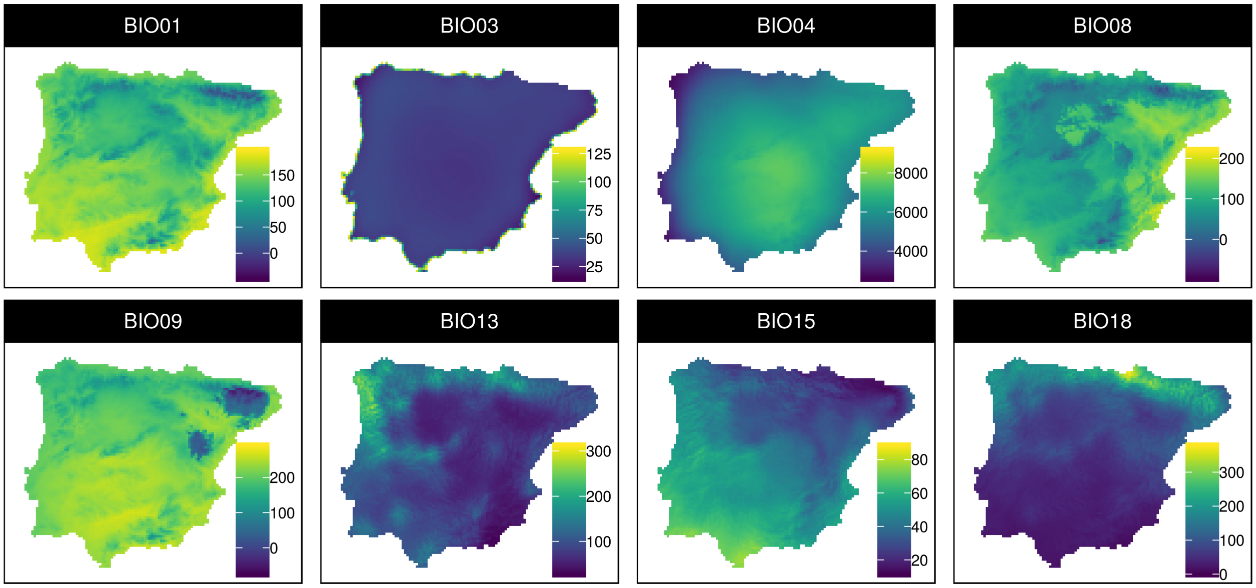

Appendix S9: Historic bioclimatic variables predicted for the late-Holocene, Meghalayan (4.2–0.3 ka; 10 × 10 km resolution).

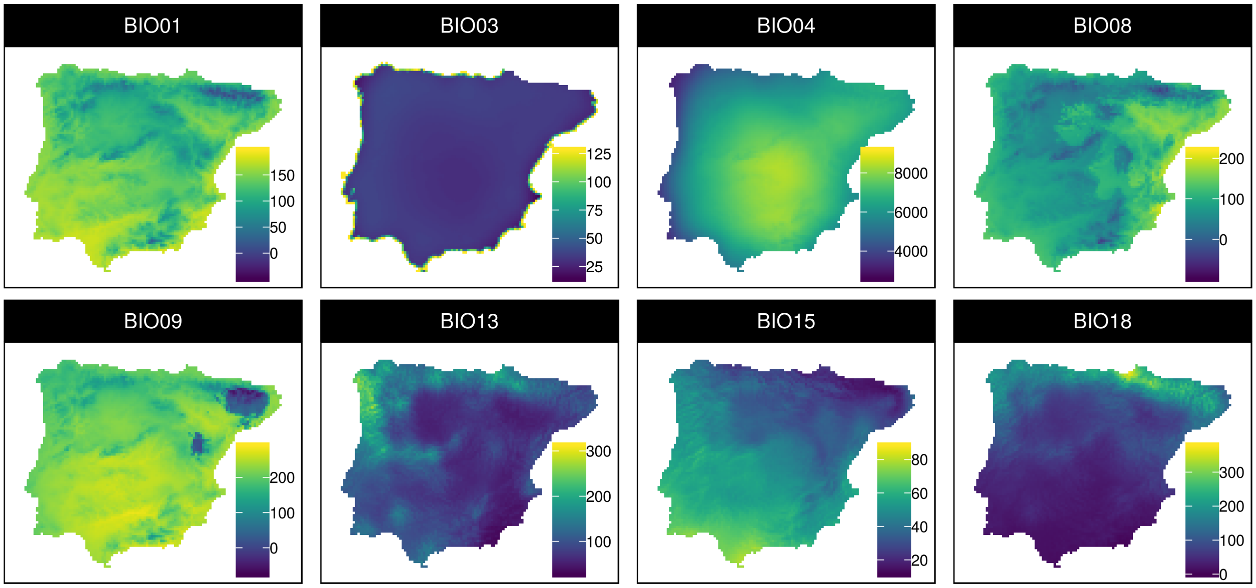

Appendix S10: Historic bioclimatic variables predicted for the mid-Holocene, Northgrippian (8.326–4.2 ka; 10 × 10 km resolution).

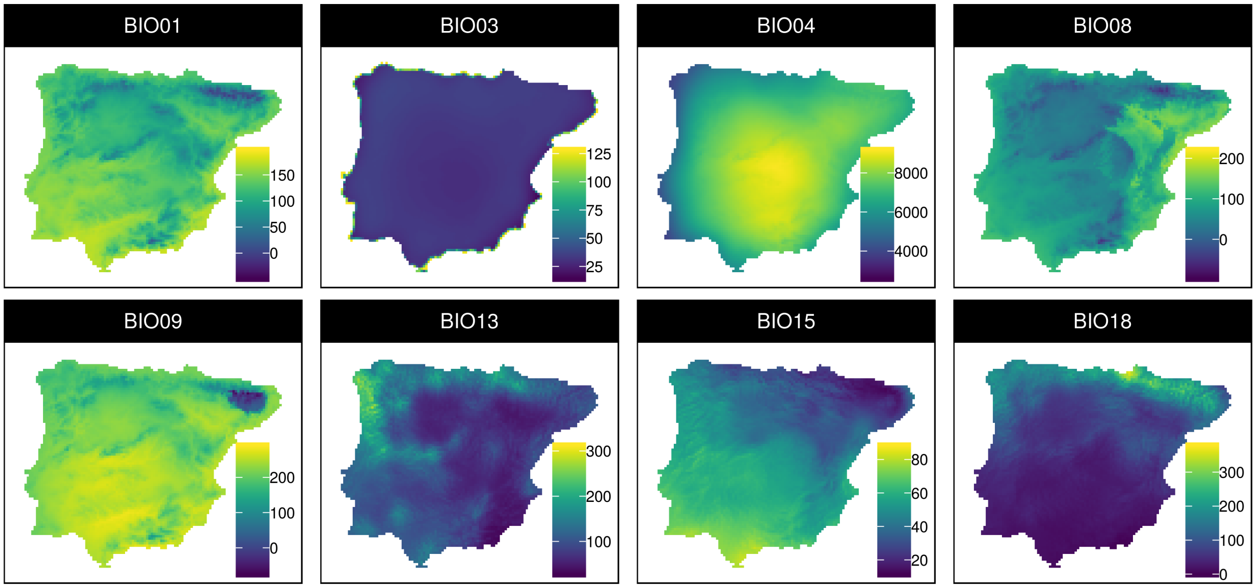

Appendix S11: Historic bioclimatic variables predicted for the early-Holocene, Greenlandian (11.7–8.326 ka; 10 × 10 km resolution).

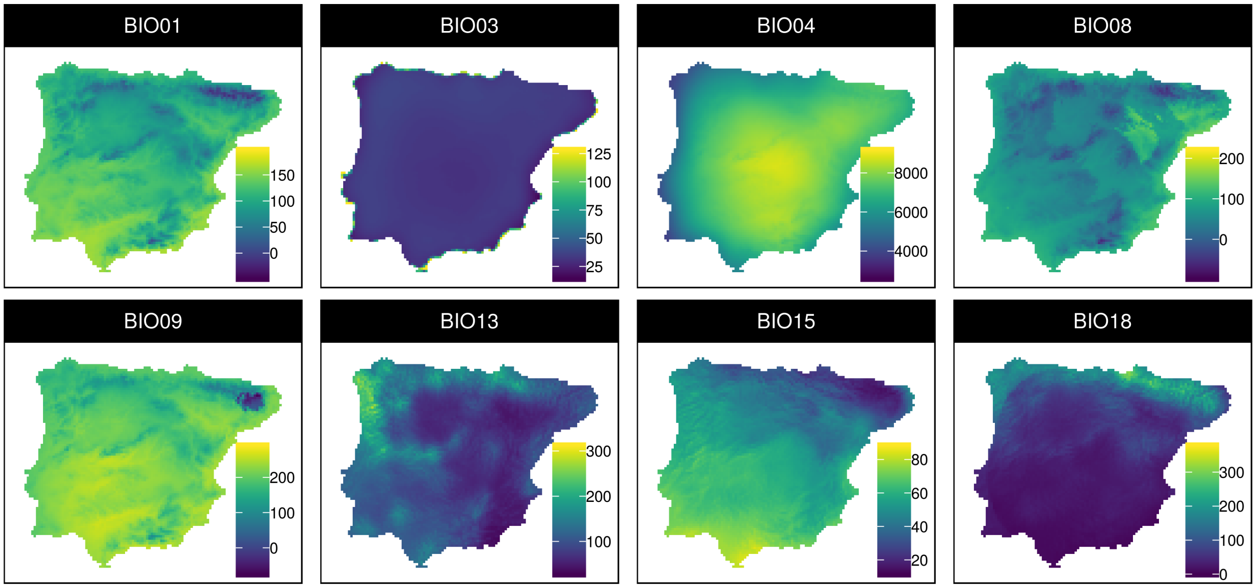

Appendix S12: Historic bioclimatic variables predicted for the Pleistocene, Younger Dryas Stadial (12.9–11.7 ka; 10 × 10 km resolution).

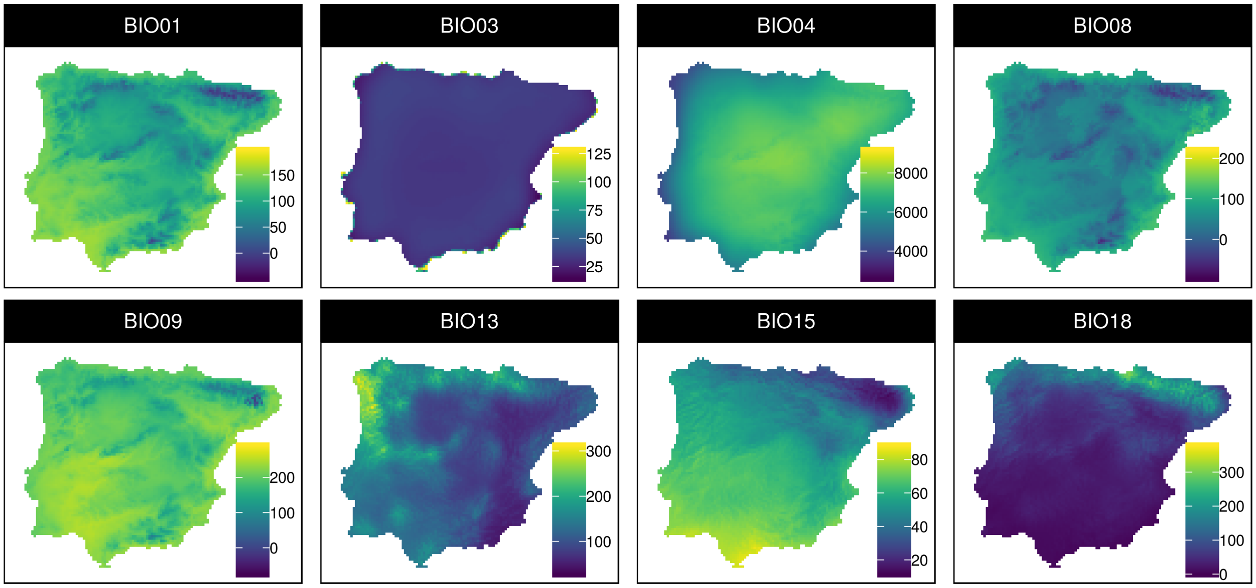

Appendix S13: Historic bioclimatic variables predicted for the Pleistocene, Bølling-Allerød (14.7–12.9 ka; 10 × 10 km resolution).

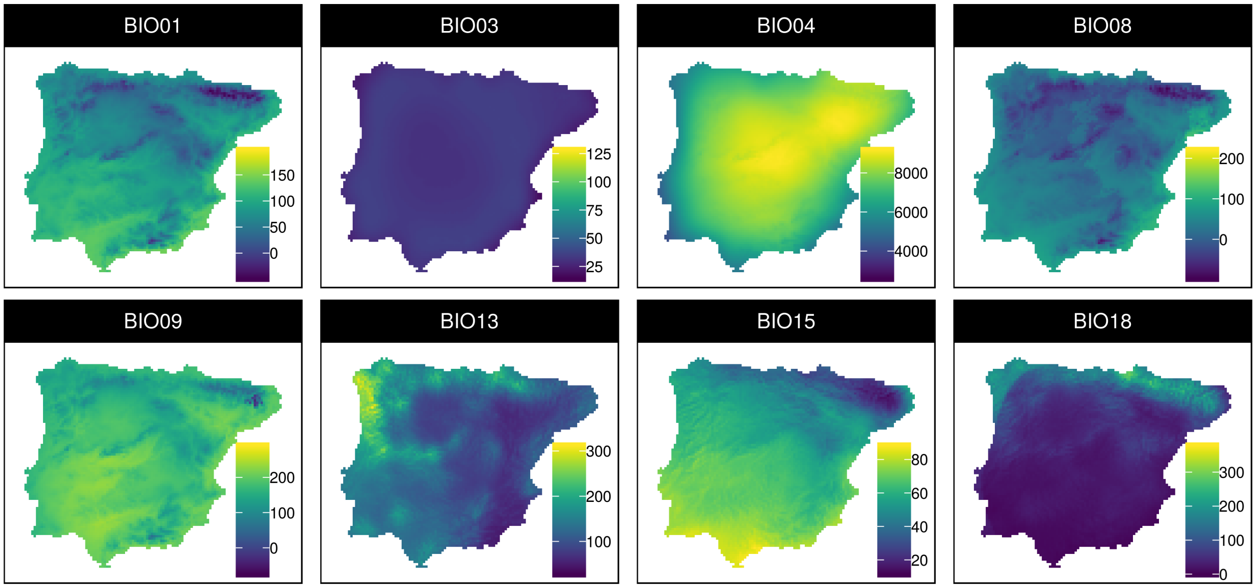

Appendix S14: Historic bioclimatic variables predicted for the Pleistocene, Heinrich Stadial 1 (17.0–14.7 ka; 10 × 10 km resolution).

Appendix S15: We prepared the atlas data (see Appendix S16 for summary statistics) for fitting environmental niche models. Specifically, we generated a presence dataset for each species using the centroids of the atlas grid cells where the species was recorded as present. We also generated an absence dataset for each species using the centroids of randomly sampled atlas grid cells where the species was not recorded. The specific number of absence grid cells for a given species was half of the total number of atlas grid cells where the species was not recorded (following Carvalho et al. 2019). Since the atlas dataset follows a regular grid and is spatially comprehensive across most of the study area, we did not explicitly account for spatial biases in sampling effort.

We then used spatial blocking (Valavi et al. 2019) to prepare the presence and absence data for model fitting (see Appendix S17 for a summary). Although 313 $\times$ 313 km blocks would be optimal given the patterns of spatial autocorrelation among the fine-scale environmental data (1 $\times$ 1 km resolution; based on the `blockCV R` package; version 2.1.1), these blocks were too large and encompassed the entire geographic range of many of the study species. Therefore, as a compromise, we used 125 $\times$ 125 km spatial blocks. The spatial blocks were then used to allocate the species' presence and absence grid cells to five folds for subsequent model fitting (using the `blockCV R` package to balance data among folds). Since these blocks were still too large for *Iberolacerta bonnali* and *Iberolacerta monticola*, we used a random procedure to allocate the presence atlas grid cells for these species to the five folds. The absence grid cells for these two species – similar to the other species – were allocated using the spatial blocks. After preparing the species' presence and absence data for model fitting, we then generated environmental niche models.

We generated environmental niche models (via the `biomod2 R` package; Thuiller et al. 2020) by fitting ensemble models to the species' presence and absence data using the contemporary environmental data (10 $\times$ 10 km resolution). Specifically, we separately fitted generalized boosted regression models (GBM), generalized additive models (GAM), artificial neural networks (ANN), and random forests (RF) using default settings. These models were trained using five-cross validation – using the previously described spatially blocked folds – and then assessed using area under the receiver operating characteristic curve (AUC) statistics calculated using the (test) data held out from each fold (Appendix S18). After fitting the models, we excluded models with AUC statistics below 0.7 to prevent poor models from weakening the ensemble models. We then used the remaining models to build an ensemble model for each species, based on the models' average predictions and weighted by their performances (AUC statistics). Next, we used AUC statistics to evaluate the ability of the ensemble models to predict the atlas presence and absence data used for model fitting (mean 0.88 AUC; ranging from 0.75 to 1; Appendix S19). Based on these statistics, we are confident that the ensemble models have sufficient performance to achieve the aims of the study.

We evaluated the strength of spatial autocorrelation among the residuals of the ensemble models (Appendix S20). Since we are only interested in estimating environmental suitability at the species' genetic sampling site locations – and not across the entire study area – we tested for patterns of spatial autocorrelation at the locations of each species' sampling sites. For each species, we calculated the residuals of its ensemble model – using the atlas presence and absence data – at the locations of its sampling sites and then tested for significant patterns of spatial autocorrelation using Moran's I statistics (via the `ape R` package; version 5.3). Since some species had sampling sites beyond the extent of the atlas data (i.e. south-western France), these sites were excluded from the spatial autocorrelation analyses. After completing the tests for each species, we applied a Bonferroni correction to correct for false positives. These analyses did not detect significant patterns of spatial autocorrelation in the model residuals for any species (all species had $P > 0.05$; Appendix S20).

Appendix S16: Total number of atlas grid cells with presences and absences for each species.

Species	Present	Absent
Alytes cisternasii	1186	4871
Alytes dickhilleni	186	5871
Alytes obstetricans	2437	3620
Calotriton asper	215	5842
Chioglossa lusitanica	428	5629
Emys orbicularis occidentalis	746	5311
Iberolacerta bonnali	33	6024
Iberolacerta monticola	165	5892
Pelobates cultripes	2381	3676
Pleurodeles waltl	1951	4106

Appendix S17: Summary of the atlas grid cells used for training and evaluating the environmental niche models with five-fold cross-validation. For a given species, for a given fold, data show the (TP) number of atlas presence grid cells used for model fitting, (TA) number of atlas absence grid cells used for model fitting, (EP) number of atlas presence grid cells used for model evaluation, and (EA) number of atlas absence grid cells used for model evaluation.

Species	Fold	TP	TA	EP	EA
Alytes cisternasii	1	1056	2031	130	404
	2	912	1844	274	591
	3	1035	1911	151	524
	4	715	1927	471	508
	5	1026	2027	160	408
Alytes dickhilleni	1	134	2417	52	518
	2	154	2256	32	679
	3	175	2352	11	583
	4	168	2412	18	523
	5	113	2303	73	632
Alytes obstetricans	1	2006	1522	431	288
	2	2003	1306	434	504
	3	1691	1555	746	255
	4	1979	1337	458	473
	5	2069	1520	368	290
Calotriton asper	1	113	2338	102	583
	2	195	2442	20	479
	3	172	2288	43	633
	4	175	2270	40	651
	5	205	2346	10	575
Chioglossa lusitanica	1	334	2182	94	632
	2	374	2218	54	596
	3	394	2241	34	573
	4	313	2266	115	548
	5	297	2349	131	465
Emys orbicularis occidentalis	1	632	2206	114	449
	2	614	2055	132	600
	3	673	2039	73	616
	4	461	2070	285	585
	5	604	2250	142	405
Iberolacerta bonnali	1	26	2426	7	586
	2	28	2368	5	644
	3	29	2537	4	475
	4	25	2334	8	678
	5	24	2383	9	629
Iberolacerta monticola	1	124	2406	41	540
	2	138	2180	27	766
	3	141	2464	24	482
	4	134	2361	31	585
	5	123	2373	42	573

Species	Fold	TP	TA	EP	EA
Pelobates cultripes	1	2021	1538	360	300
	2	1879	1394	502	444
	3	2008	1399	373	439
	4	1621	1480	760	358
	5	1995	1541	386	297
Pleurodeles waltl	1	1722	1755	229	298
	2	1498	1597	453	456
	3	1640	1522	311	531
	4	1410	1661	541	392
	5	1534	1677	417	376

Appendix S18: Summary of environmental niche models. Models were fitted using multiple methods (method) and five-fold cross-validation (fold; identifiers correspond to Appendix S16). They were evaluated using area under the receiver operating characteristic curve (AUC) statistics that were calculated using the data held out from each fold. Missing values indicate that issues were encountered while fitting a given model, and so the model was not used for ensemble modelling.

Species	Method	Fold	*AUC*
Alytes cisternasii	GBM	1	–
		2	0.858
		3	0.955
		4	0.882
	GAM	1	0.955
		2	0.846
		3	0.888
		4	0.877
	ANN	1	0.958
		2	0.837
		3	0.936
		4	0.893
	RF	1	0.933
		2	0.845
		3	0.953
		4	0.885
Alytes dickhilleni	GBM	1	0.828
		2	0.934
		3	0.926
		4	0.917
	GAM	1	0.698
		2	0.936
		3	0.957
		4	0.966
	ANN	1	0.7
		2	0.928
		3	0.914
		4	0.979
	RF	1	0.87
		2	0.984
		3	0.974
		4	0.888
Alytes obstetricans	GBM	1	–
		2	0.892
		3	0.674
		4	0.936
	GAM	1	0.87
		2	0.898
		3	0.732
		4	0.948

Species	Method	Fold	*AUC*
	ANN	1	–
		2	0.899
		3	0.657
		4	0.915
	RF	1	0.927
		2	0.9
		3	0.729
		4	0.935
Calotriton asper	GBM	1	–
		2	1
		3	0.991
		4	0.979
	GAM	1	0.89
		2	0.999
		3	0.991
		4	0.835
	ANN	1	0.958
		2	0.999
		3	0.992
		4	0.961
	RF	1	0.98
		2	1
		3	0.992
		4	0.992
Chioglossa lusitanica	GBM	1	0.992
		2	0.926
		3	0.964
		4	0.96
	GAM	1	0.987
		2	0.948
		3	0.861
		4	0.667
	ANN	1	0.992
		2	0.833
		3	0.951
		4	0.966
	RF	1	0.991
		2	0.954
		3	0.976
		4	0.955
Emys orbicularis occidentalis	GBM	1	0.771
		2	0.659
		3	0.774
		4	0.716
	GAM	1	0.748
		2	0.654

Species	Method	Fold	*AUC*
		3	0.683
		4	0.667
	ANN	1	0.71
		2	0.671
		3	0.554
		4	0.641
	RF	1	0.777
		2	0.696
		3	0.775
		4	0.747
Iberolacerta bonnali	GBM	1	0.984
		2	0.999
		3	1
		4	1
	GAM	1	0.989
		2	0.8
		3	1
		4	0.688
	ANN	1	0.988
		2	–
		3	1
		4	1
	RF	1	0.982
		2	1
		3	1
		4	1
Iberolacerta monticola	GBM	1	0.964
		2	0.996
		3	0.979
		4	0.95
	GAM	1	0.947
		2	0.988
		3	0.972
		4	0.937
	ANN	1	0.979
		2	0.997
		3	0.973
		4	0.911
	RF	1	0.959
		2	0.996
		3	0.975
		4	0.951
Pelobates cultripes	GBM	1	–
		2	0.764
		3	0.78
		4	0.698

Species	Method	Fold	*AUC*
	GAM	1	0.744
		2	0.739
		3	0.761
		4	0.586
	ANN	1	0.768
		2	0.656
		3	0.72
		4	0.576
	RF	1	0.812
		2	0.771
		3	0.782
		4	0.723
Pleurodeles waltl	GBM	1	–
		2	0.872
		3	0.942
		4	0.79
	GAM	1	0.828
		2	0.775
		3	0.94
		4	0.777
	ANN	1	0.853
		2	0.852
		3	0.911
		4	0.772
	RF	1	0.882
		2	0.877
		3	0.938
		4	0.8

Appendix S19: Performance of ensemble environmental niche models. They were evaluated using area under the receiver operating characteristic curve (AUC) statistics calculated using all of the presence and absence grid cells associated with each species.

Species	*AUC*
Alytes cisternasii	0.845
Alytes dickhilleni	0.978
Alytes obstetricans	0.789
Calotriton asper	0.989
Chioglossa lusitanica	0.955
Emys orbicularis occidentalis	0.790
Iberolacerta bonnali	1.000
Iberolacerta monticola	0.967
Pelobates cultripes	0.755
Pleurodeles waltl	0.748

Appendix S20: Spatial autocorrelation test results. The residuals of the environmental niche models at the species' sampling site locations were tested for spatial auto-correlation using Moran's I statistics. Data show the number of sites examined (n), observed statistic, expected statistic, standard deviation statistic, uncorrected P-value (P_u) and Bonferroni corrected P-value (P_c) for each species.

Species	n	Expected	Observed	Standard Deviation	P_u	P_c
Alytes cisternasii	11	-0.110	-0.100	0.100	0.92	> 0.99
Alytes dickhilleni	20	-0.057	-0.053	0.080	0.96	> 0.99
Alytes obstetricans	35	-0.033	-0.028	0.042	0.90	> 0.99
Calotriton asper	26	0.095	-0.033	0.068	0.058	0.58
Chioglossa lusitanica	13	-0.023	-0.083	0.065	0.35	> 0.99
Emys orbicularis occidentalis	14	-0.053	-0.077	0.075	0.75	> 0.99
Iberolacerta bonnali	7	-0.126	-0.083	0.045	0.34	> 0.99
Iberolacerta monticola	14	-0.085	-0.077	0.086	0.93	> 0.99
Pelobates cultripes	19	-0.027	-0.056	0.034	0.40	> 0.99
Pleurodeles waltl	22	-0.067	-0.045	0.045	0.63	> 0.99

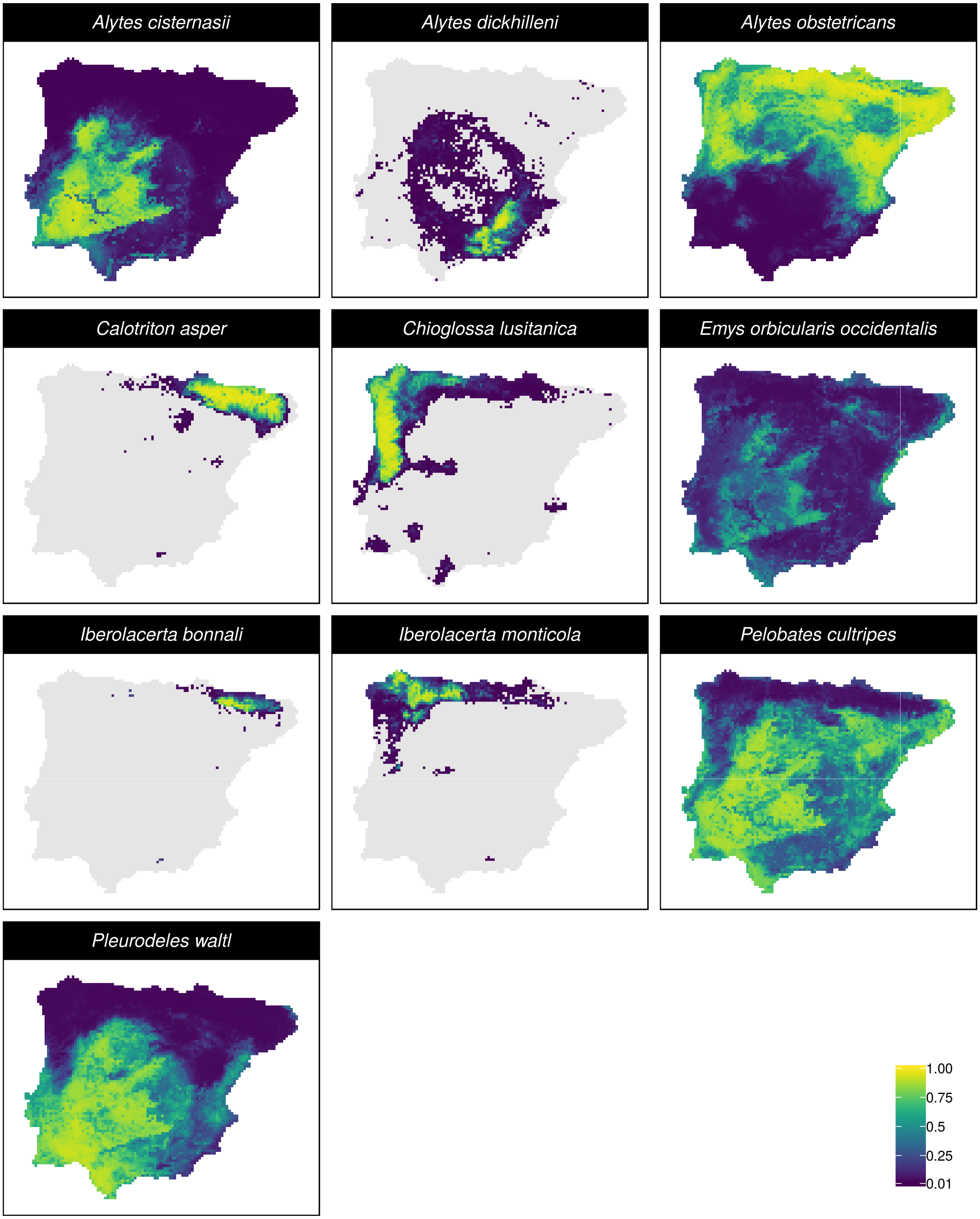

Appendix S21: Maps of contemporary environmental suitability (10 × 10 km resolution). To aid with visual interpretation, places with near-zero environmental suitability scores (less than 0.01) are shown in grey.

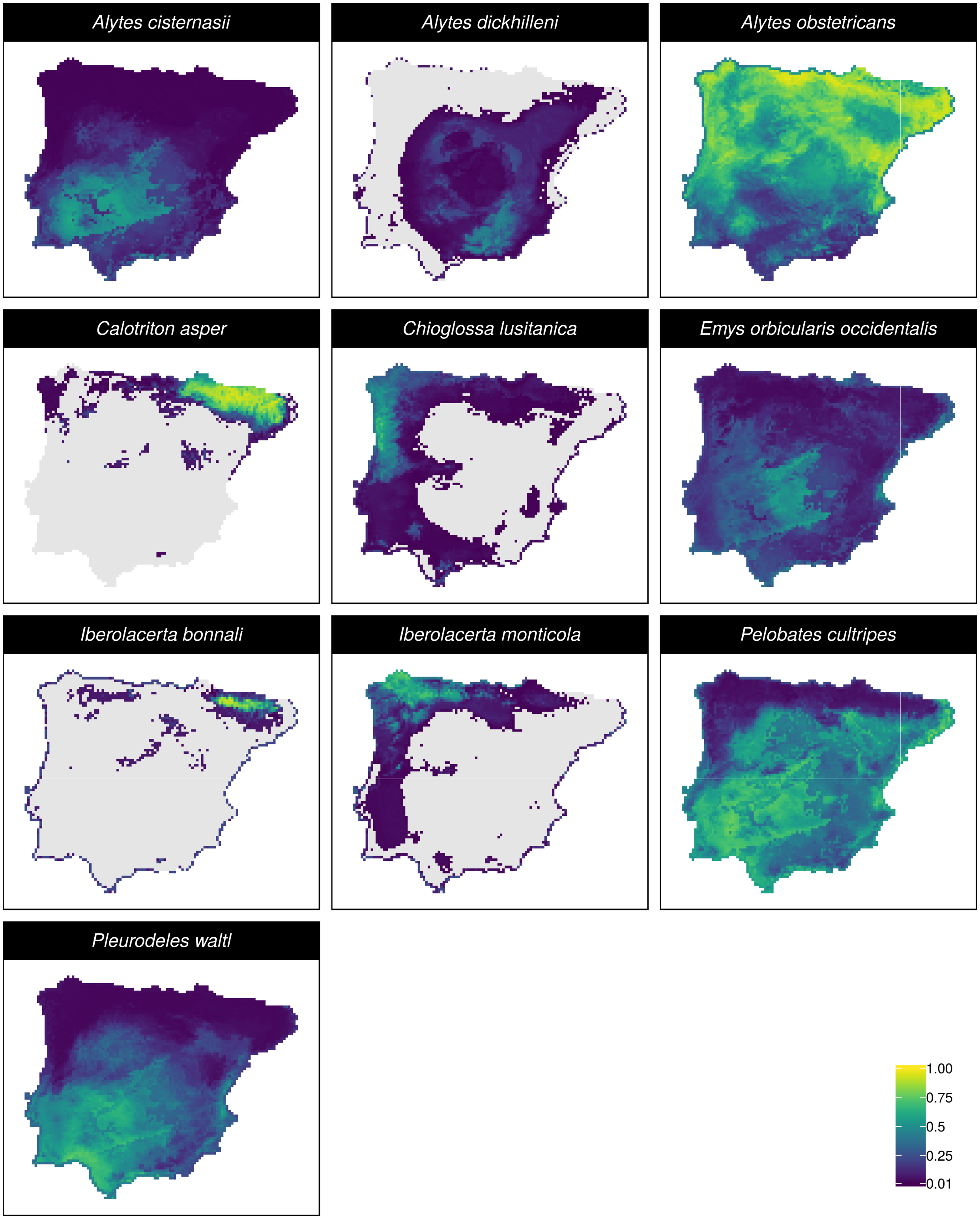

Appendix S22: Maps of historic environmental suitability predicted for the late-Holocene, Meghalayan (4.2–0.3 ka; 10 × 10 km resolution). To aid with visual interpretation, places with near-zero environmental suitability scores (less than 0.01) are shown in grey.

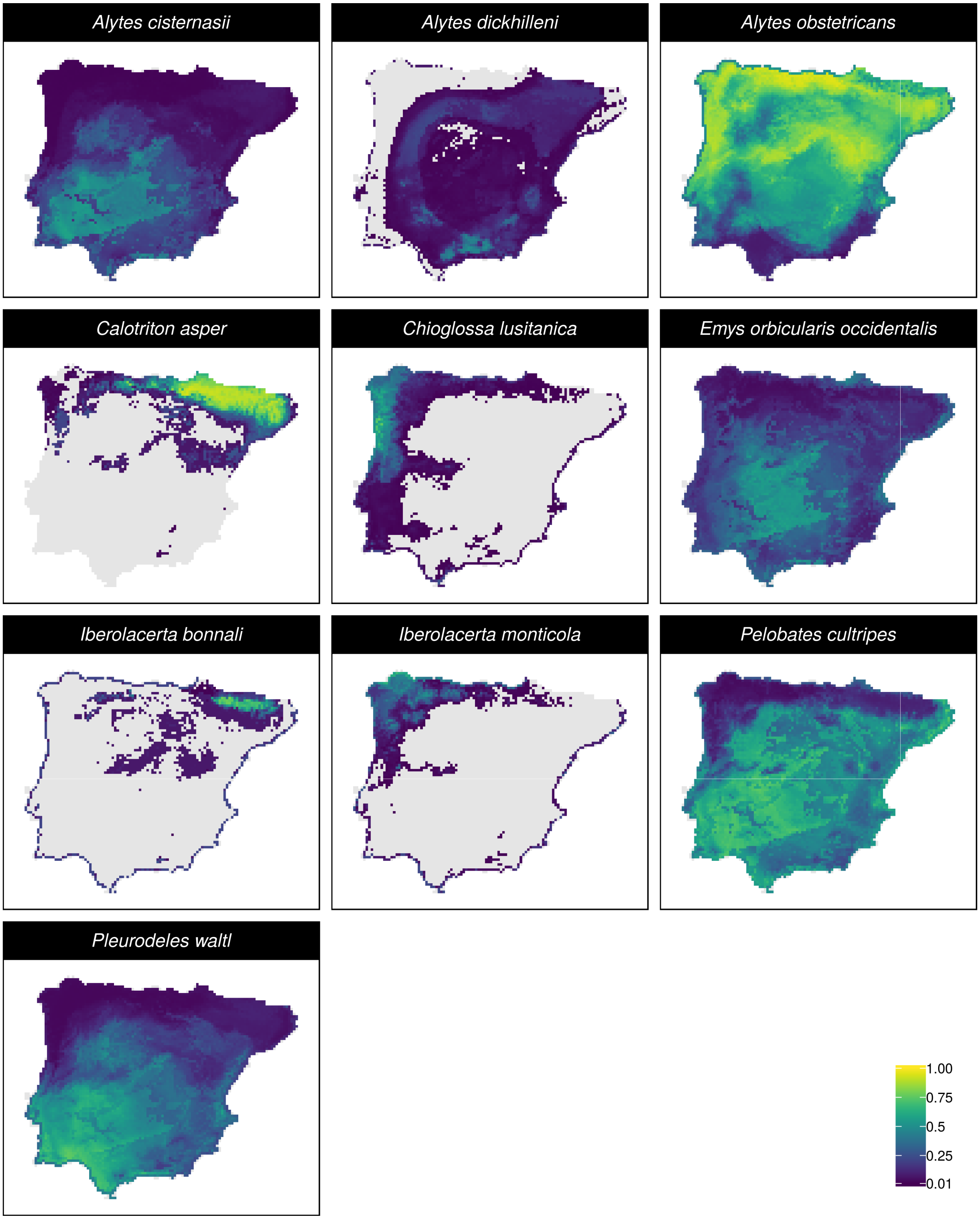

Appendix S23: Maps of historic environmental suitability predicted for the mid-Holocene, Northgrippian (8.326–4.2 ka; 10 × 10 km resolution). To aid with visual interpretation, places with near-zero environmental suitability scores (less than 0.01) are shown in grey.

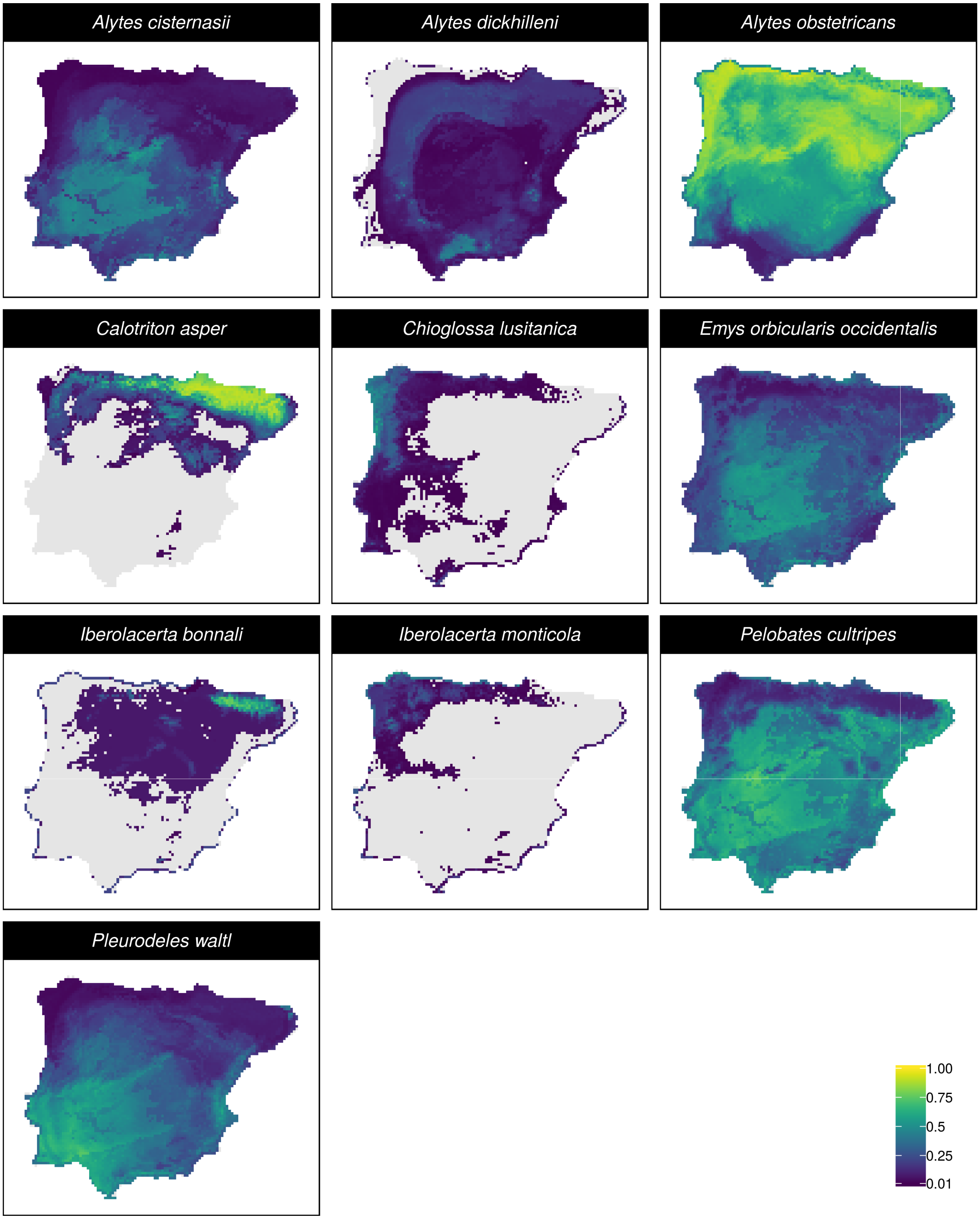

Appendix S24: Maps of historic environmental suitability predicted for the early-Holocene, Greenlandian (11.7–8.326 ka; 10 × 10 km resolution). To aid with visual interpretation, places with near-zero environmental suitability scores (less than 0.01) are shown in grey.

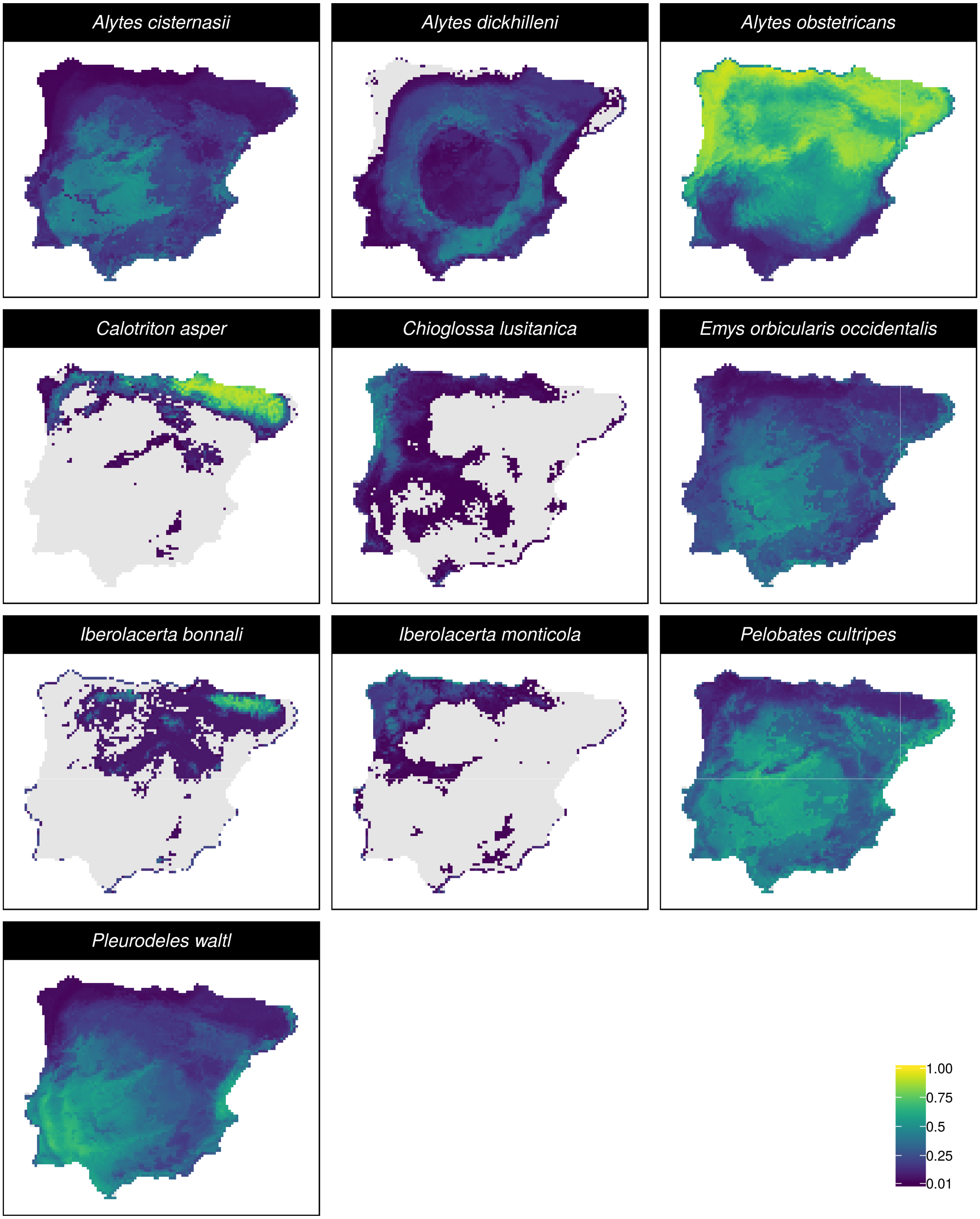

Appendix S25: Maps of historic environmental suitability predicted for the Pleistocene, Younger Dryas Stadial (12.9–11.7 ka; 10 × 10 km resolution). To aid with visual interpretation, places with near-zero environmental suitability scores (less than 0.01) are shown in grey.

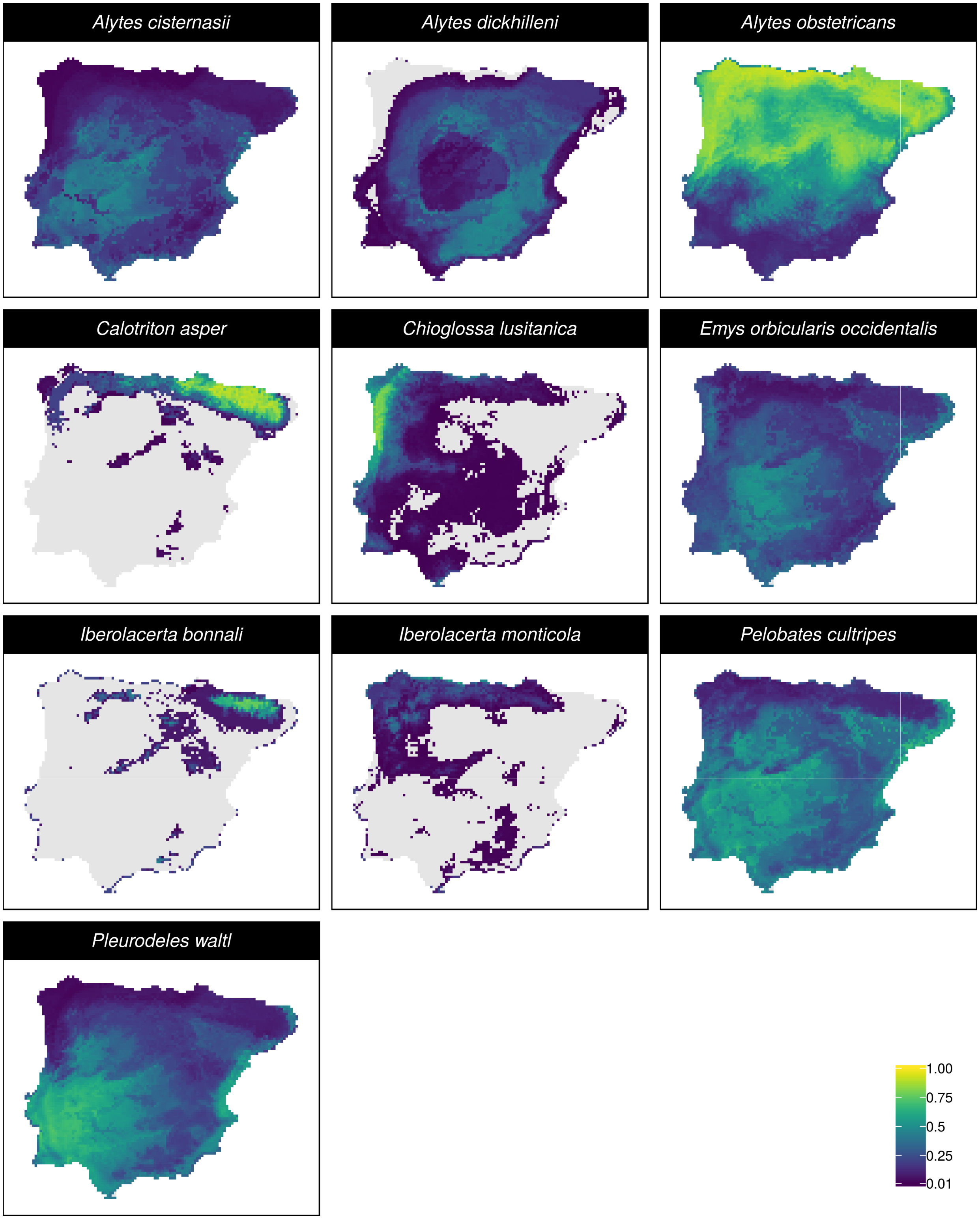

Appendix S26: Maps of historic environmental suitability predicted for the Pleistocene, Bølling-Allerød (14.7–12.9 ka; 10 × 10 km resolution). To aid with visual interpretation, places with near-zero environmental suitability scores (less than 0.01) are shown in grey.

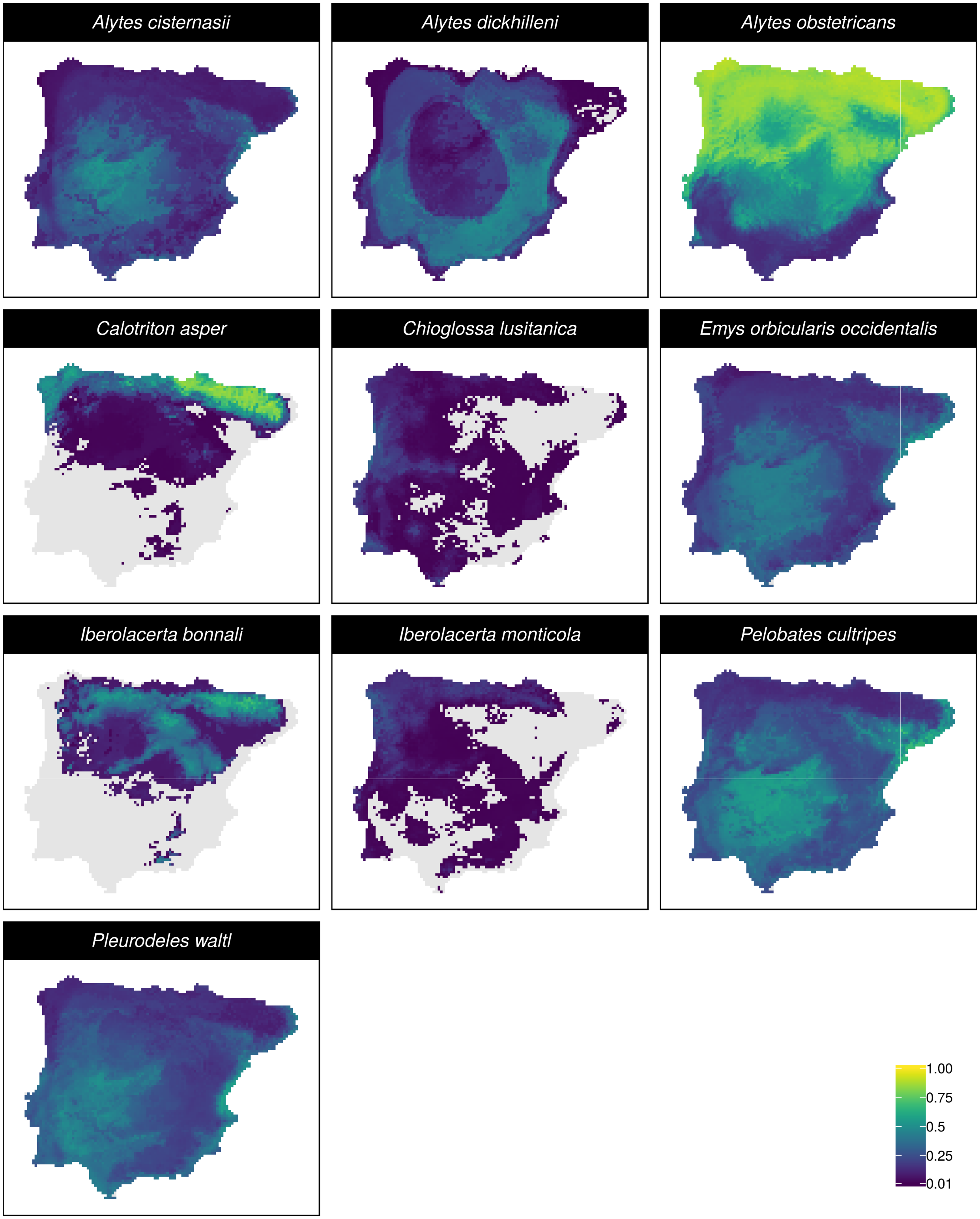

Appendix S27: Maps of historic environmental suitability predicted for the Pleistocene, Heinrich Stadial 1 (17.0–14.7 ka; 10 × 10 km resolution). To aid with visual interpretation, places with near-zero environmental suitability scores (less than 0.01) are shown in grey.

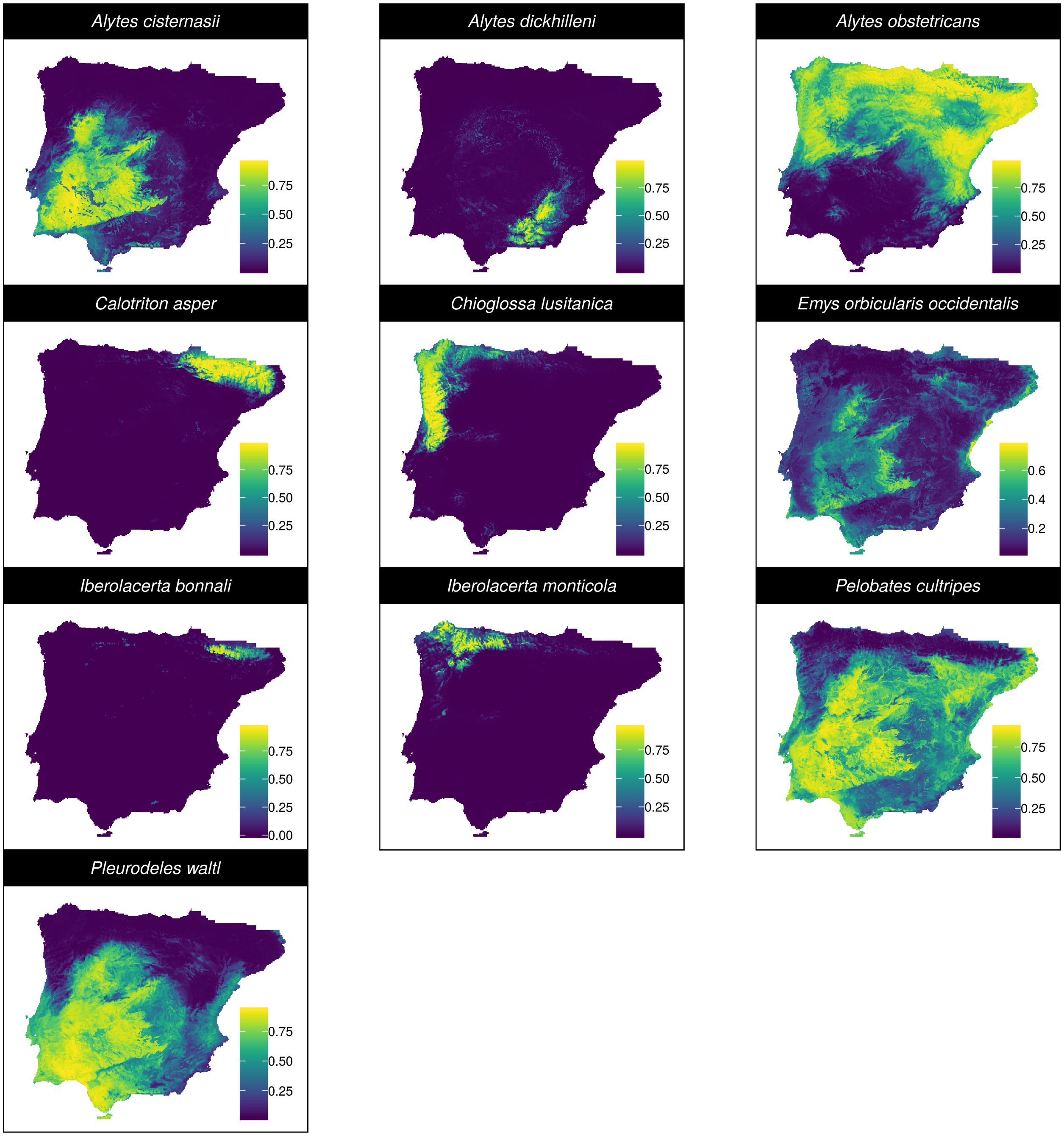

Appendix S28: Maps of contemporary environmental suitability (1 × 1 km resolution). To aid with visual interpretation, places with near-zero environmental suitability scores (less than 0.01) are shown in grey.

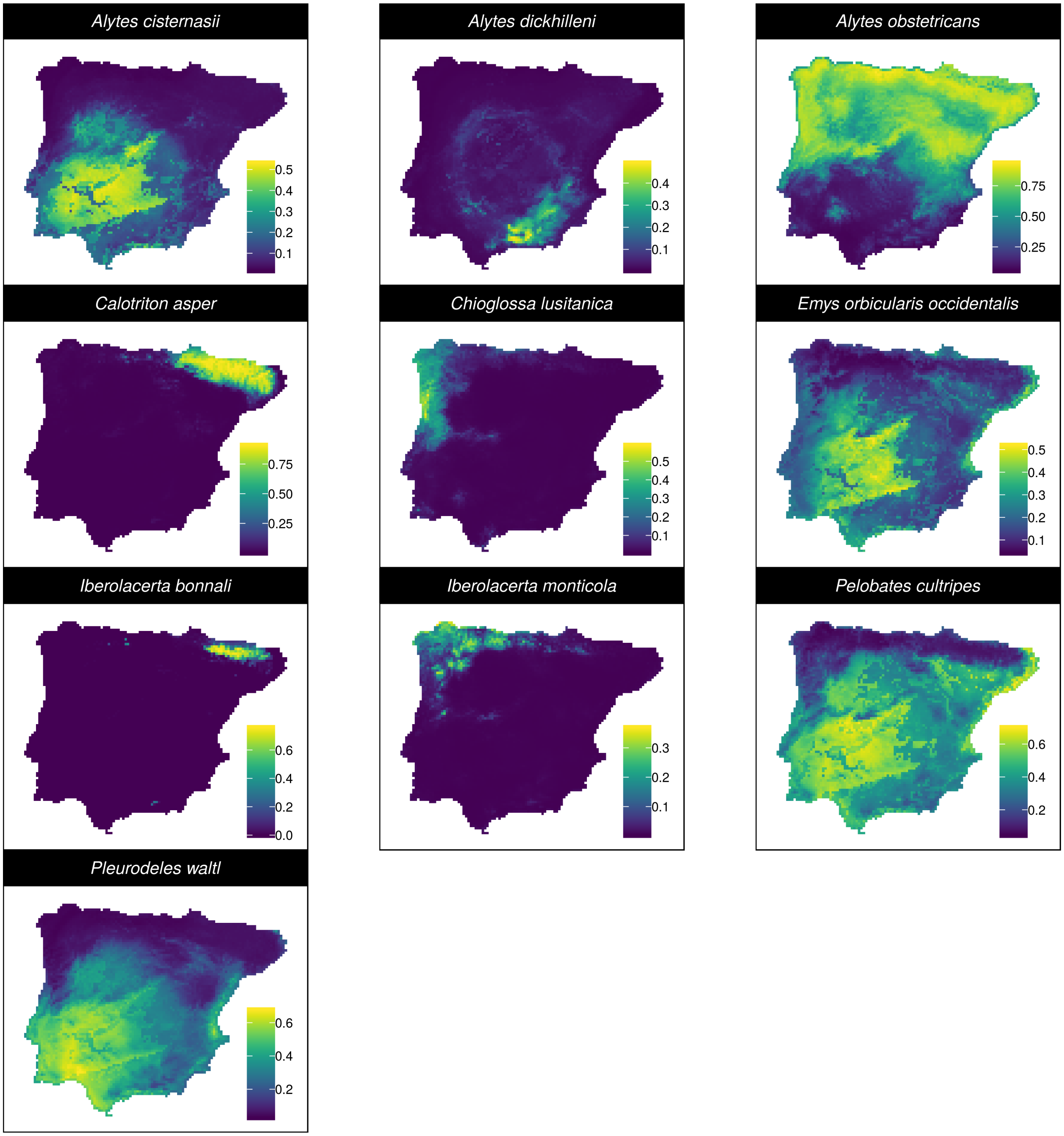

Appendix S29: Maps of long-term environmental suitability (10 × 10 km resolution).

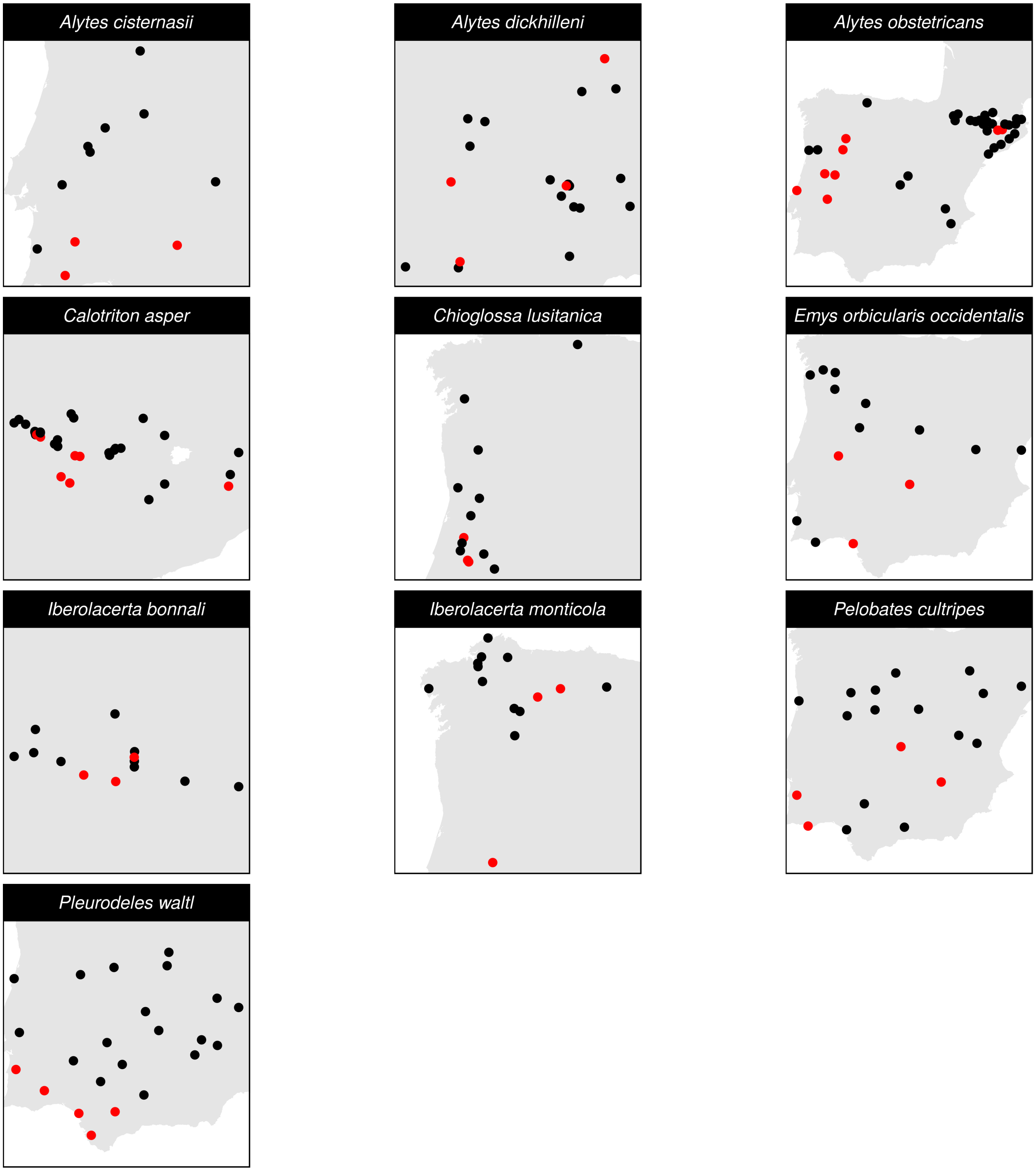

Appendix S30: Sites with high allelic richness for each species. Red points denote sites with high allelic richness (greater than 80th percentile), and black points denote the remaining sites.

Appendix S31: The environmental diversity formulation of the reserve selection problem aims to prioritise a set of sites that contains a diverse range of environmental conditions (Faith & Walker 1996). Specifically, it finds the subset of sites that contain the most representative set of environmental conditions among all candidate sites, subject to a limit on the number of sites that can be selected. It uses a dissimilarity (or distance) matrix to characterize environmental differences between each pair of sites. As a consequence, it can be adapted to generate prioritisations that spread conservation effort evenly across geographic space by using a geographic distance matrix instead of an environmental dissimilarity matrix. Similarly, it can also be used to generate prioritisations that spread conservation effort evenly across dispersal barriers using a resistance distance matrix. Furthermore, it can also be used to generate prioritisations that secure a representative sample of the genetic diversity among sites using a pairwise genetic distance matrix.

This formulation of the reserve selection problem can be defined mathematically. Let J denote a set of candidate sites for conservation (indexed by both i and j). Also, let n denote the maximum number of sites that can be selected for conservation in prioritisation. Furthermore, let D_{ij} denote the dissimilarity (or distance) between each pair of sites $i \in J$ and sites $j \in J$. The decision variables for this problem are the binary X_j and Y_{ij} variables. Here, the X_j variable indicates which sites are (1) selected for conservation or (0) not. The Y_{ij} variables indicates the closest selected site to each site $j \in J$.

$$
\begin{aligned}
\min \sum_{i \in J} \sum_{j \in J} D_{ij} Y_{ij} & \\
\text{subject to} \sum_{j \in J} X_j = n & \\
Y_{ij} \leq X_j & \qquad \forall i \in J, j \in J \\
\sum_{j \in J} Y_{ij} = 1 & \qquad \forall i \in J \\
X_j \in \{0, 1\} & \qquad \forall j \in J \\
Y_{ij} \in \{0, 1\} & \qquad \forall i \in J, j \in J
\end{aligned}
$$

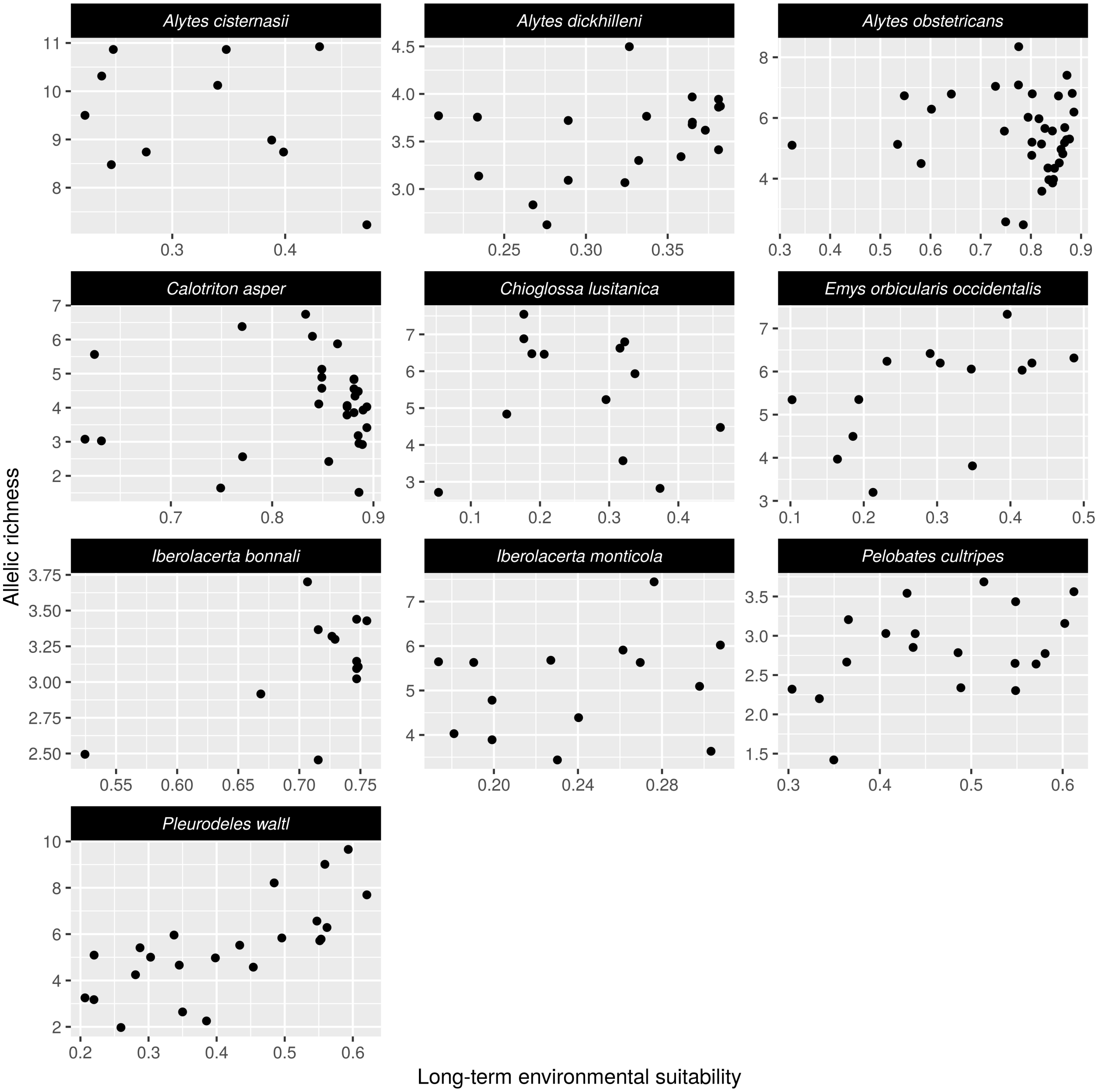

Appendix S32: The relationship between the allelic richness measured at a site and the long-term environmental suitability at the site. Each panel corresponds to a different species, and points correspond to sites.

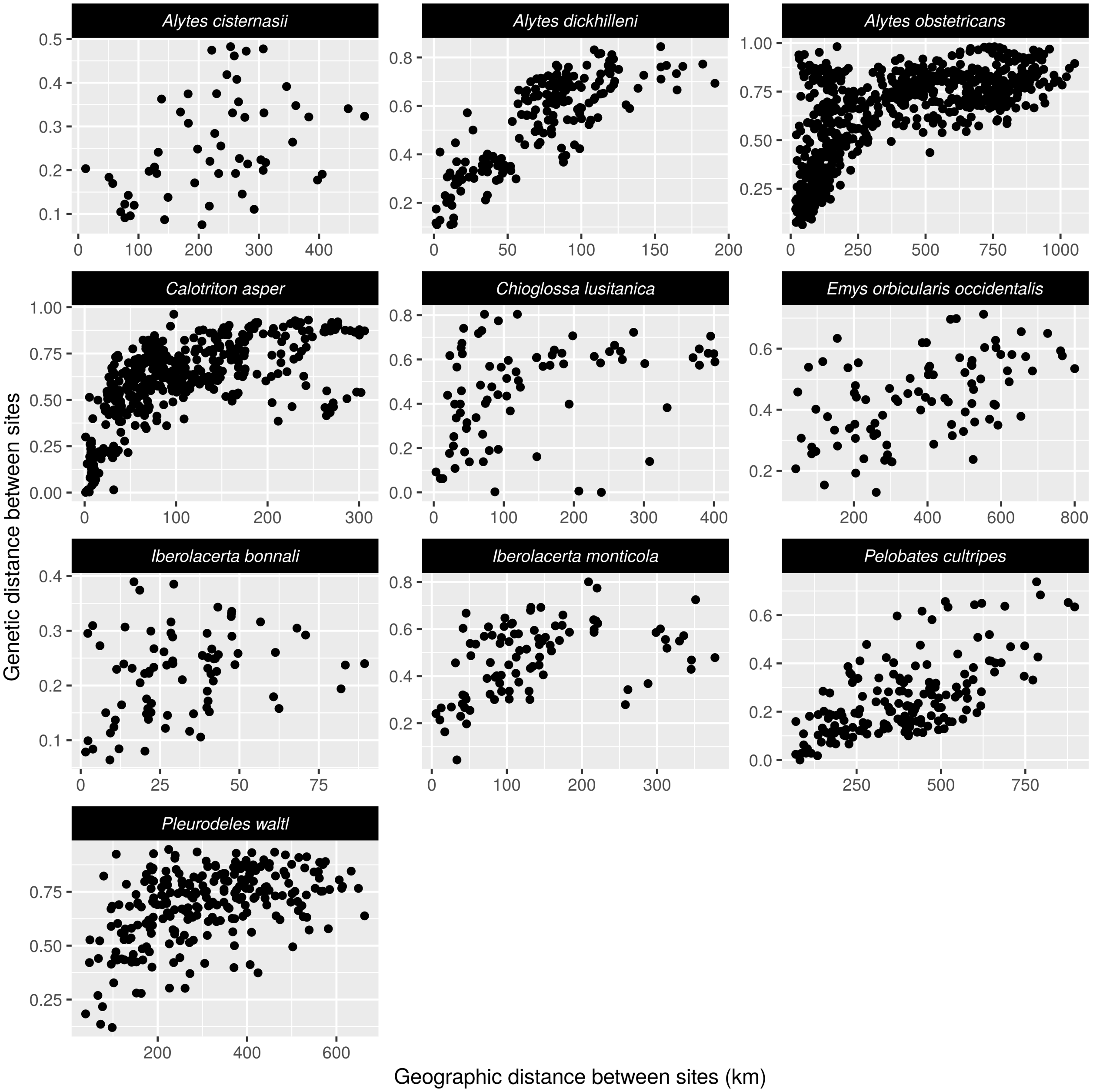

Appendix S33: The relationship between the genetic distances and geographic distance between sites. Genetic distances were computed as pairwise Jost's D statistics. Geographic distances were computed as the Euclidean distance between site coordinates in a projected coordinate system. Each panel corresponds to a different species, and points correspond to sites.

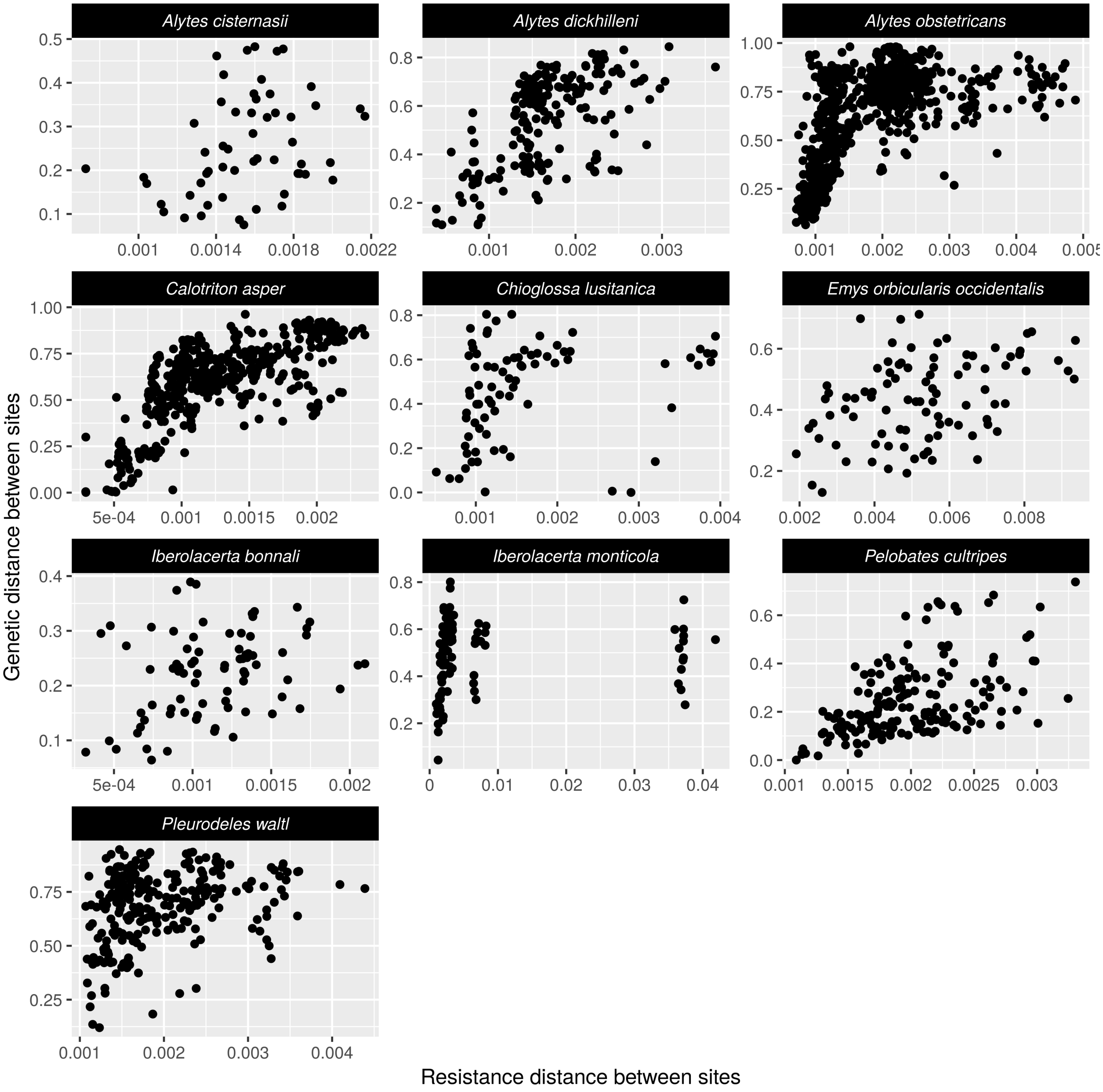

Appendix S34: The relationship between the genetic distances and resistance distances between sites. Genetic distances were computed as pairwise Jost's D statistics. Resistance distances were computed as commute distances between sites using inverse environmental suitability to parametrize landscape traversal cost. See Figure S33 caption for conventions.

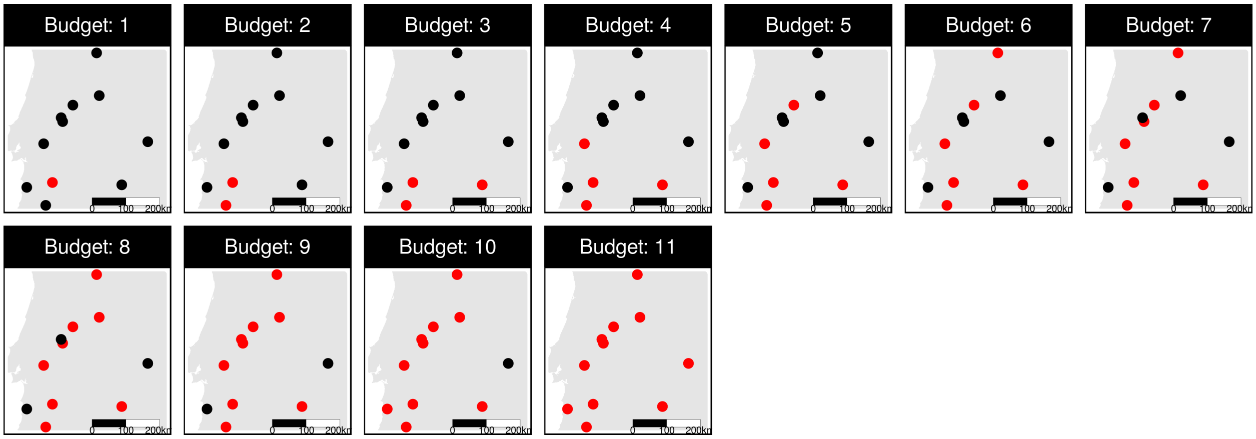

Appendix S35: Site-based prioritisations generated for *Alytes cisternasii* using allelic richness measures. Panels correspond to different budgets, red points denote selected sites, and black points denote unselected sites.

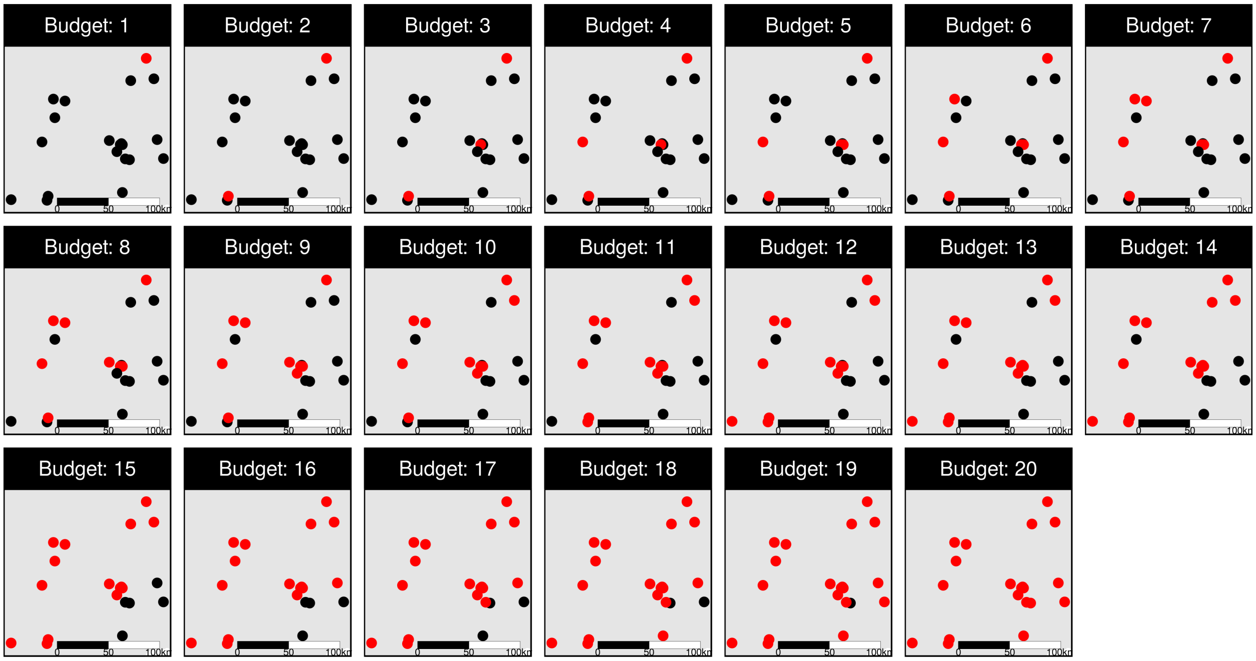

Appendix S36: Site-based prioritisations generated for *Alytes dickhilleni* using allelic richness measures. Panels correspond to different budgets, red points denote selected sites, and black points denote unselected sites.

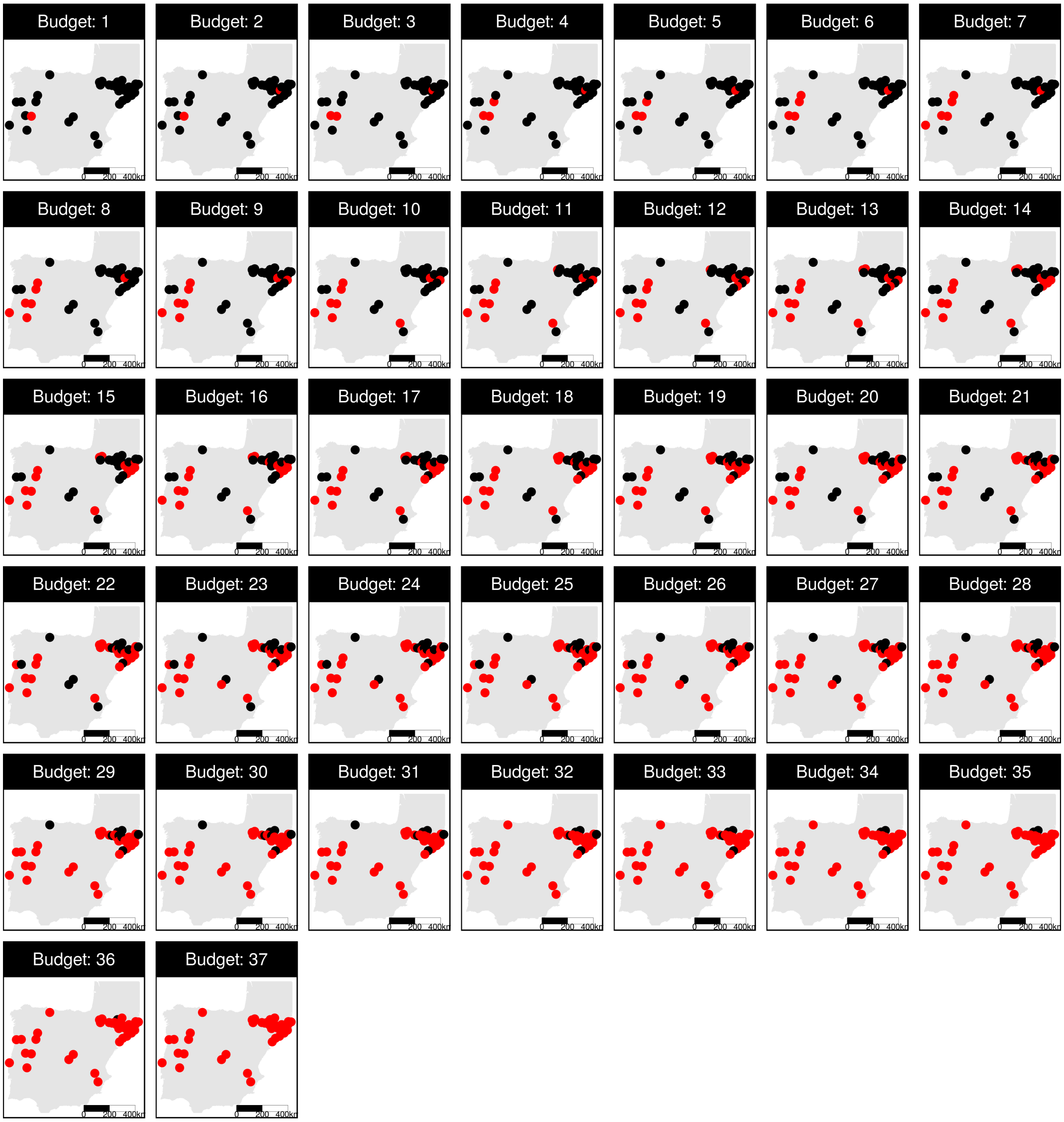

Appendix S37: Site-based prioritisations generated for *Alytes obstetricans* using allelic richness measures. Panels correspond to different budgets, red points denote selected sites, and black points denote unselected sites.

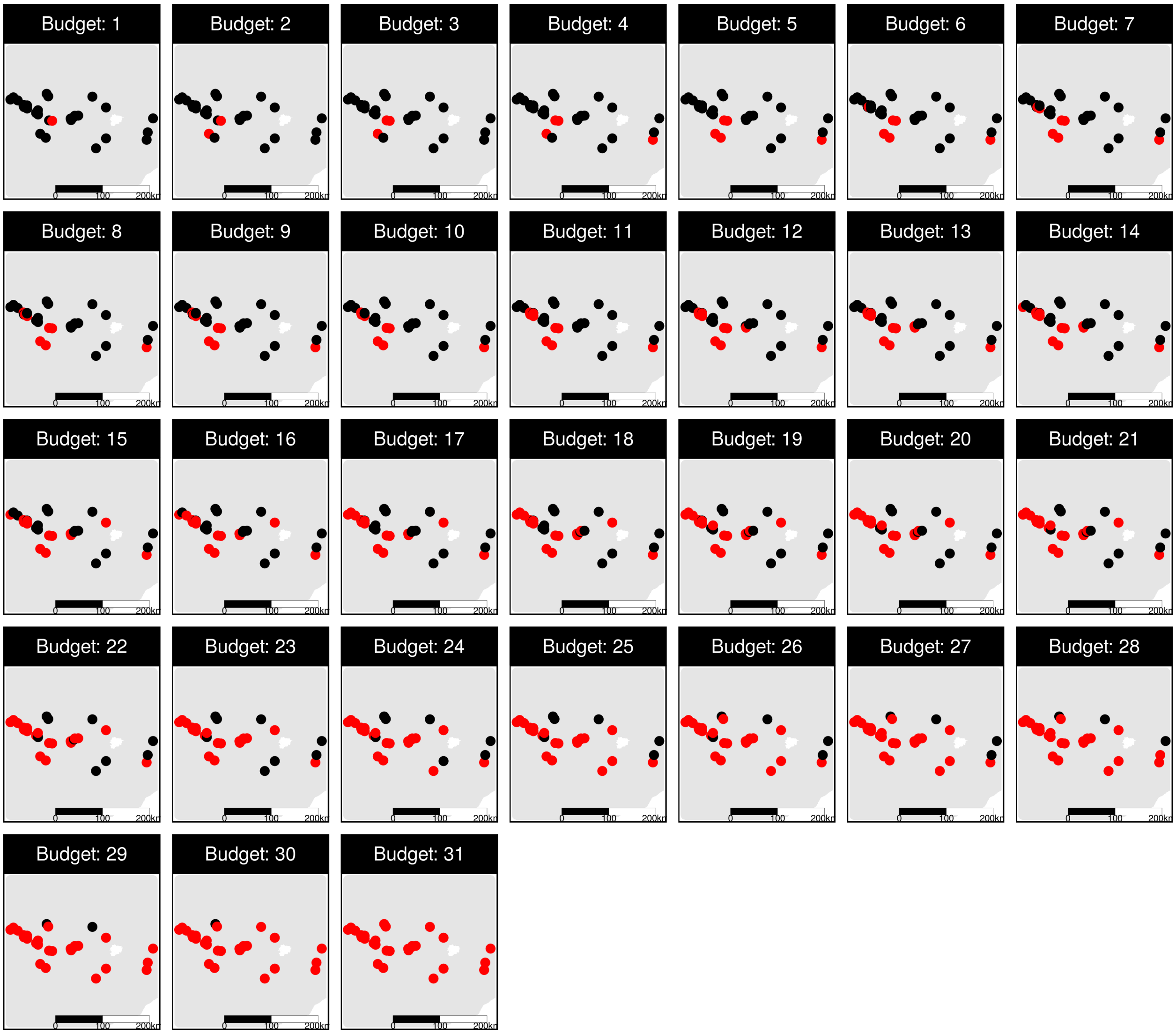

Appendix S38: Site-based prioritisations generated for *Calotriton asper* using allelic richness measures. Panels correspond to different budgets, red points denote selected sites, and black points denote unselected sites.

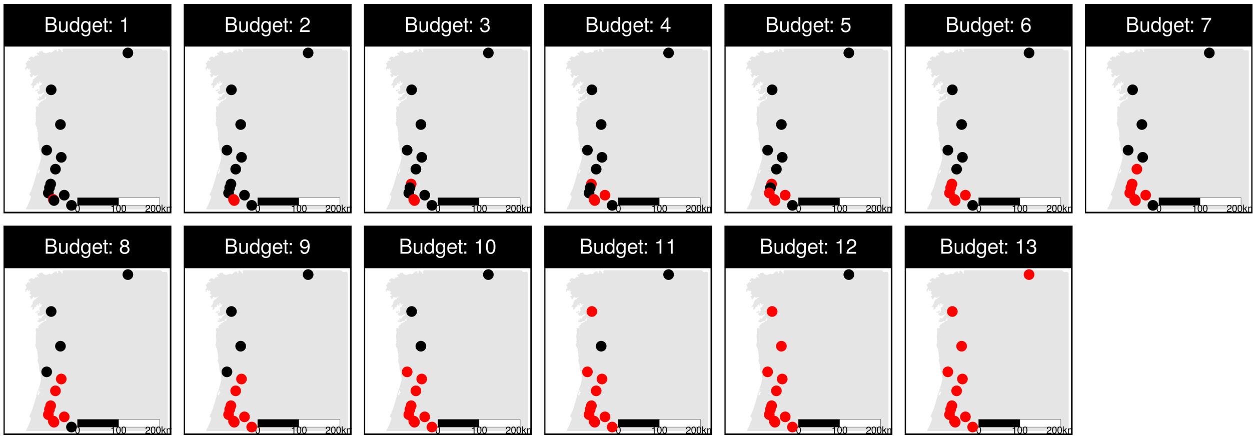

Appendix S39: Site-based prioritisations generated for *Chioglossa lusitanica* using allelic richness measures. Panels correspond to different budgets, red points denote selected sites, and black points denote unselected sites.

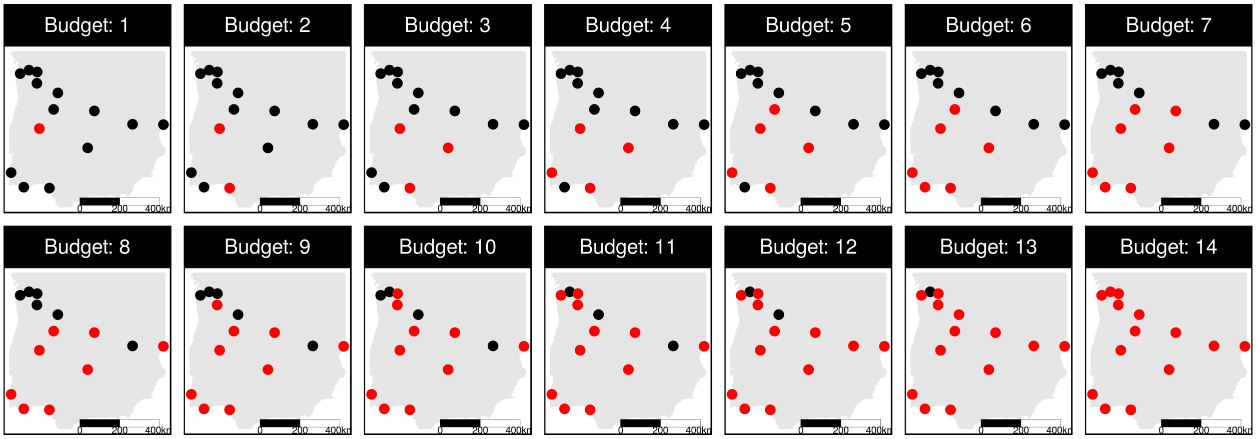

Appendix S40: Site-based prioritisations generated for *Emys orbicularis occidentalis* using allelic richness measures. Panels correspond to different budgets, red points denote selected sites, and black points denote unselected sites.

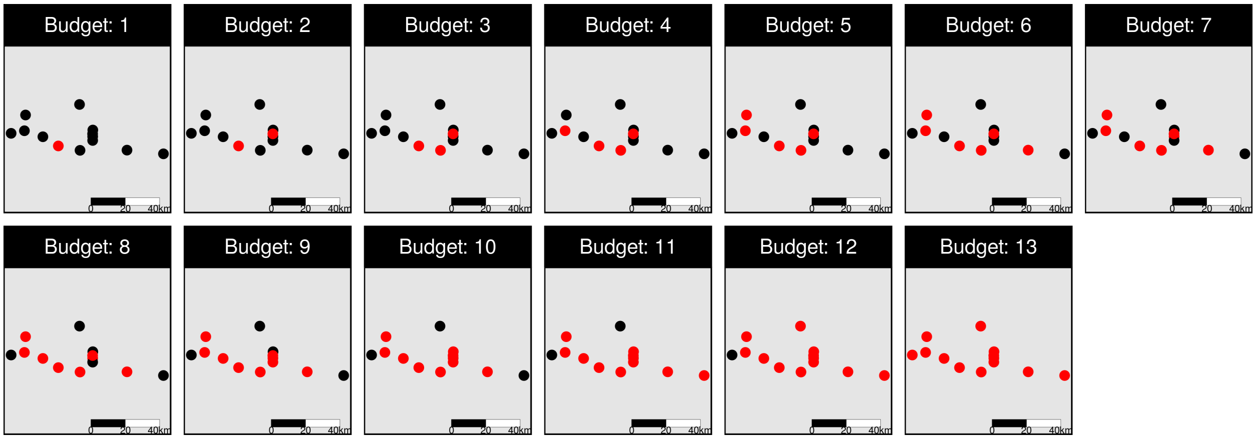

Appendix S41: Site-based prioritisations generated for *Iberolacerta bonnali* using allelic richness measures. Panels correspond to different budgets, red points denote selected sites, and black points denote unselected sites.

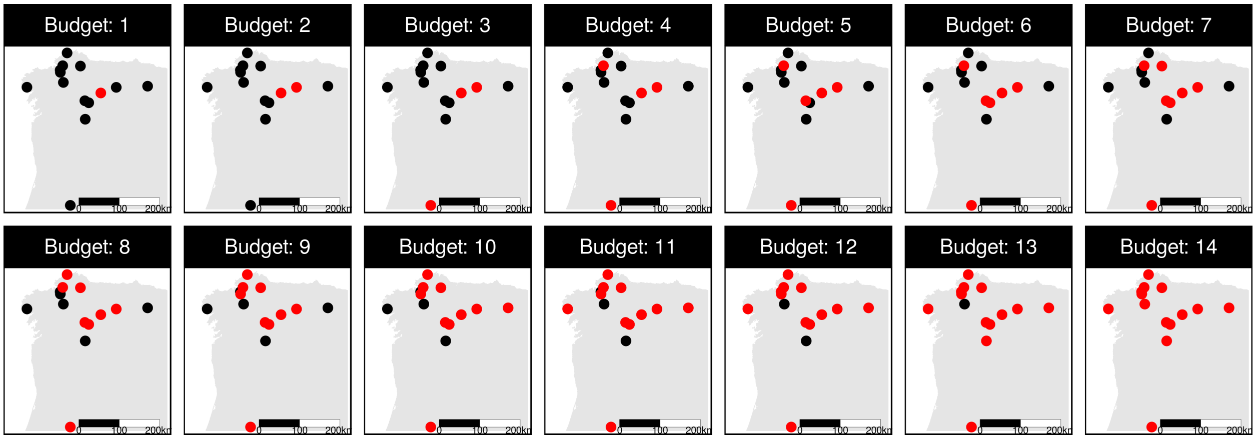

Appendix S42: Site-based prioritisations generated for *Iberolacerta monticola* using allelic richness measures. Panels correspond to different budgets, red points denote selected sites, and black points denote unselected sites.

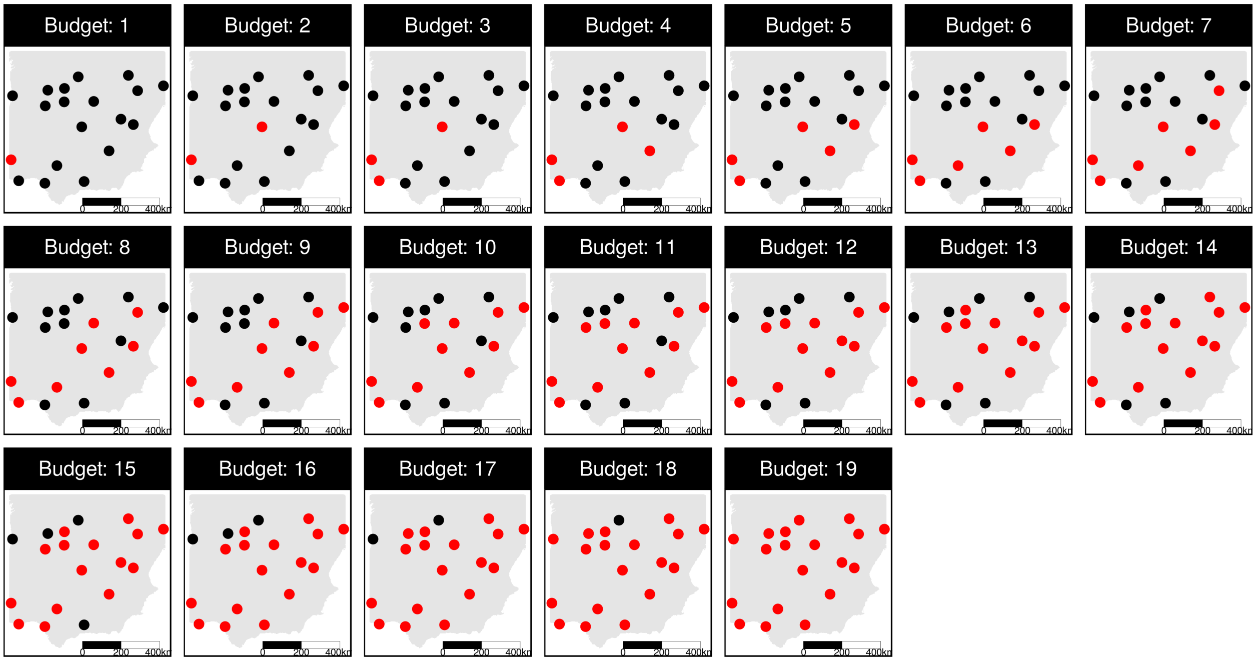

Appendix S43: Site-based prioritisations generated for *Pelobates cultripes* using allelic richness measures. Panels correspond to different budgets, red points denote selected sites, and black points denote unselected sites.

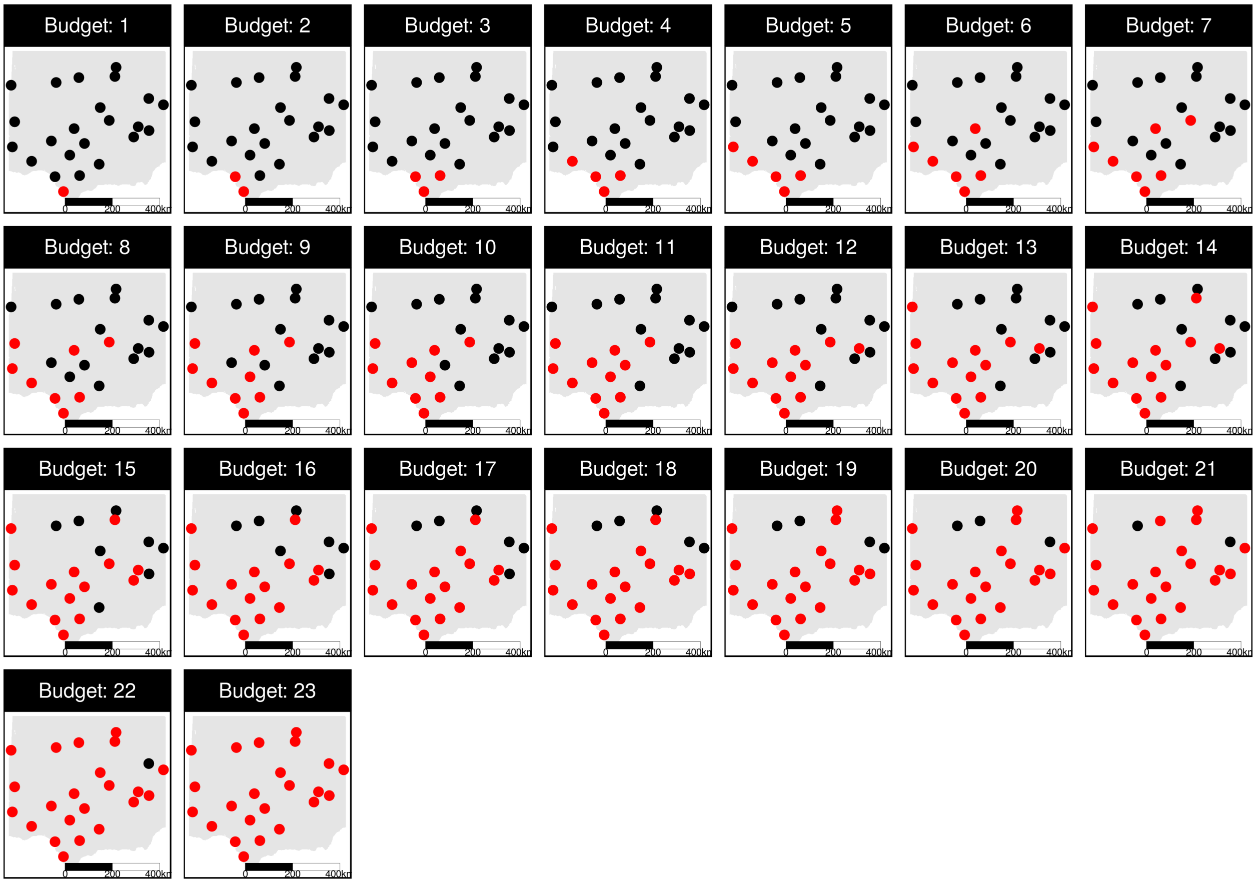

Appendix S44: Site-based prioritisations generated for *Pleurodeles waltl* using allelic richness measures. Panels correspond to different budgets, red points denote selected sites, and black points denote unselected sites.

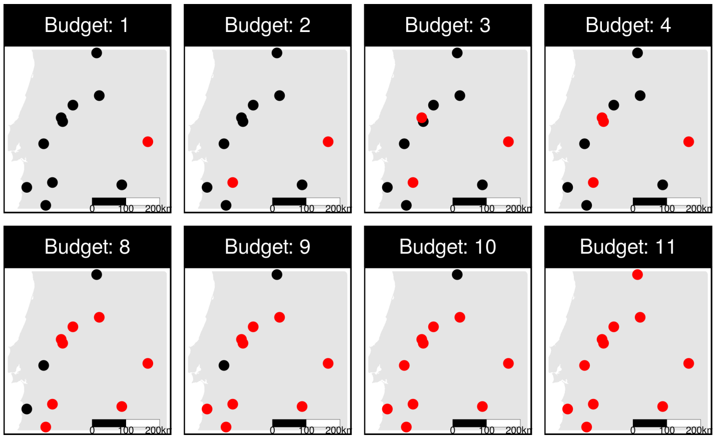

Appendix S45: Site-based prioritisations generated for *Alytes cisternasii* using long-term environmental suitability. Panels correspond to different budgets, red points denote selected sites, and black points denote unselected sites.

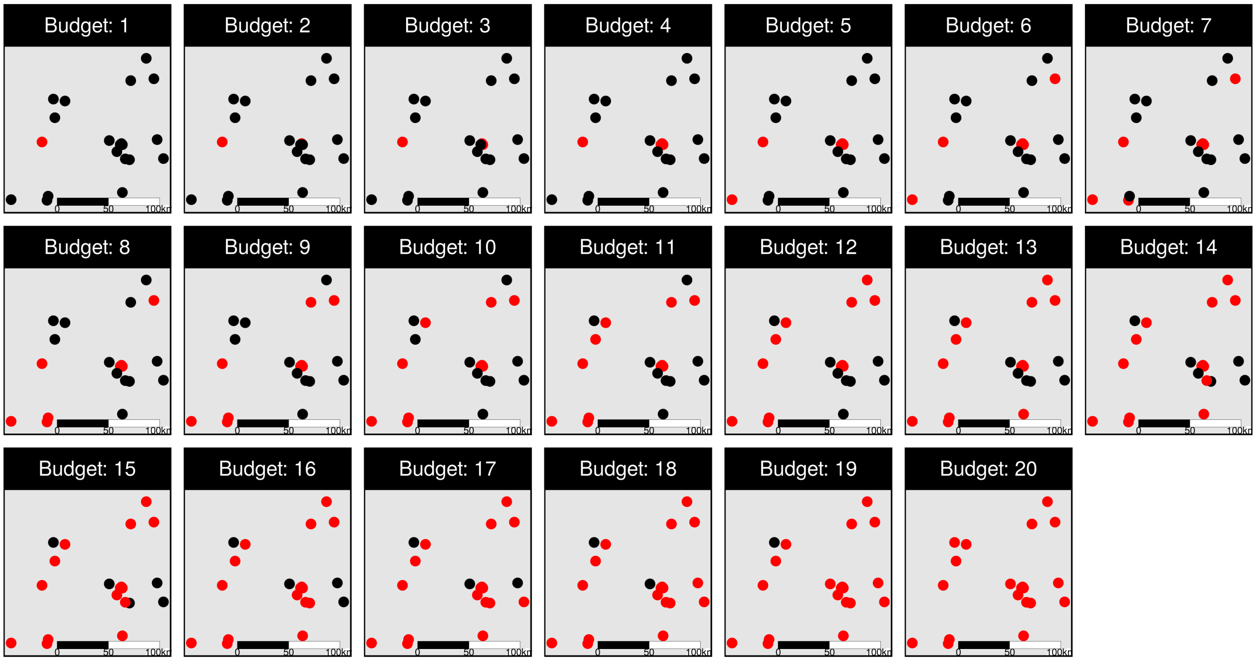

Appendix S46: Site-based prioritisations generated for *Alytes dickhilleni* using long-term environmental suitability. Panels correspond to different budgets, red points denote selected sites, and black points denote unselected sites.

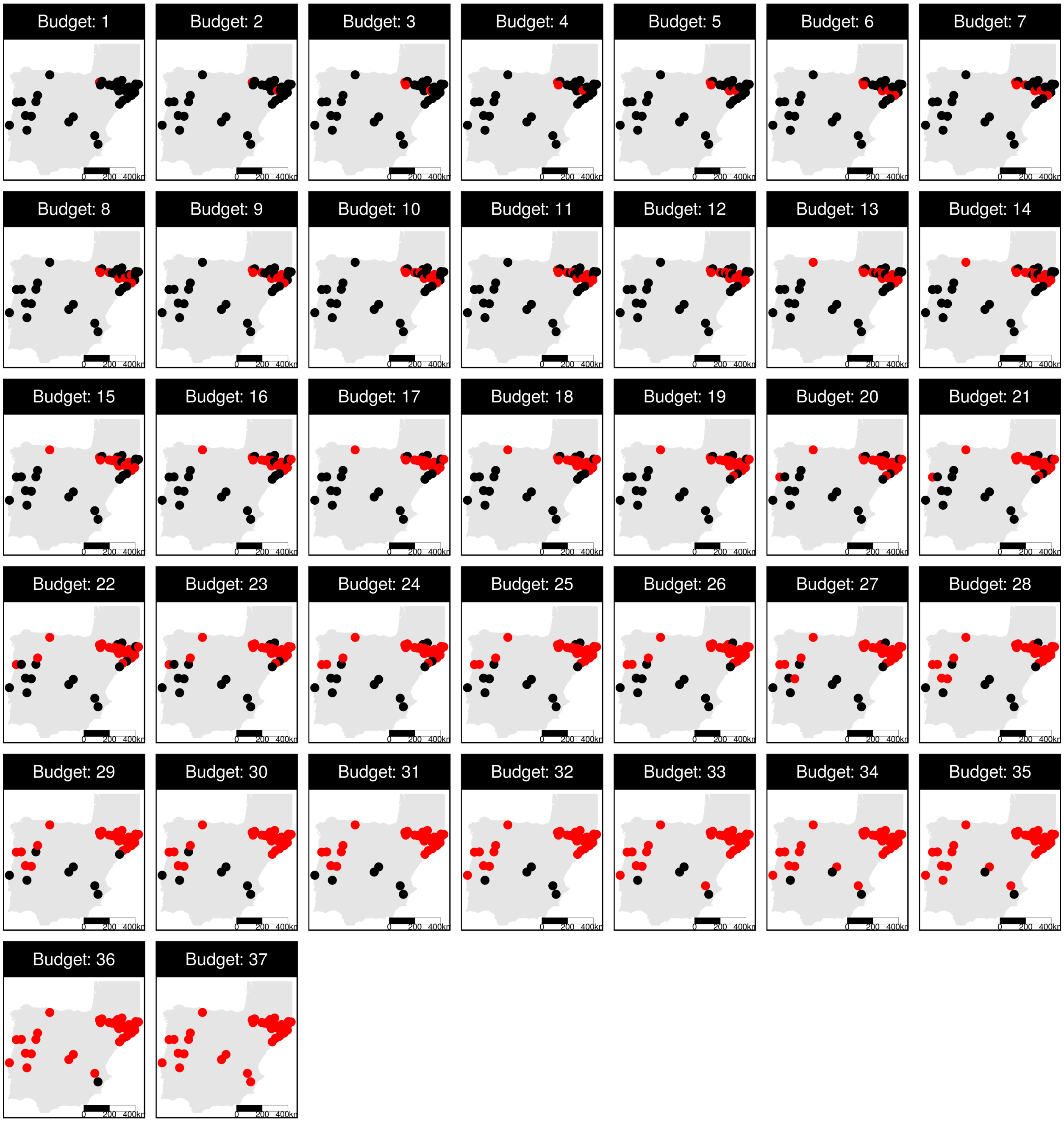

Appendix S47: Site-based prioritisations generated for *Alytes obstetricans* using long-term environmental suitability. Panels correspond to different budgets, red points denote selected sites, and black points denote unselected sites.

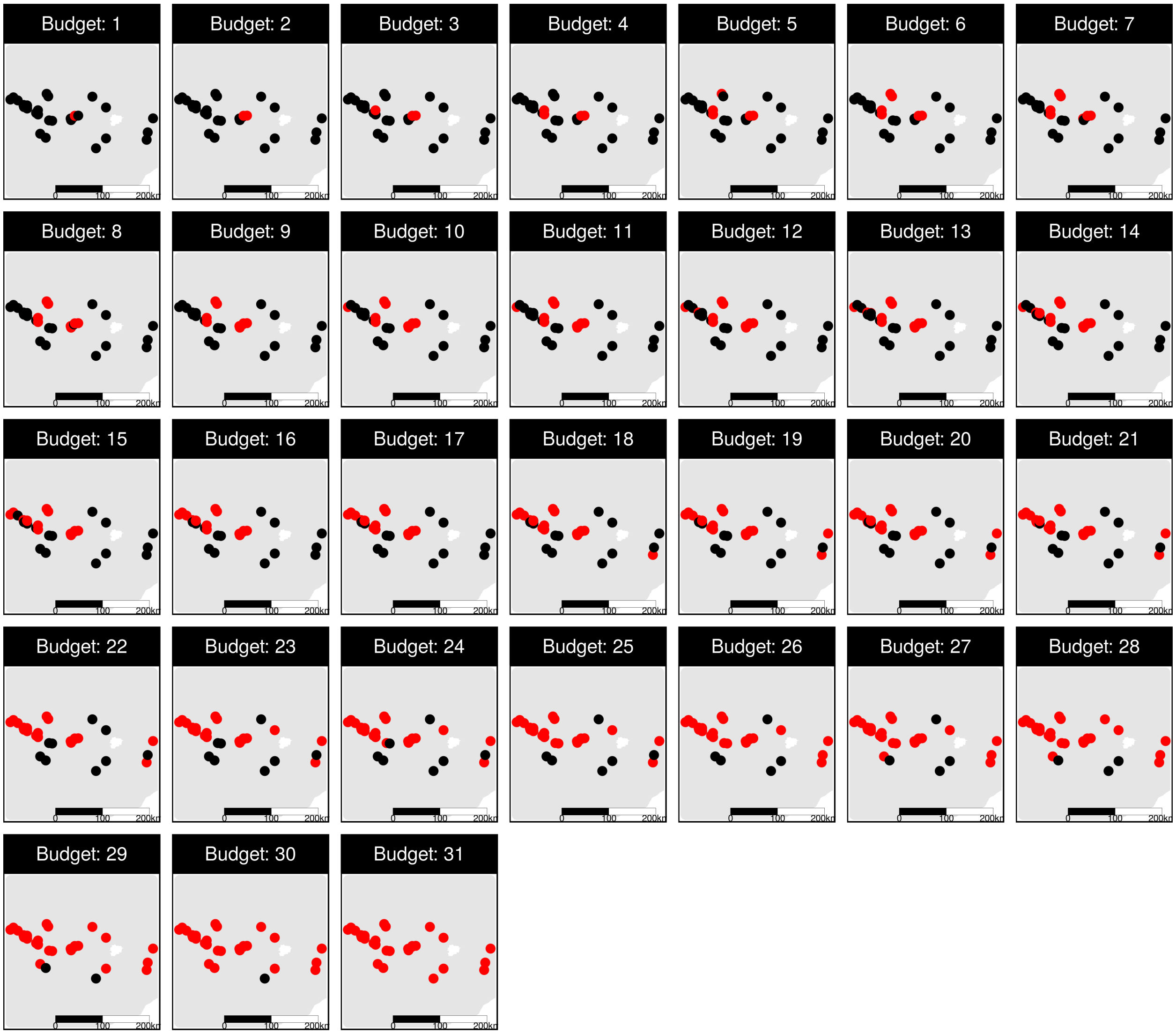

Appendix S48: Site-based prioritisations generated for *Calotriton asper* using long-term environmental suitability. Panels correspond to different budgets, red points denote selected sites, and black points denote unselected sites.

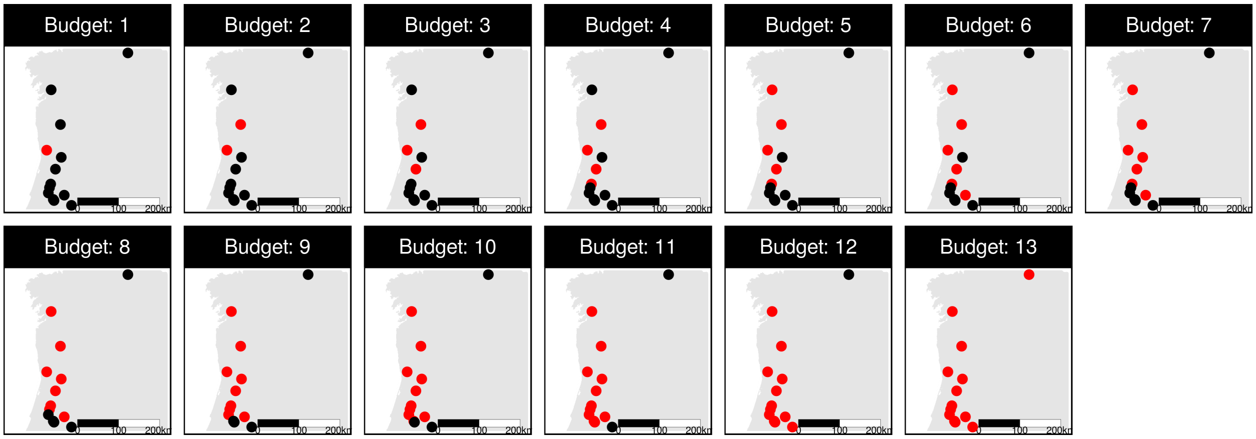

Appendix S49: Site-based prioritisations generated for *Chioglossa lusitanica* using long-term environmental suitability. Panels correspond to different budgets, red points denote selected sites, and black points denote unselected sites.

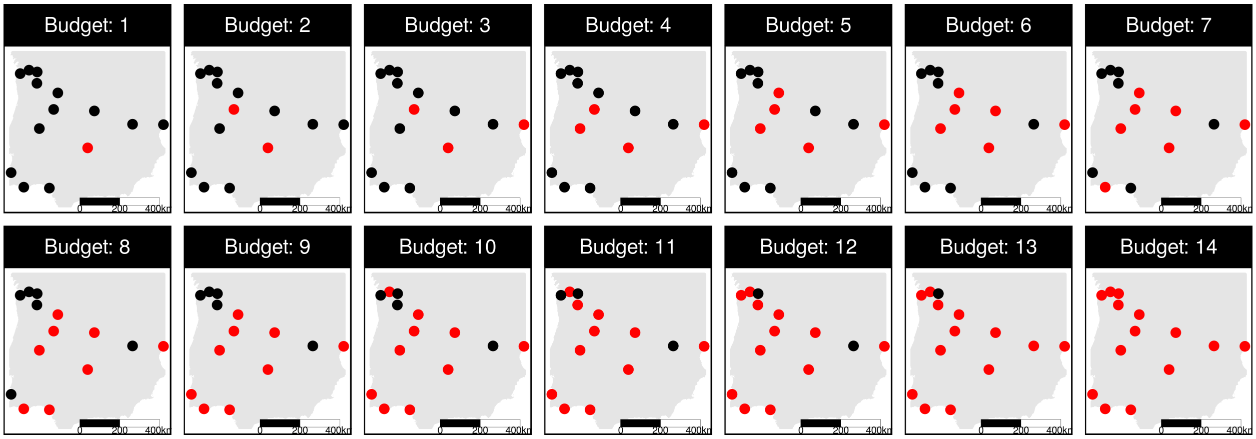

Appendix S50: Site-based prioritisations generated for *Emys orbicularis occidentalis* using long-term environmental suitability. Panels correspond to different budgets, red points denote selected sites, and black points denote unselected sites.

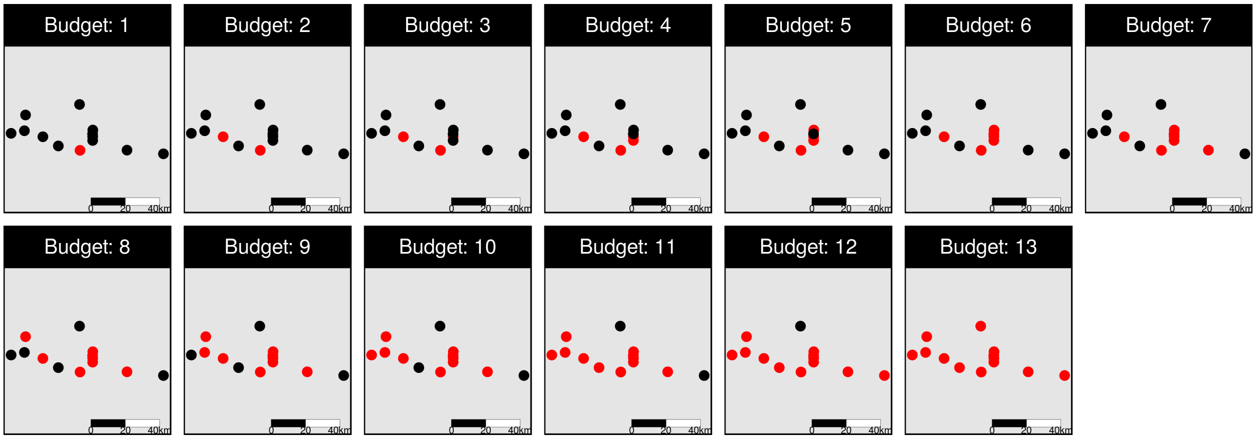

Appendix S51: Site-based prioritisations generated for *Iberolacerta bonnali* using long-term environmental suitability. Panels correspond to different budgets, red points denote selected sites, and black points denote unselected sites.

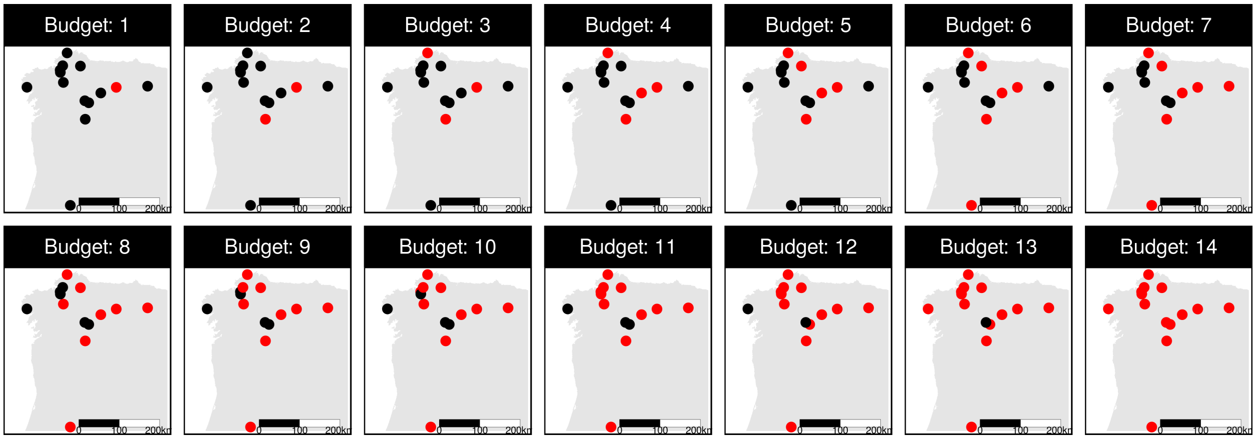

Appendix S52: Site-based prioritisations generated for *Iberolacerta monticola* using long-term environmental suitability. Panels correspond to different budgets, red points denote selected sites, and black points denote unselected sites.

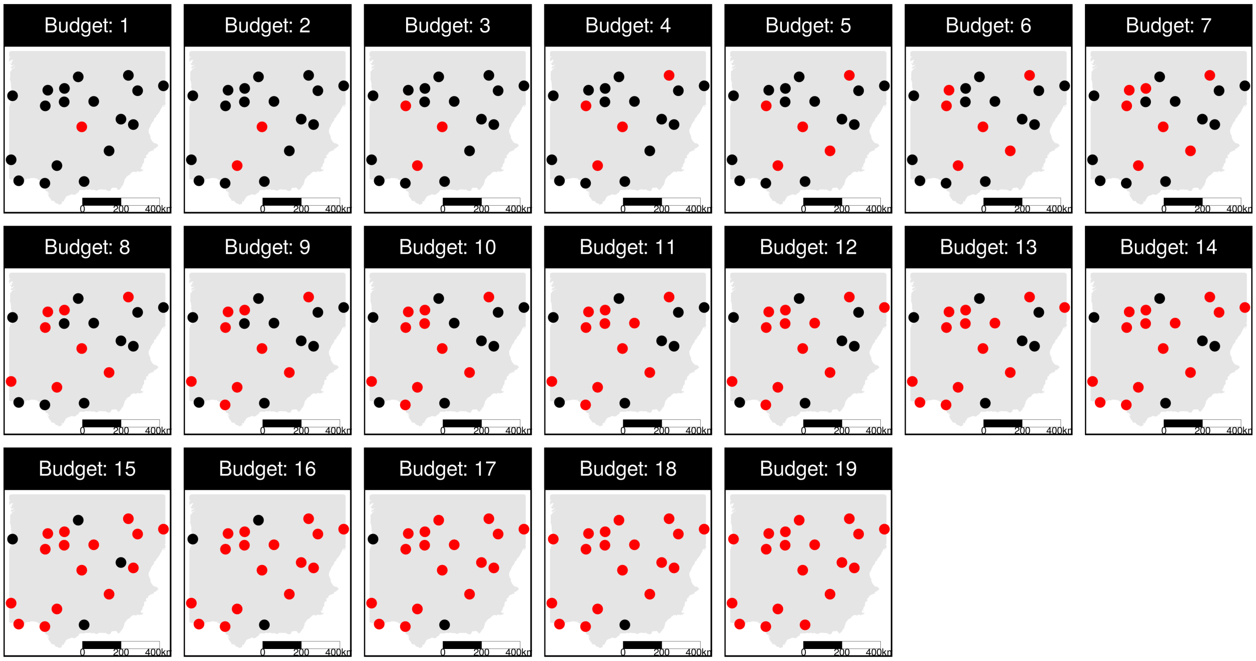

Appendix S53: Site-based prioritisations generated for *Pelobates cultripes* using long-term environmental suitability. Panels correspond to different budgets, red points denote selected sites, and black points denote unselected sites.

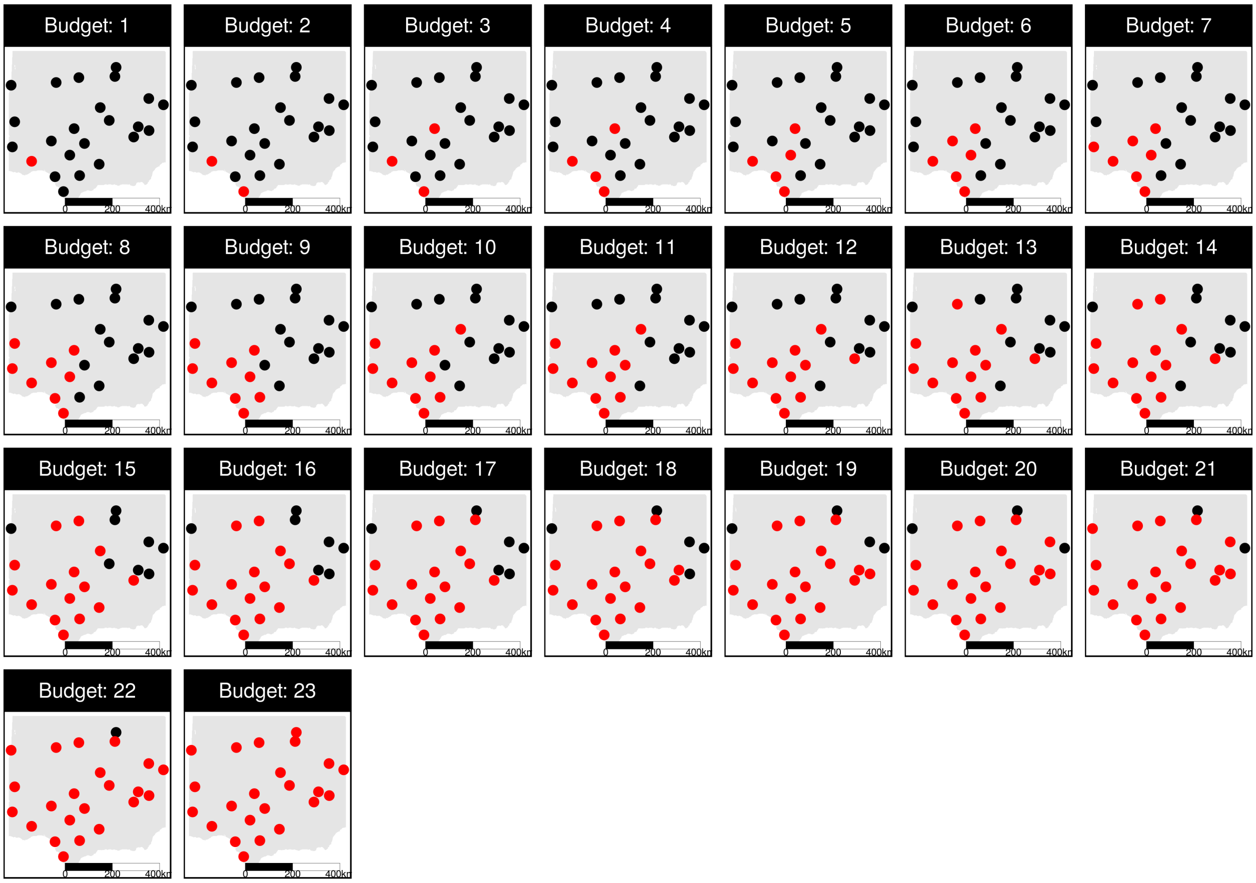

Appendix S54: Site-based prioritisations generated for *Pleurodeles waltl* using long-term environmental suitability. Panels correspond to different budgets, red points denote selected sites, and black points denote unselected sites.

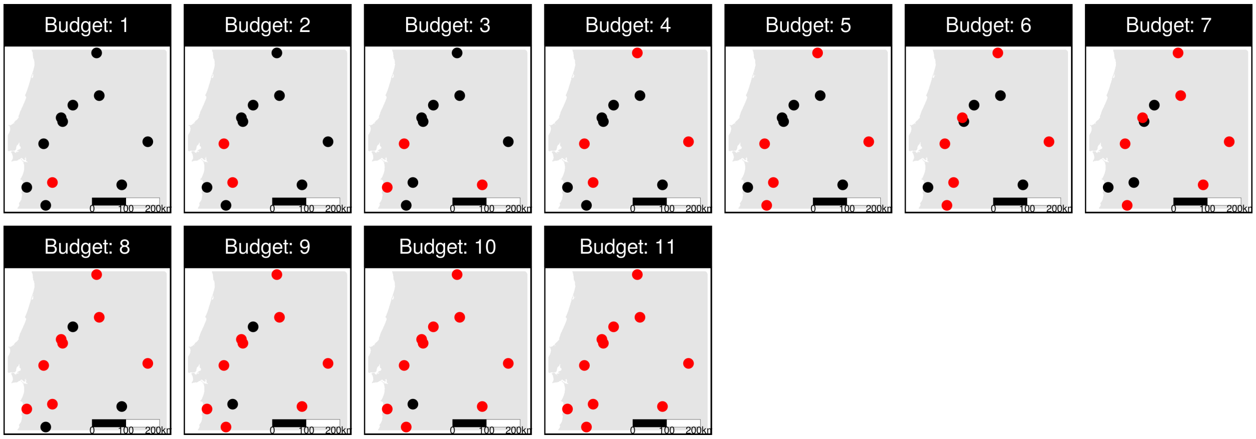

Appendix S55: Site-based prioritisations generated for *Alytes cisternasii* using genetic distances. Panels correspond to different budgets, red points denote selected sites, and black points denote unselected sites.

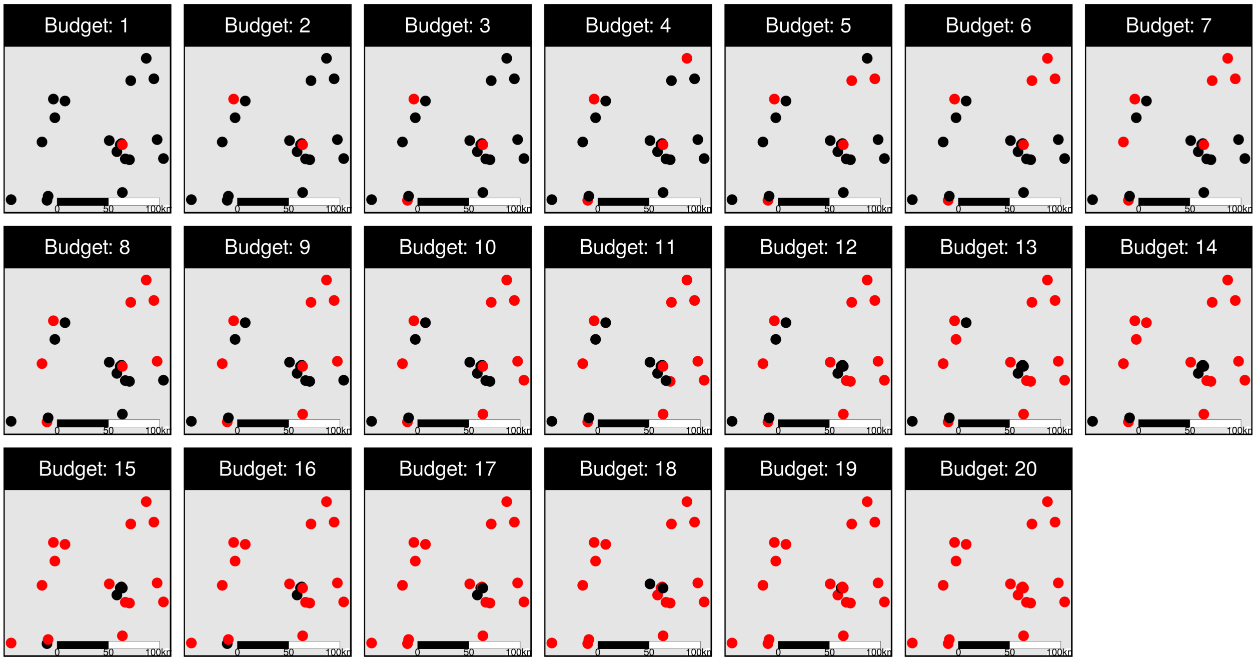

Appendix S56: Site-based prioritisations generated for *Alytes dickhilleni* using genetic distances. Panels correspond to different budgets, red points denote selected sites, and black points denote unselected sites.

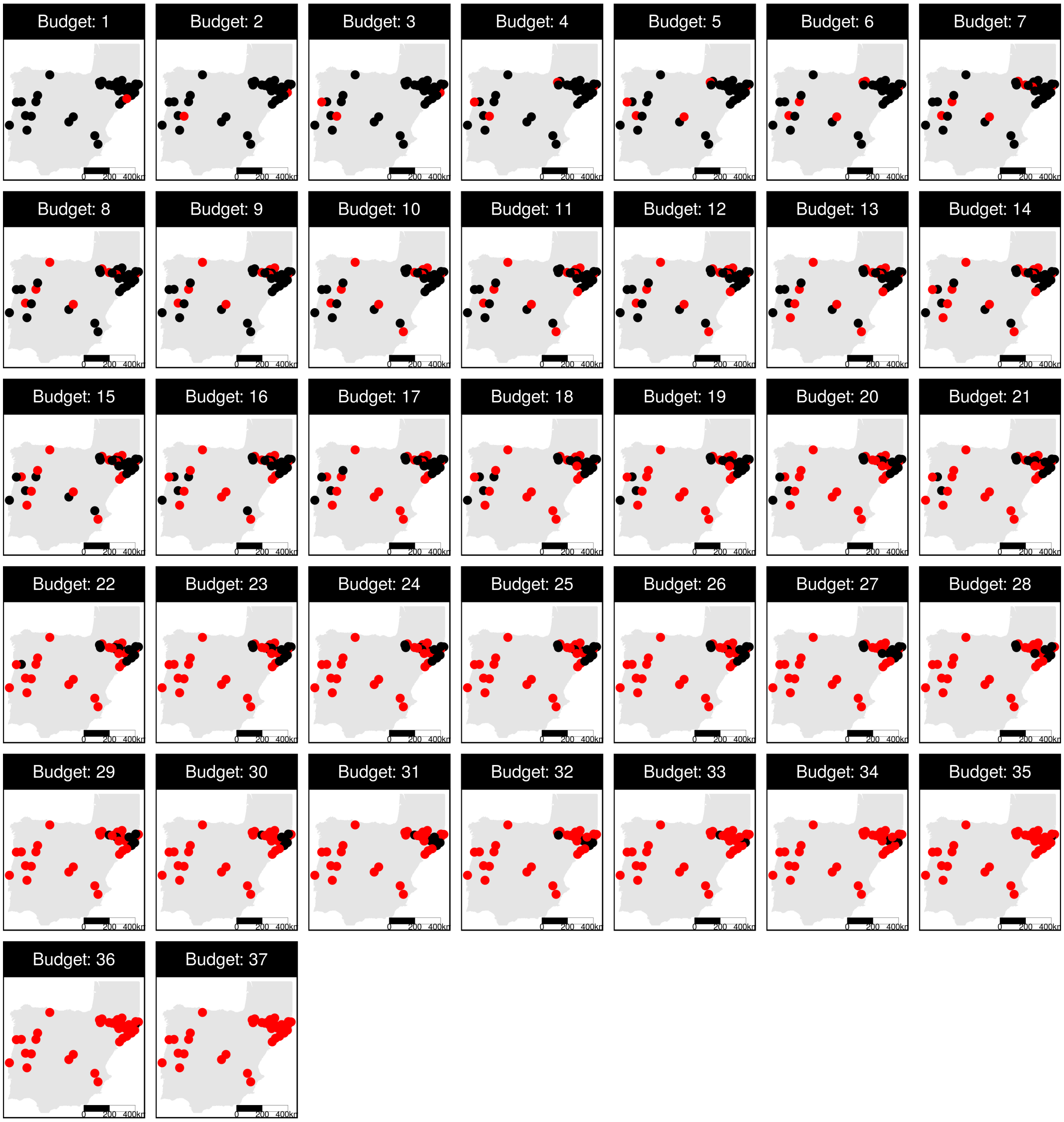

Appendix S57: Site-based prioritisations generated for *Alytes obstetricans* using genetic distances. Panels correspond to different budgets, red points denote selected sites, and black points denote unselected sites.

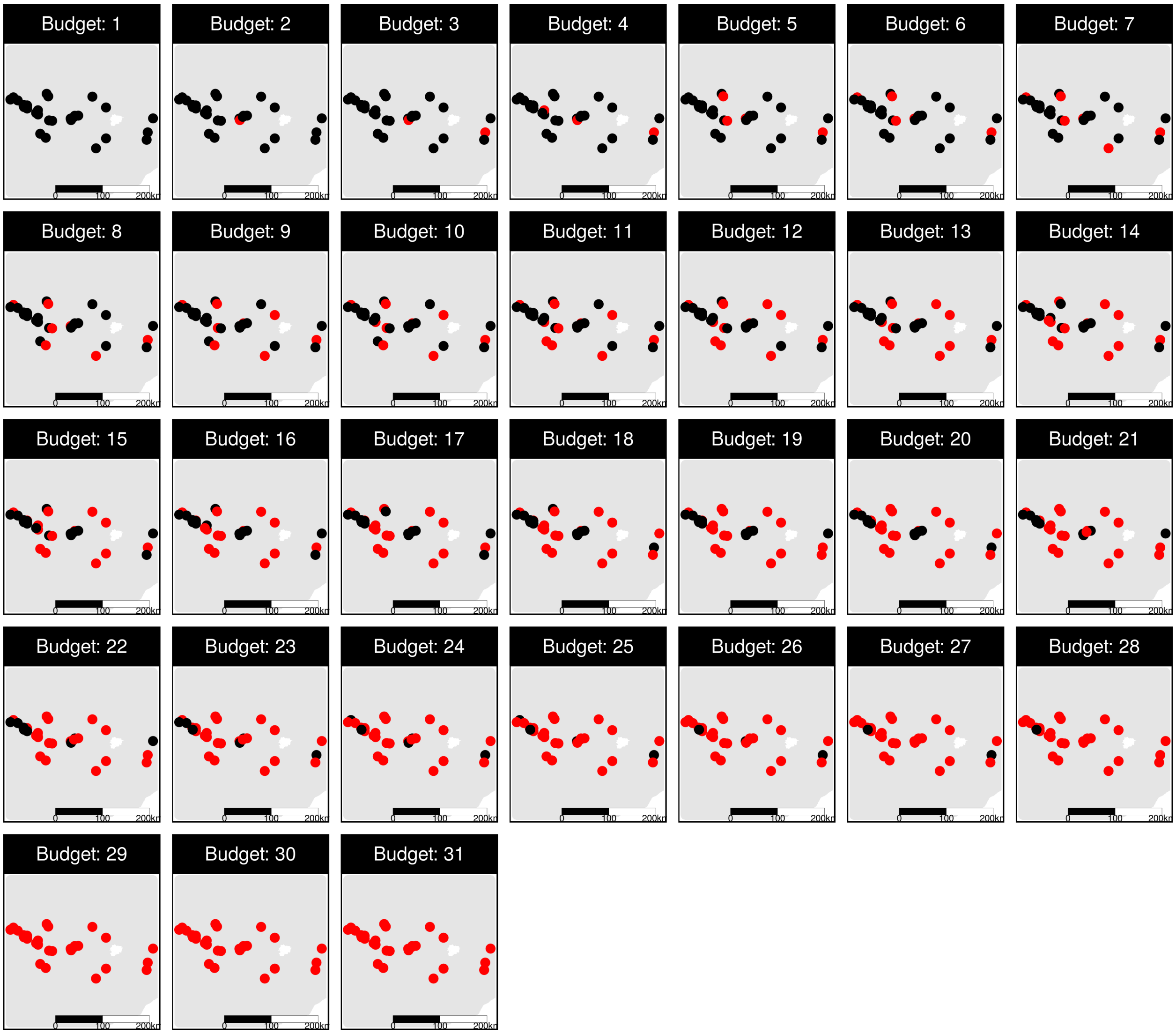

Appendix S58: Site-based prioritisations generated for *Calotriton asper* using genetic distances. Panels correspond to different budgets, red points denote selected sites, and black points denote unselected sites.

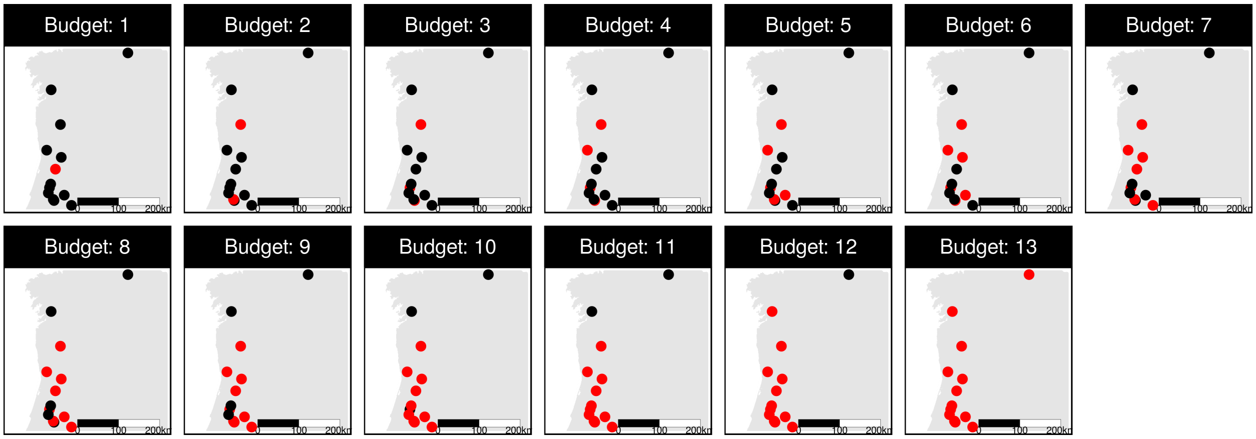

Appendix S59: Site-based prioritisations generated for *Chioglossa lusitanica* using genetic distances. Panels correspond to different budgets, red points denote selected sites, and black points denote unselected sites.

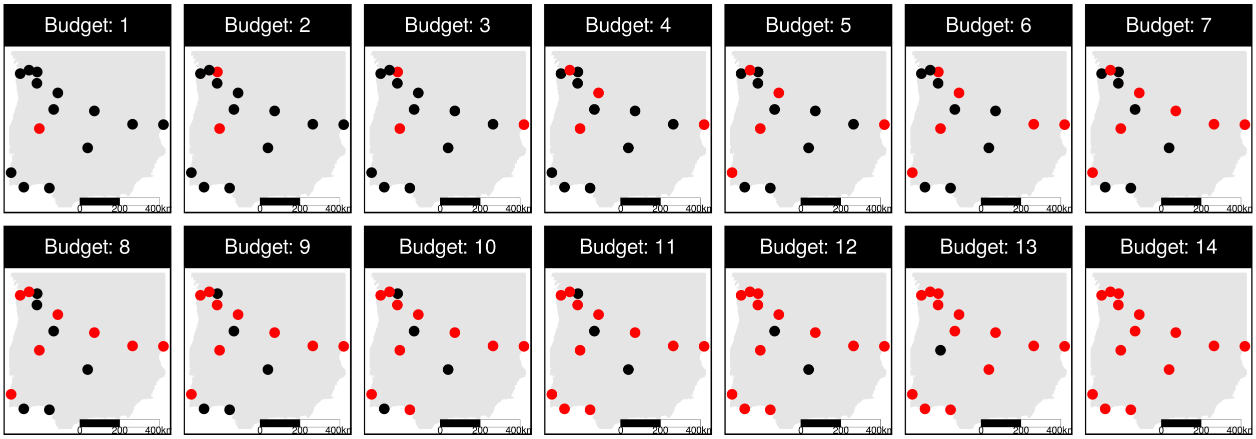

Appendix S60: Site-based prioritisations generated for *Emys orbicularis occidentalis* using genetic distances. Panels correspond to different budgets, red points denote selected sites, and black points denote unselected sites.

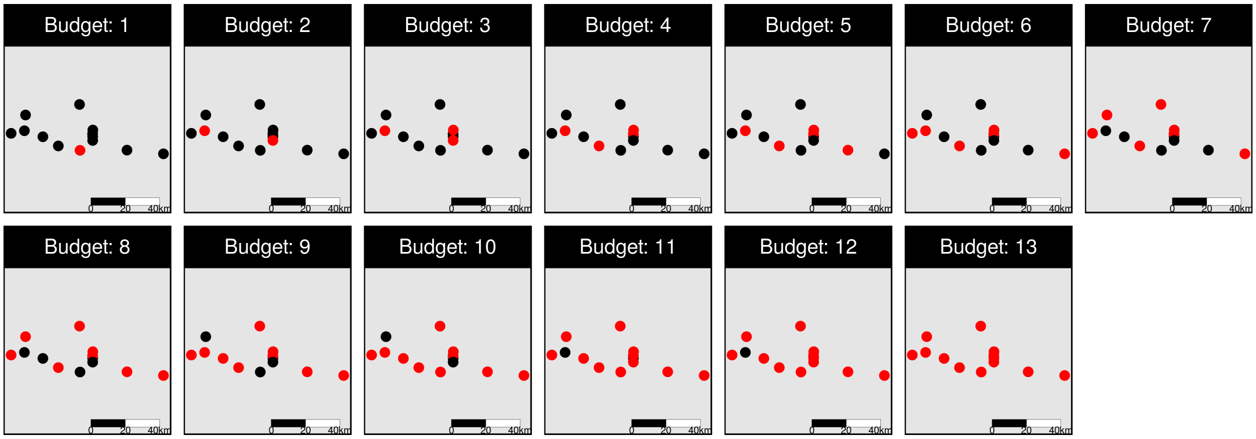

Appendix S61: Site-based prioritisations generated for *Iberolacerta bonnali* using genetic distances. Panels correspond to different budgets, red points denote selected sites, and black points denote unselected sites.

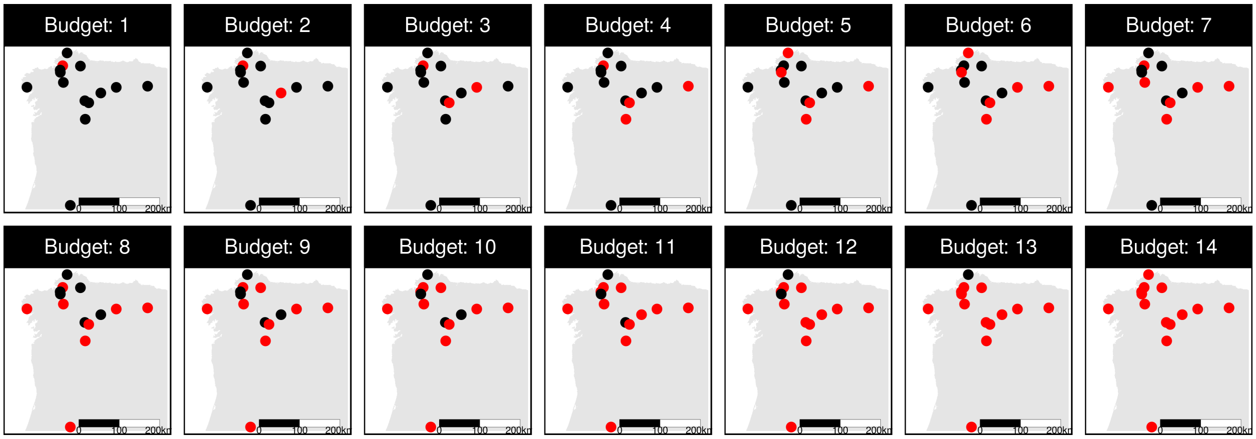

Appendix S62: Site-based prioritisations generated for *Iberolacerta monticola* using genetic distances. Panels correspond to different budgets, red points denote selected sites, and black points denote unselected sites.

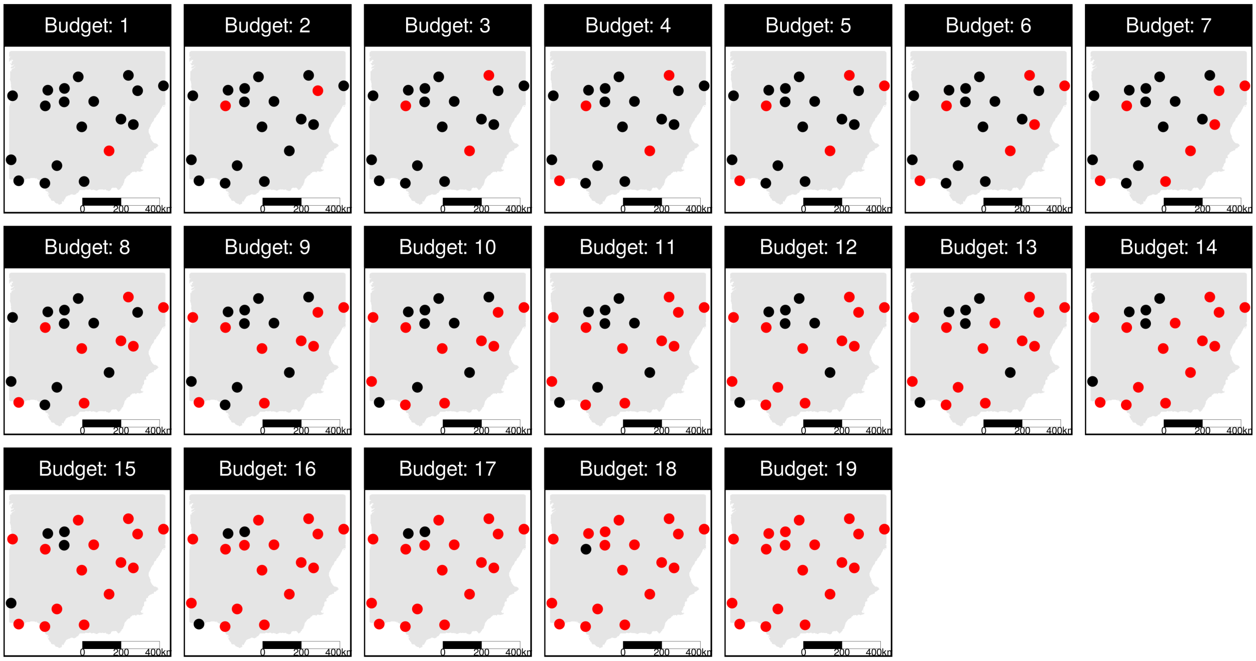

Appendix S63: Site-based prioritisations generated for *Pelobates cultripes* using genetic distances. Panels correspond to different budgets, red points denote selected sites, and black points denote unselected sites.

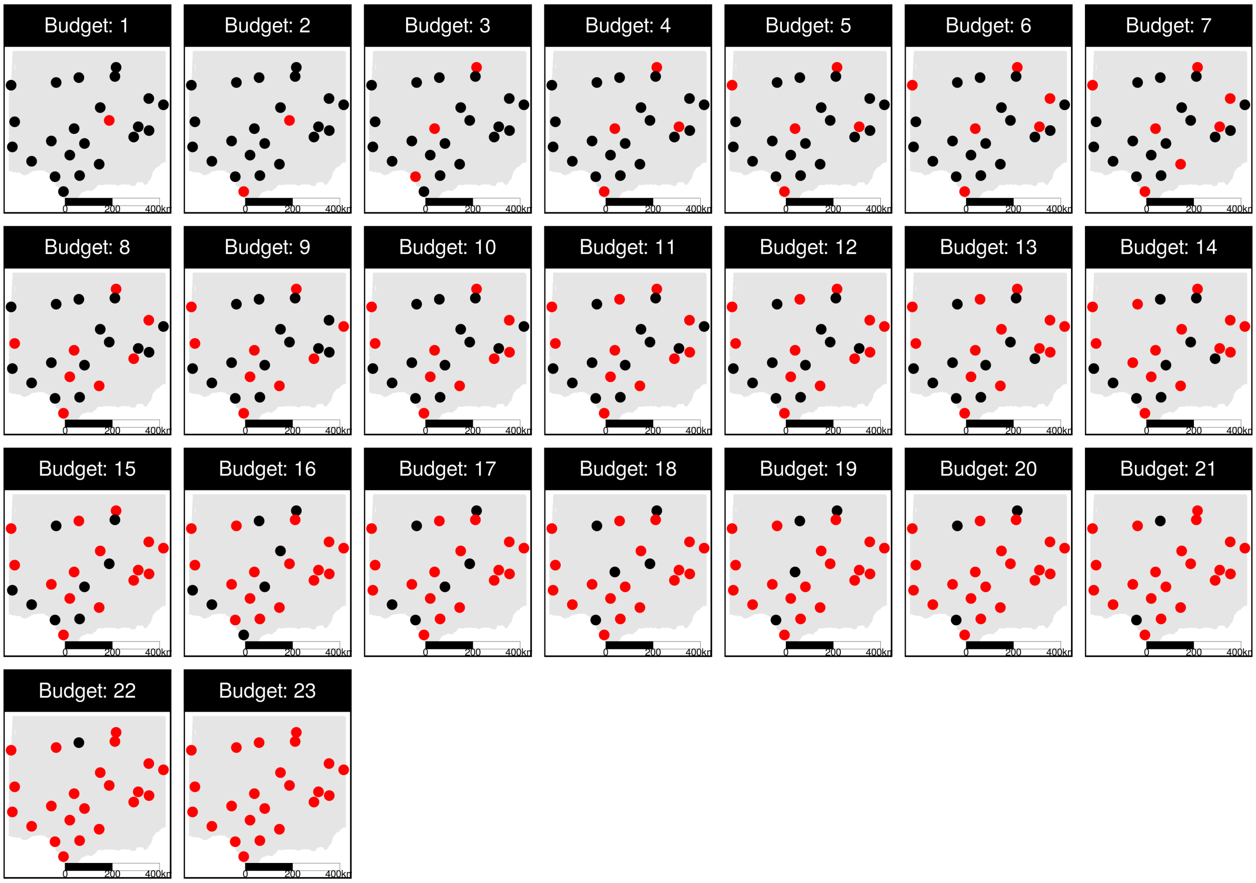

Appendix S64: Site-based prioritisations generated for *Pleurodeles waltl* using genetic distances. Panels correspond to different budgets, red points denote selected sites, and black points denote unselected sites.

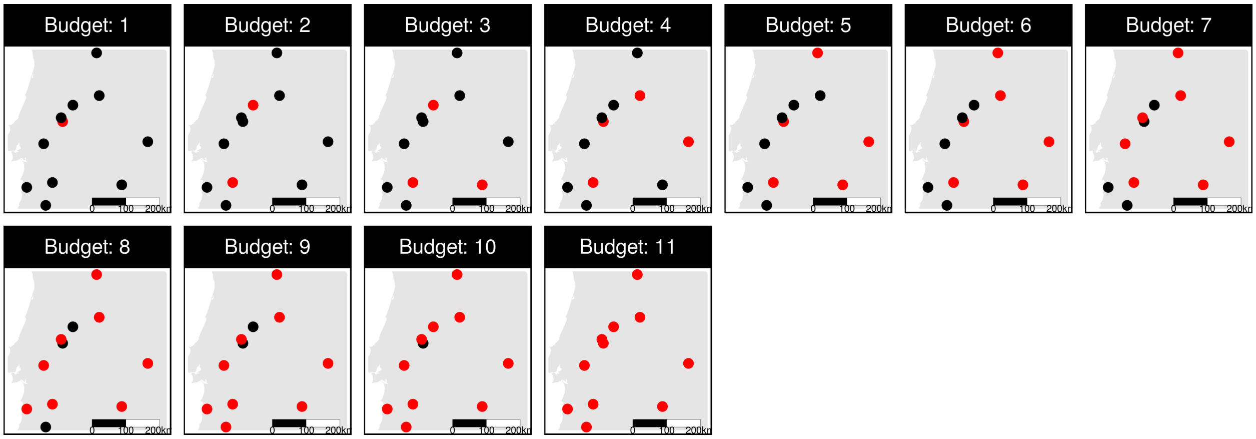

Appendix S65: Site-based prioritisations generated for *Alytes cisternasii* using geographic distances. Panels correspond to different budgets, red points denote selected sites, and black points denote unselected sites.

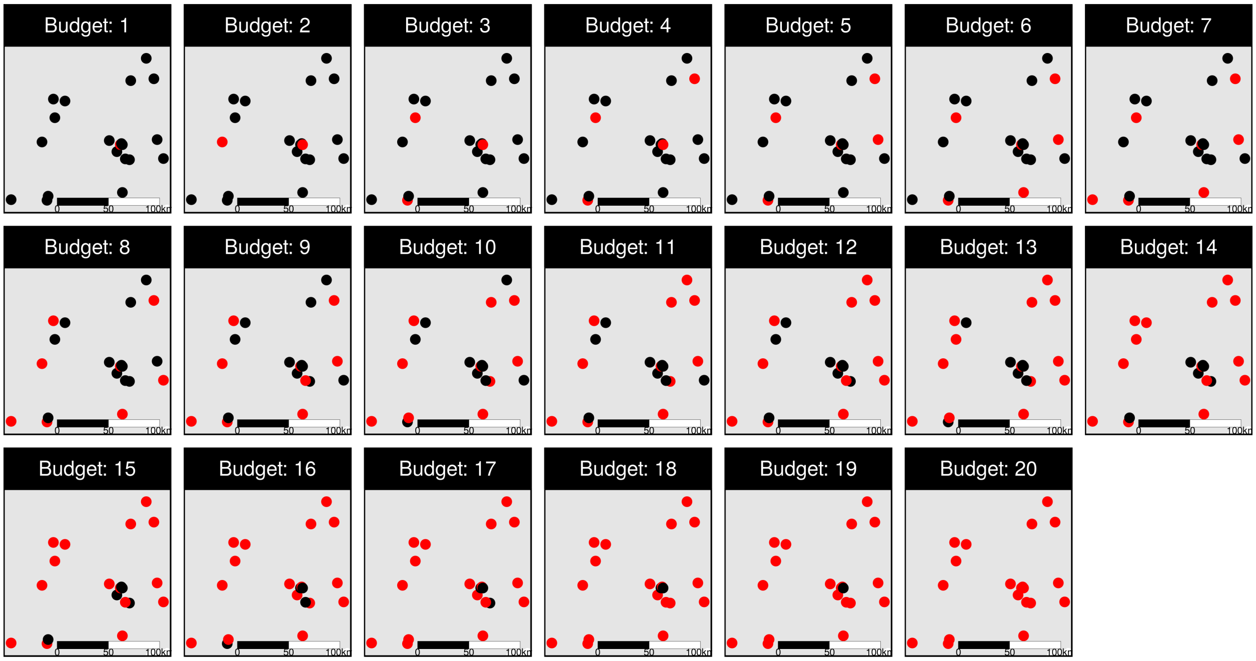

Appendix S66: Site-based prioritisations generated for *Alytes dickhilleni* using geographic distances. Panels correspond to different budgets, red points denote selected sites, and black points denote unselected sites.

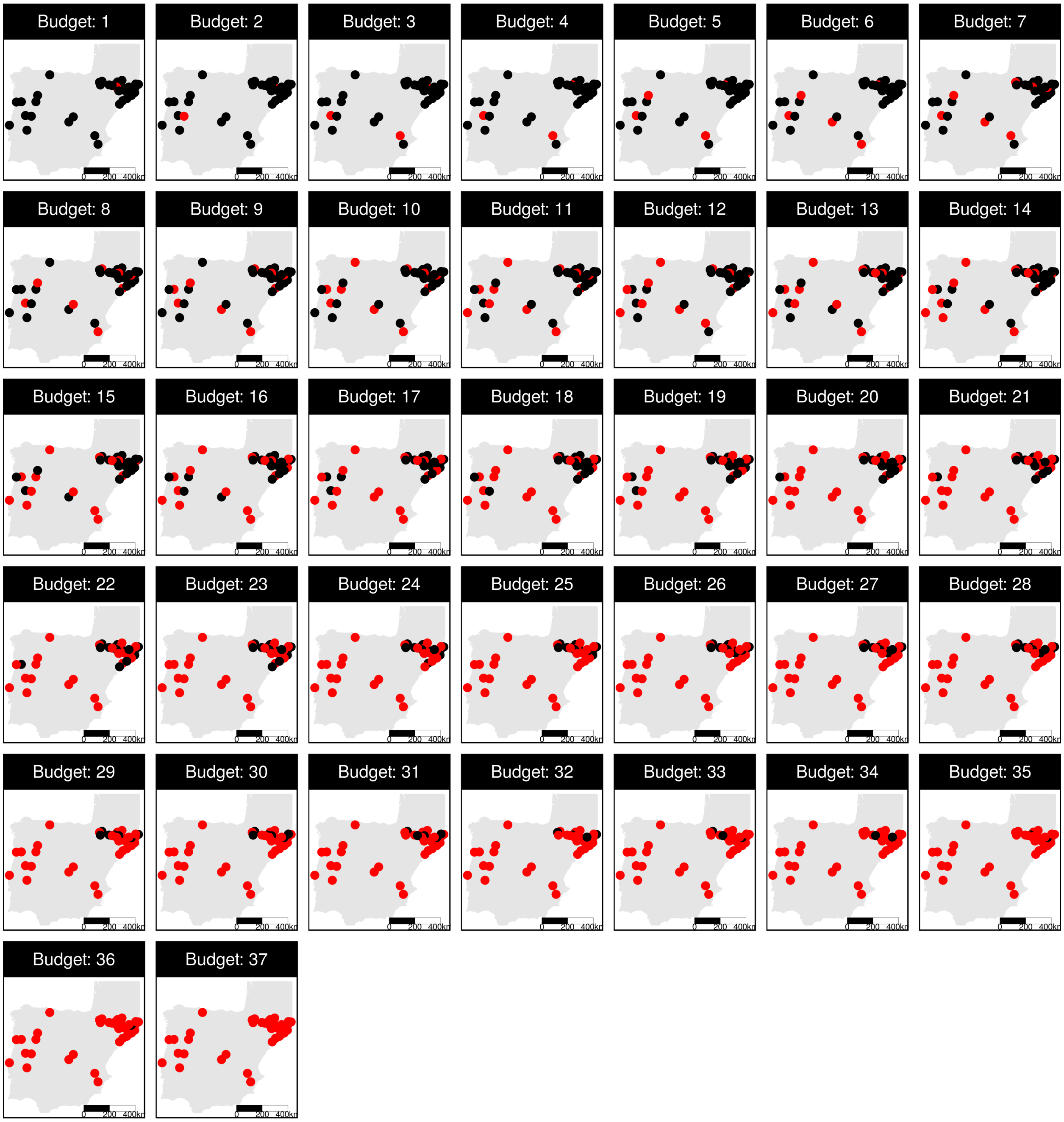

Appendix S67: Site-based prioritisations generated for *Alytes obstetricans* using geographic distances. Panels correspond to different budgets, red points denote selected sites, and black points denote unselected sites.

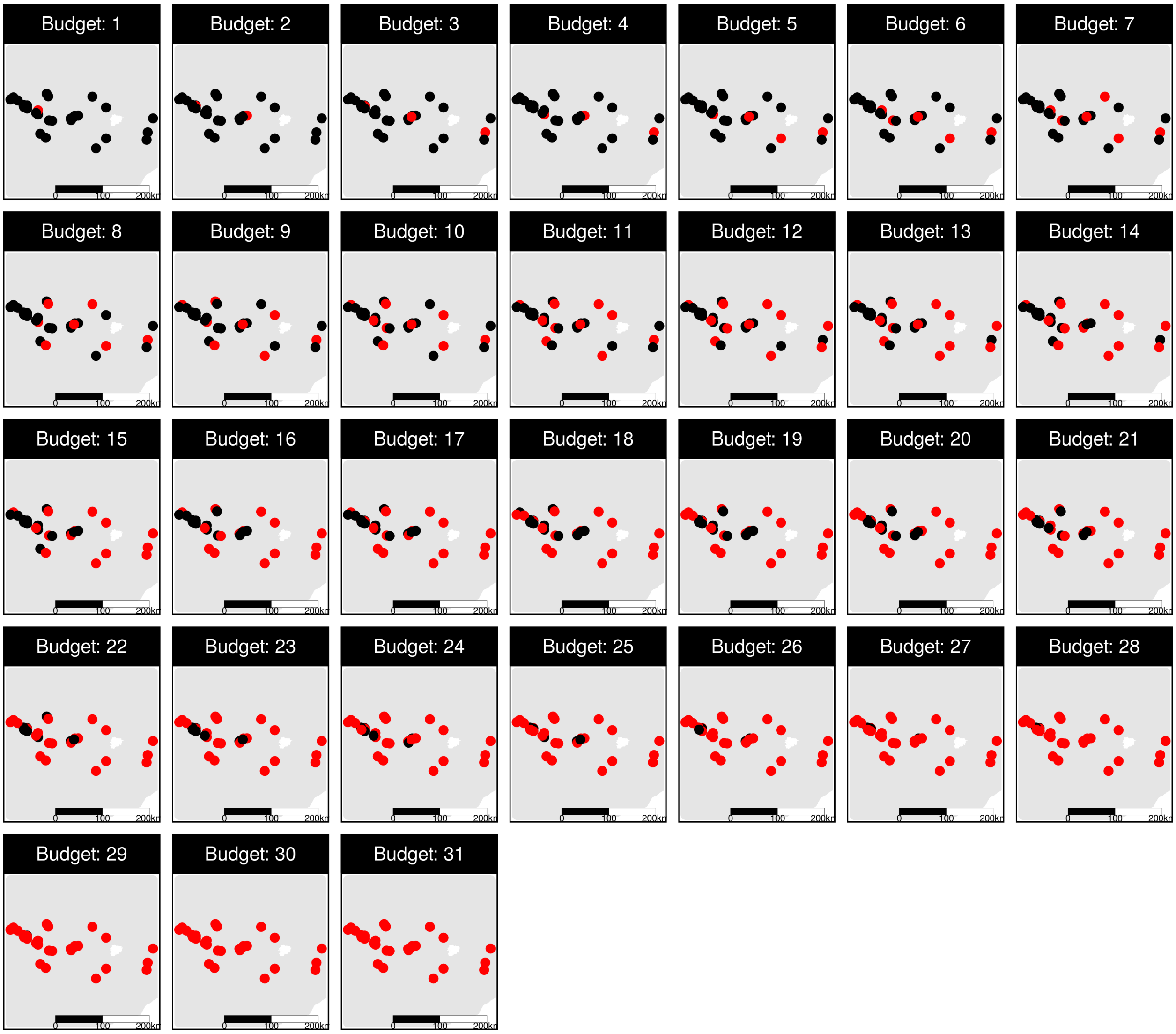

Appendix S68: Site-based prioritisations generated for *Calotriton asper* using geographic distances. Panels correspond to different budgets, red points denote selected sites, and black points denote unselected sites.

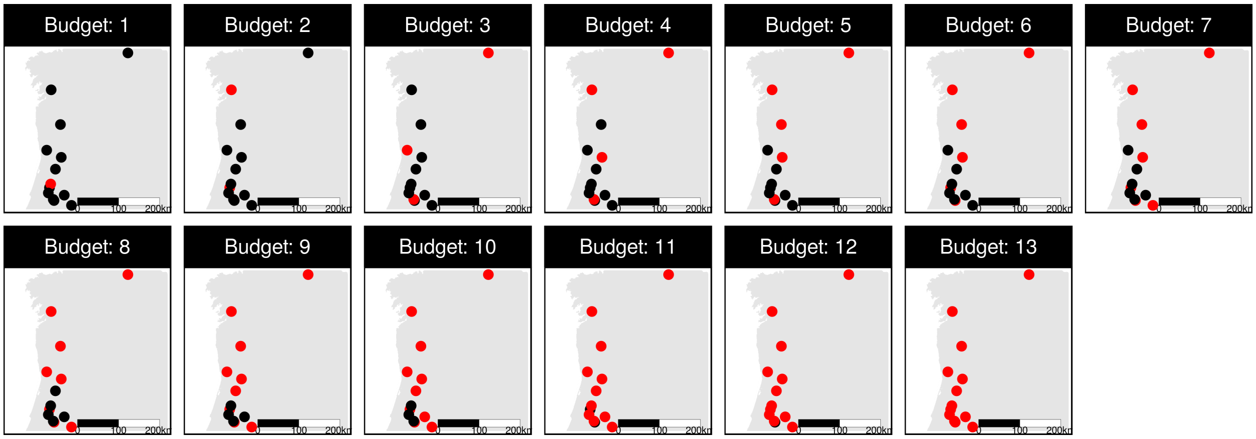

Appendix S69: Site-based prioritisations generated for *Chioglossa lusitanica* using geographic distances. Panels correspond to different budgets, red points denote selected sites, and black points denote unselected sites.

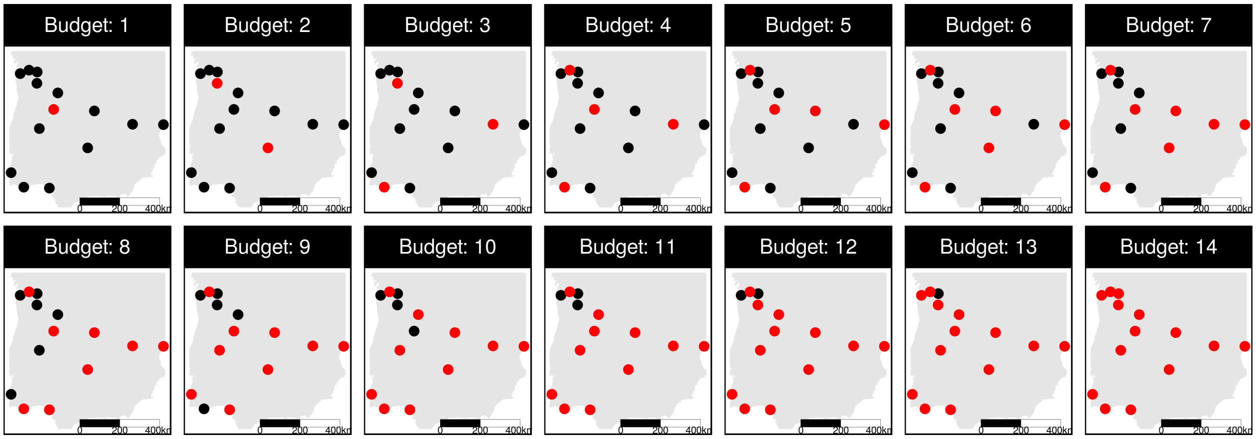

Appendix S70: Site-based prioritisations generated for *Emys orbicularis occidentalis* using geographic distances. Panels correspond to different budgets, red points denote selected sites, and black points denote unselected sites.

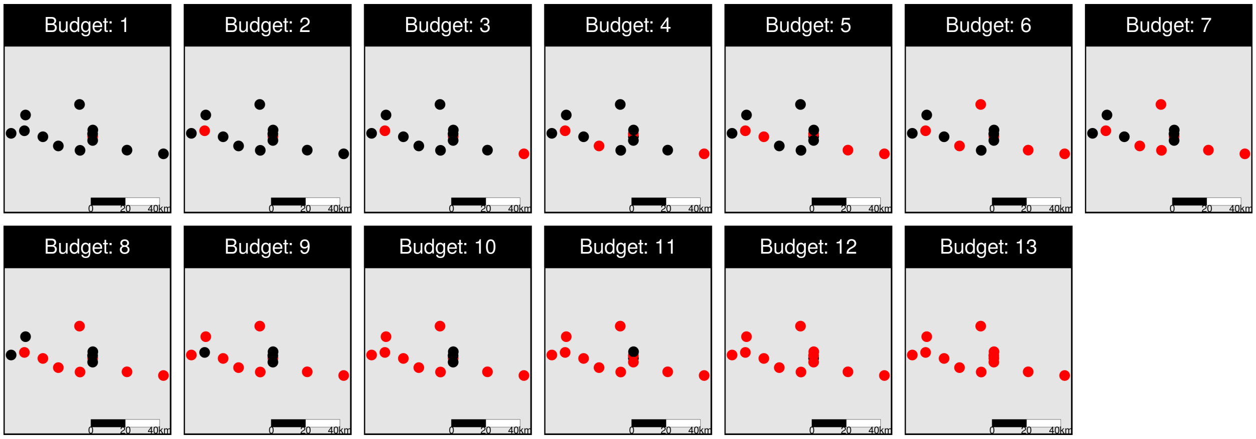

Appendix S71: Site-based prioritisations generated for *Iberolacerta bonnali* using geographic distances. Panels correspond to different budgets, red points denote selected sites, and black points denote unselected sites.

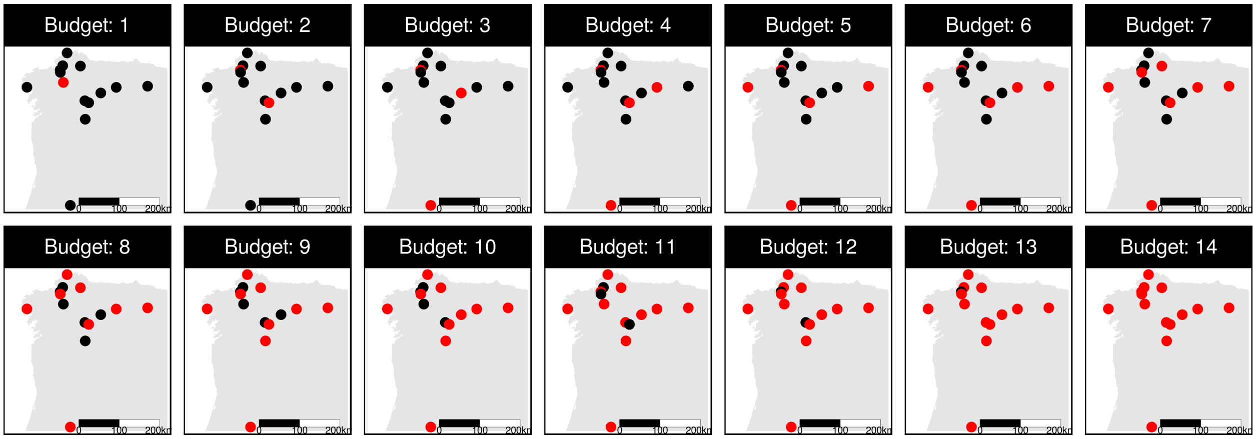

Appendix S72: Site-based prioritisations generated for *Iberolacerta monticola* using geographic distances. Panels correspond to different budgets, red points denote selected sites, and black points denote unselected sites.

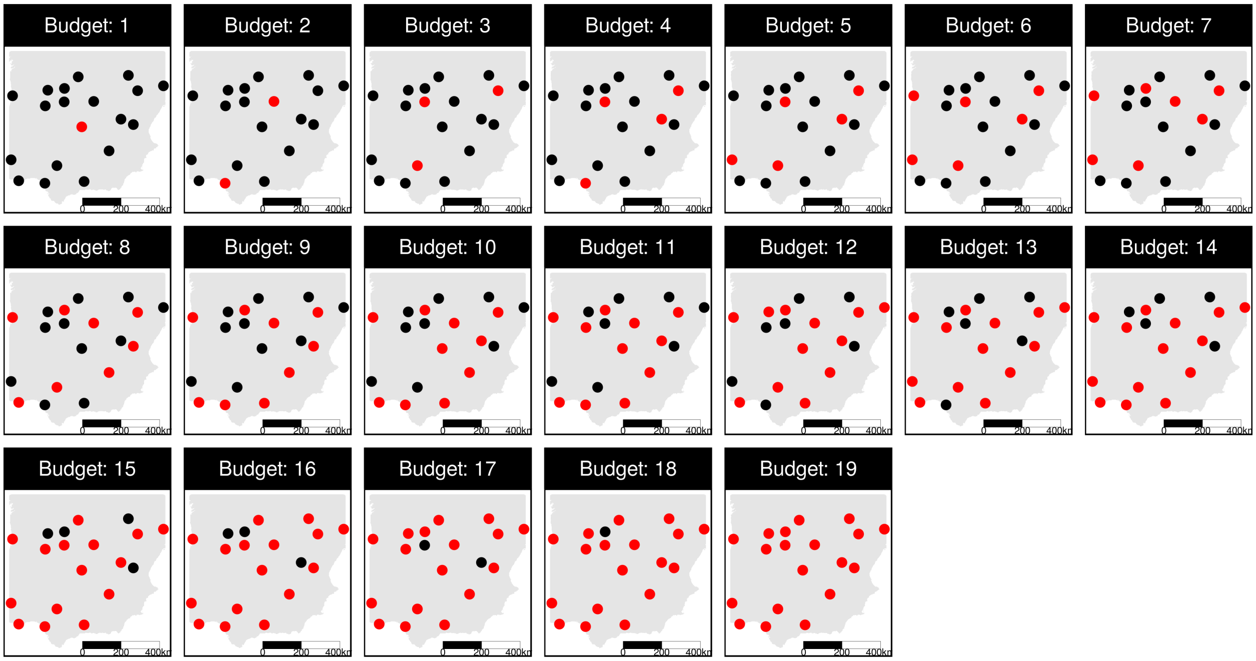

Appendix S73: Site-based prioritisations generated for *Pelobates cultripes* using geographic distances. Panels correspond to different budgets, red points denote selected sites, and black points denote unselected sites.

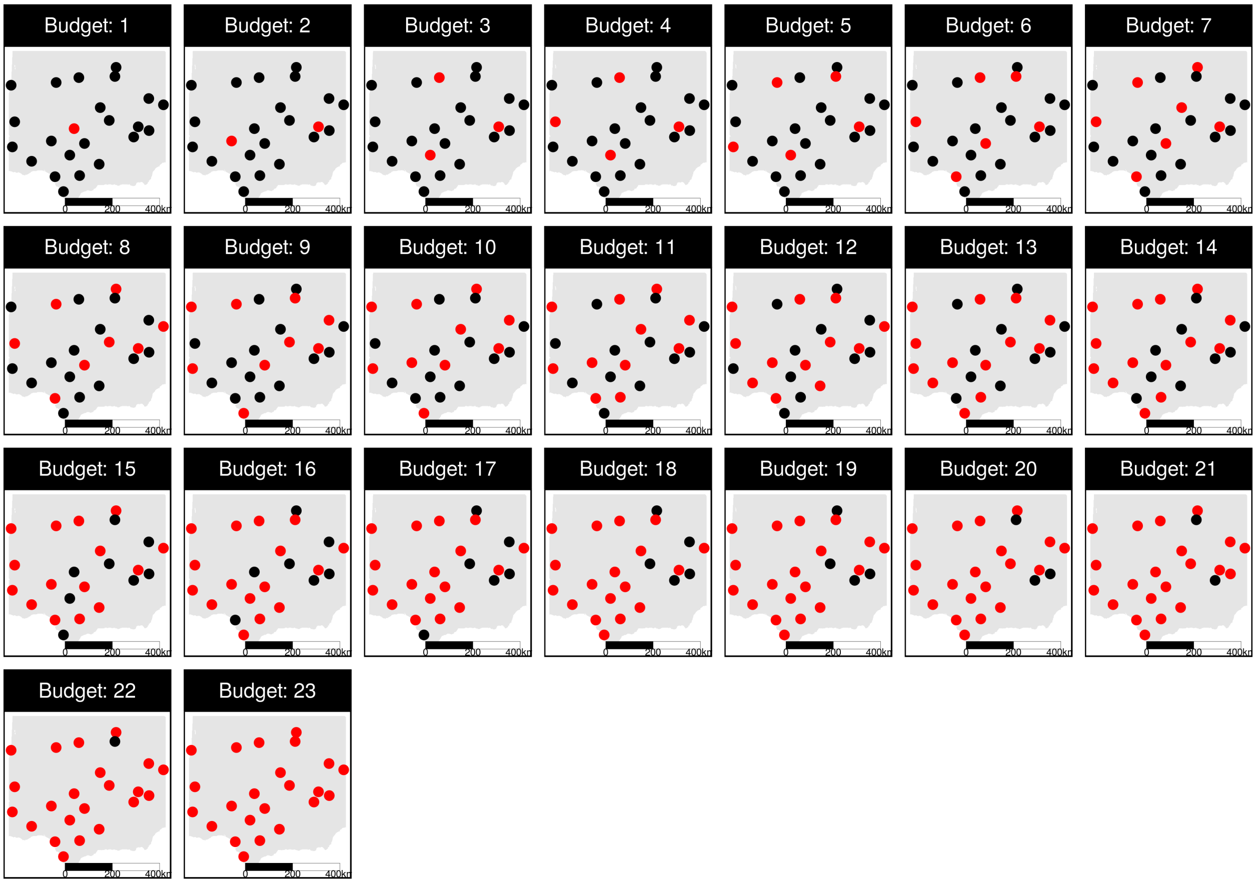

Appendix S74: Site-based prioritisations generated for *Pleurodeles waltl* using geographic distances. Panels correspond to different budgets, red points denote selected sites, and black points denote unselected sites.

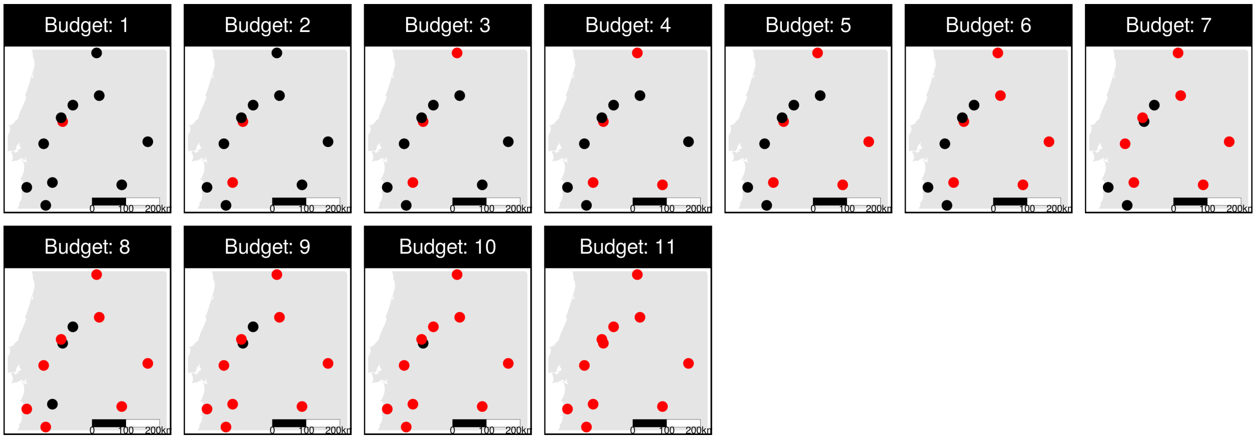

Appendix S75: Site-based prioritisations generated for *Alytes cisternasii* using resistance distances. Panels correspond to different budgets, red points denote selected sites, and black points denote unselected sites.

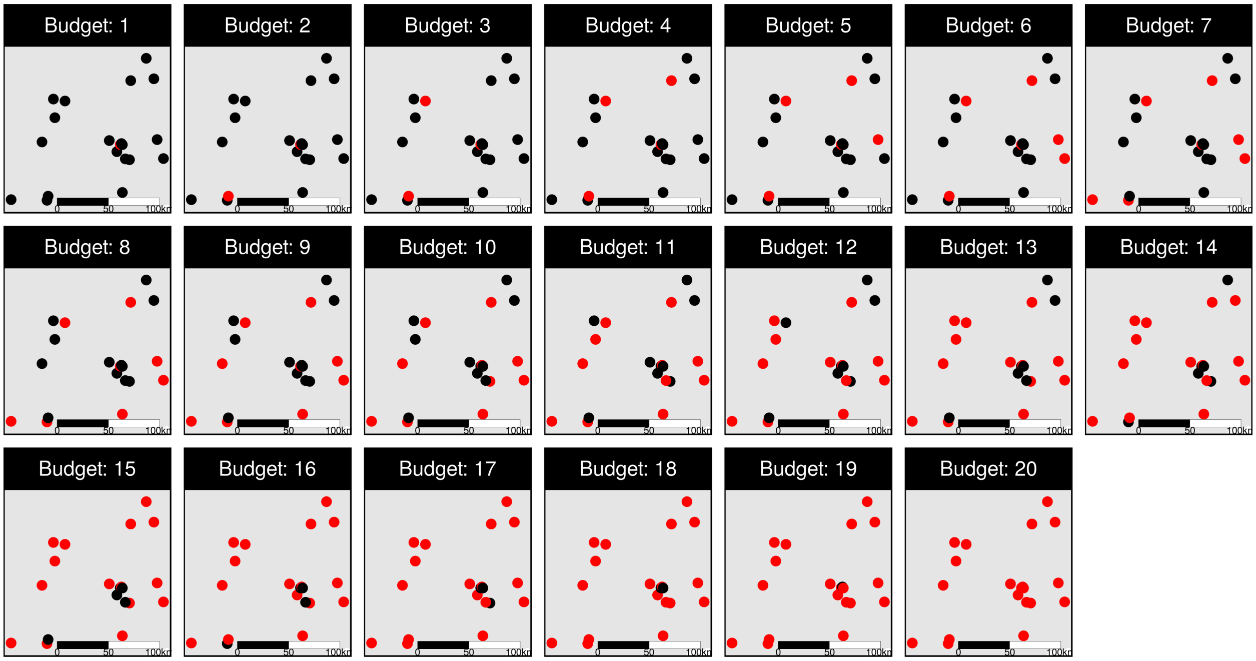

Appendix S76: Site-based prioritisations generated for *Alytes dickhilleni* using resistance distances. Panels correspond to different budgets, red points denote selected sites, and black points denote unselected sites.

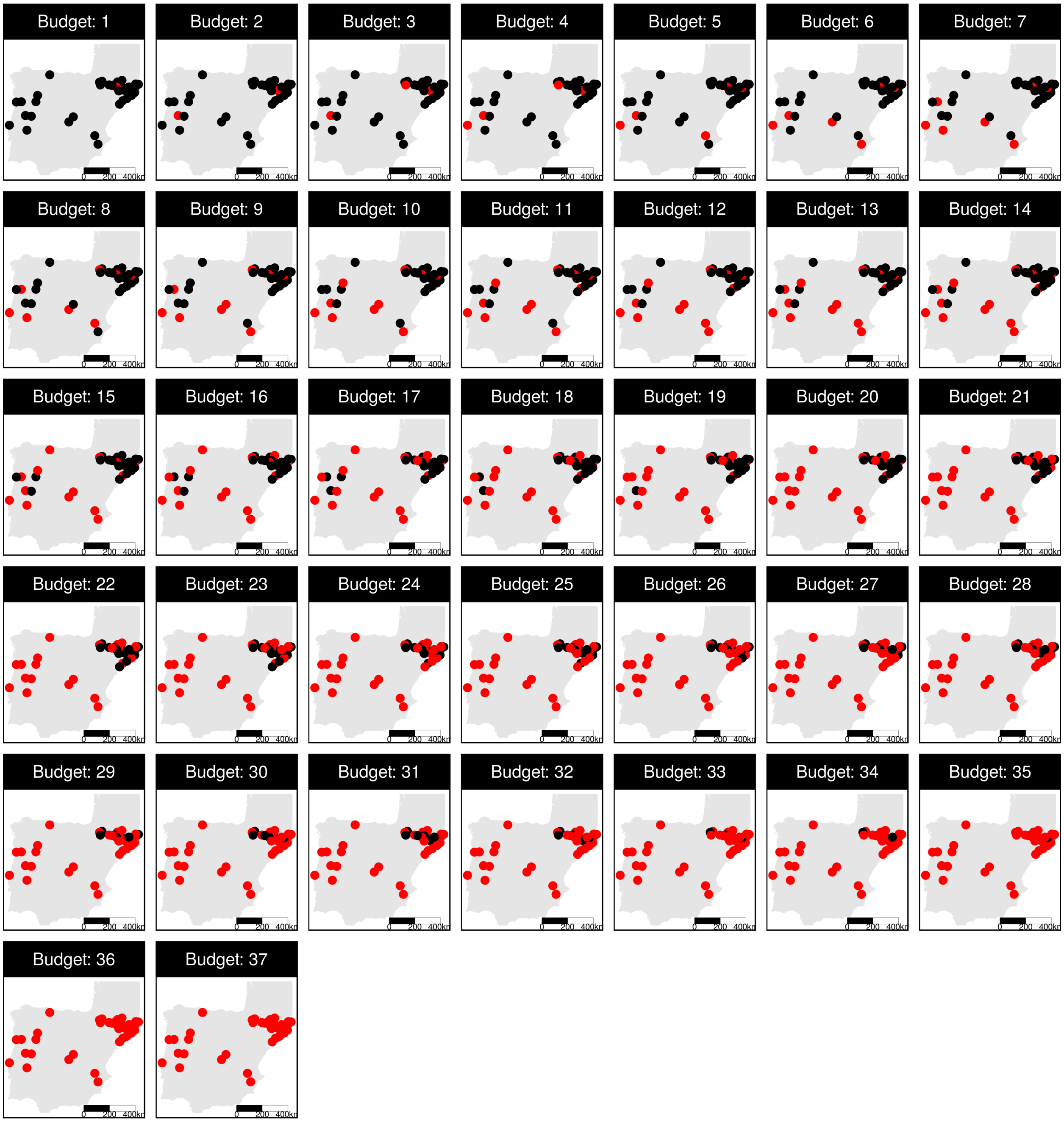

Appendix S77: Site-based prioritisations generated for *Alytes obstetricans* using resistance distances. Panels correspond to different budgets, red points denote selected sites, and black points denote unselected sites.

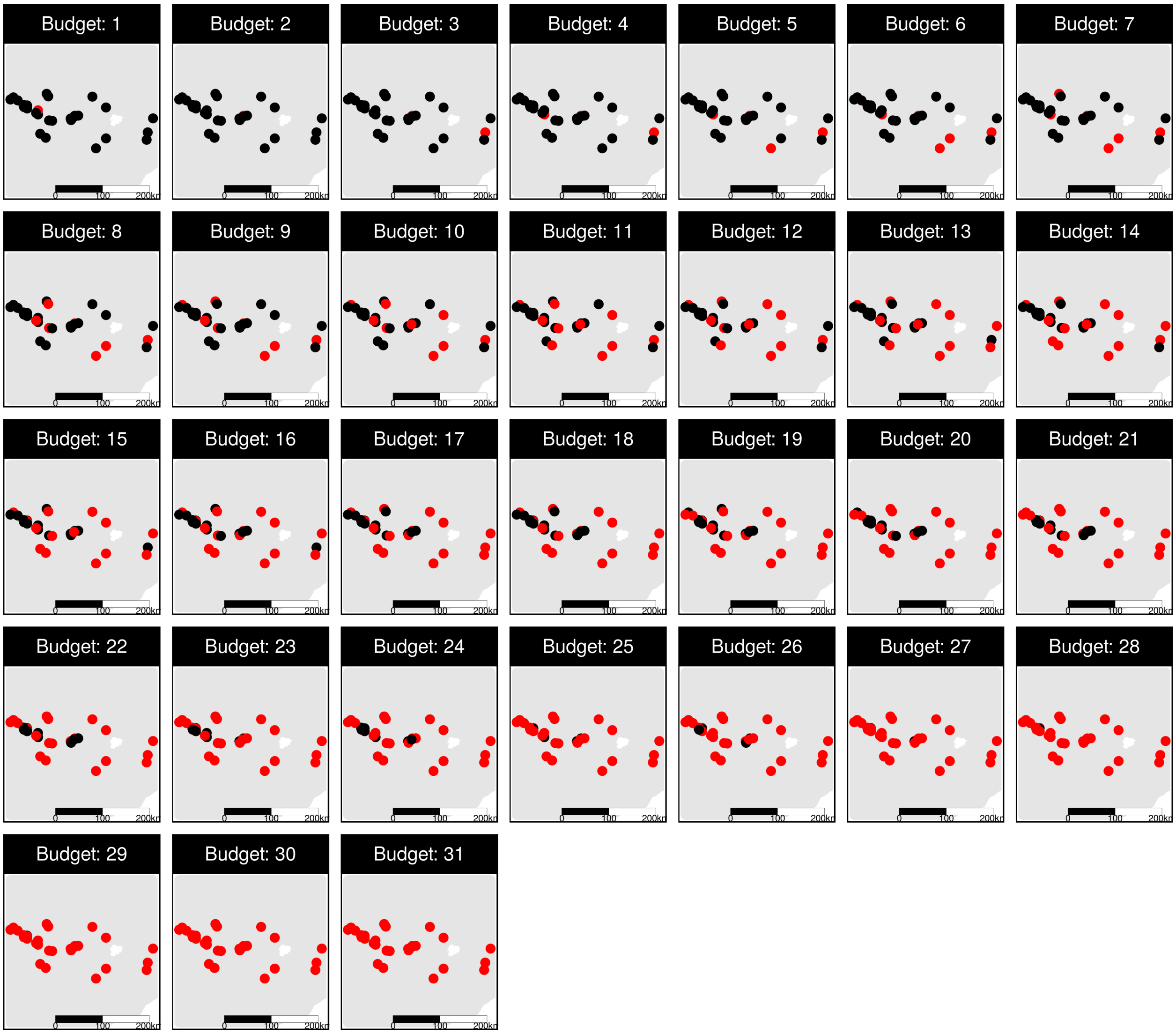

Appendix S78: Site-based prioritisations generated for *Calotriton asper* using resistance distances. Panels correspond to different budgets, red points denote selected sites, and black points denote unselected sites.

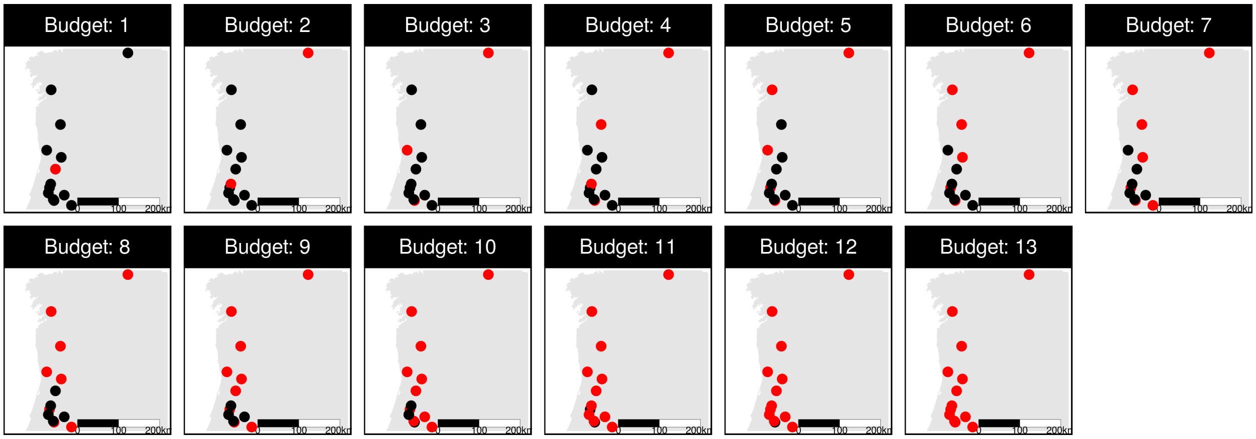

Appendix S79: Site-based prioritisations generated for *Chioglossa lusitanica* using resistance distances. Panels correspond to different budgets, red points denote selected sites, and black points denote unselected sites.

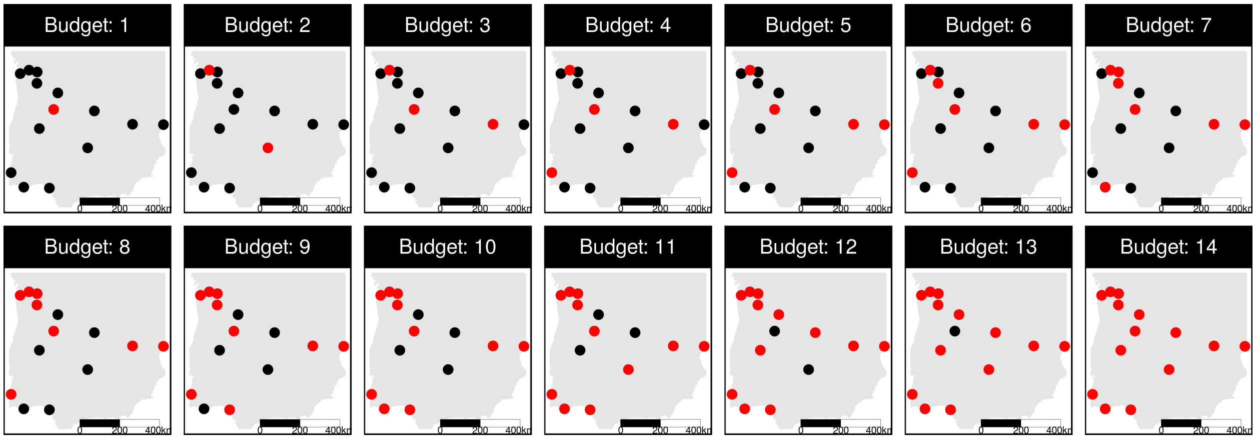

Appendix S80: Site-based prioritisations generated for *Emys orbicularis occidentalis* using resistance distances. Panels correspond to different budgets, red points denote selected sites, and black points denote unselected sites.

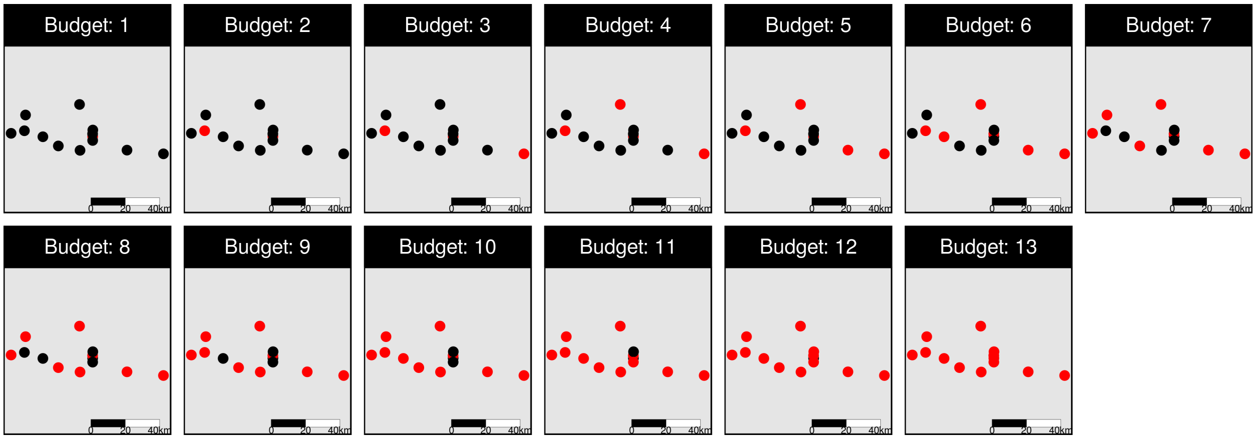

Appendix S81: Site-based prioritisations generated for *Iberolacerta bonnali* using resistance distances. Panels correspond to different budgets, red points denote selected sites, and black points denote unselected sites.

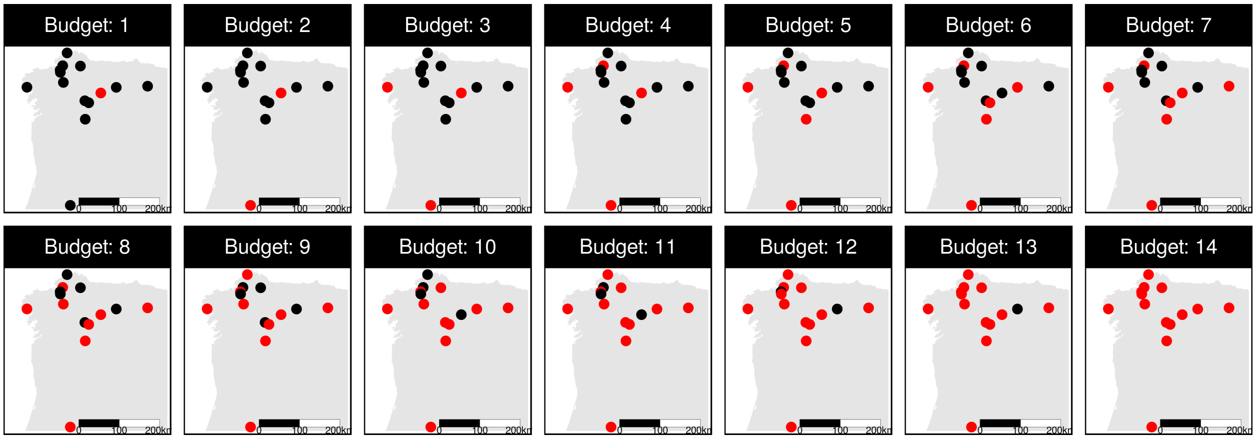

Appendix S82: Site-based prioritisations generated for *Iberolacerta monticola* using resistance distances. Panels correspond to different budgets, red points denote selected sites, and black points denote unselected sites.

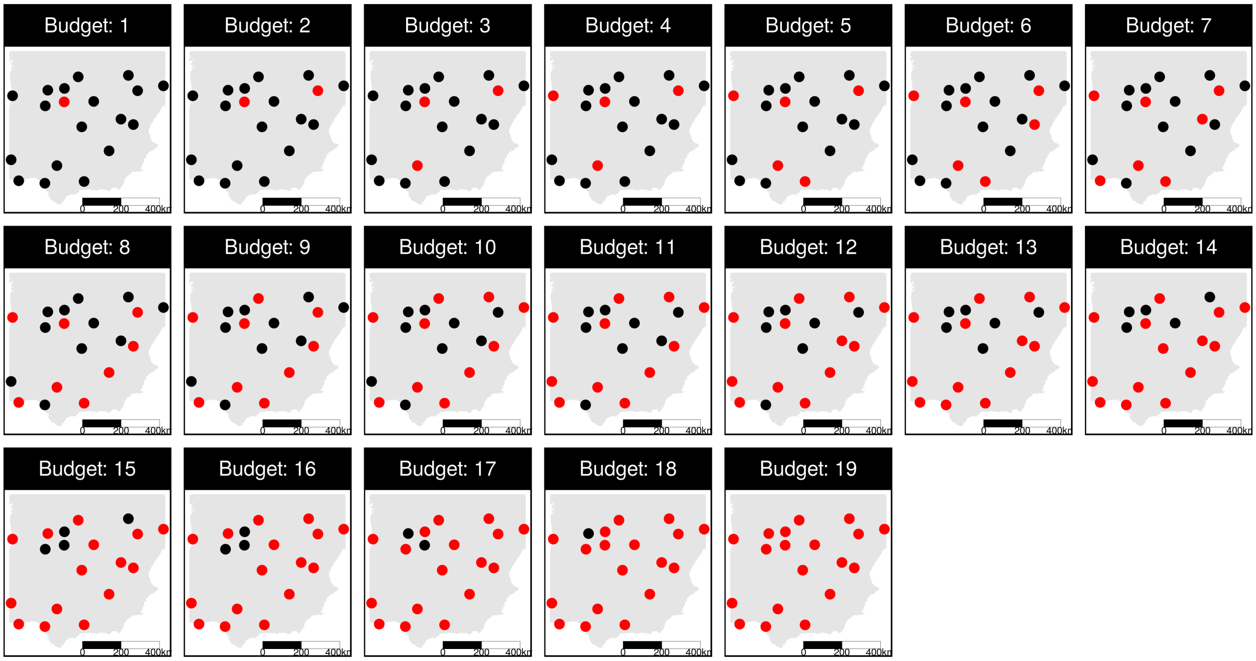

Appendix S83: Site-based prioritisations generated for *Pelobates cultripes* using resistance distances. Panels correspond to different budgets, red points denote selected sites, and black points denote unselected sites.

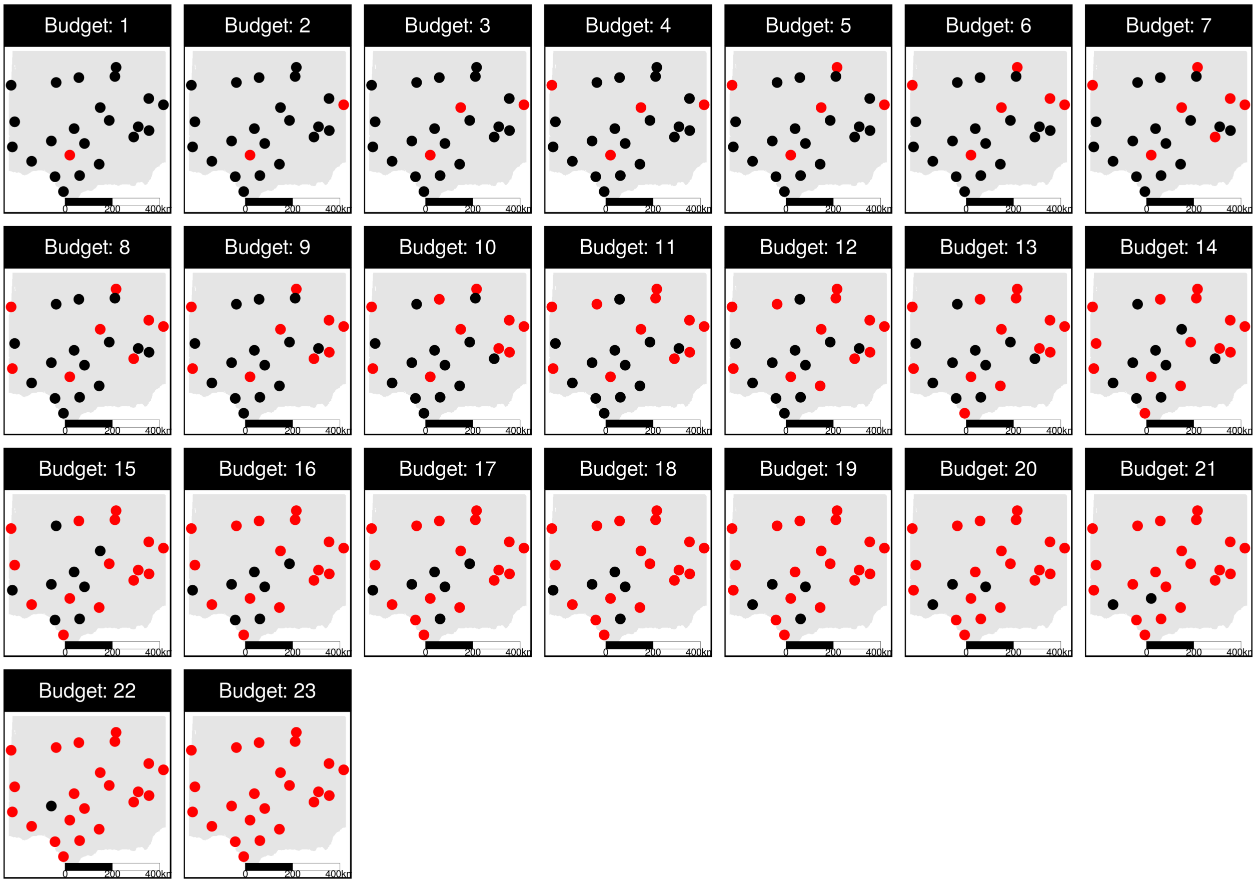

Appendix S84: Site-based prioritisations generated for *Pleurodeles waltl* using resistance distances. Panels correspond to different budgets, red points denote selected sites, and black points denote unselected sites.

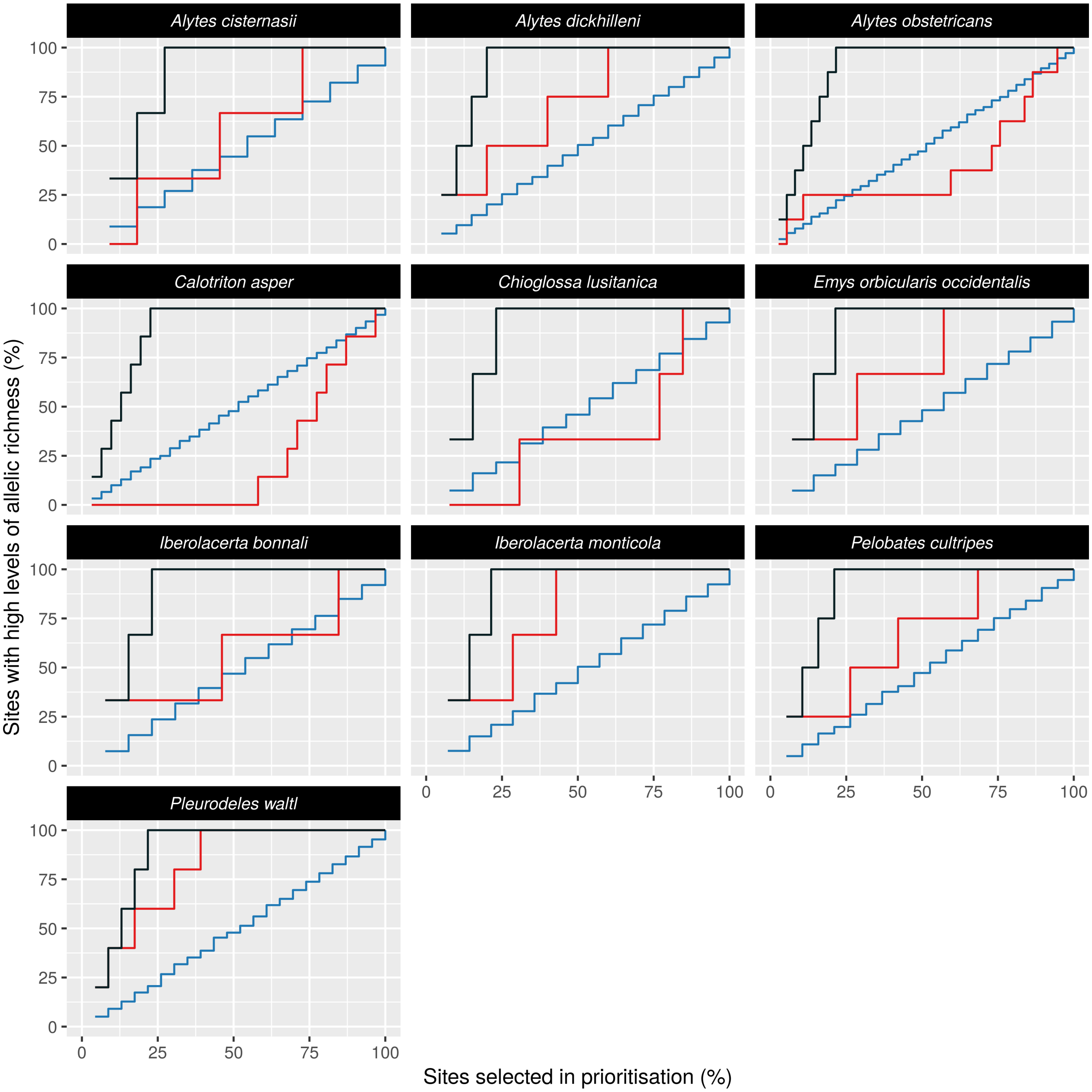

Appendix S85: The relationship between the percentage of sites with high allelic richness that are held in a prioritisation and the approach used to generate it. Since percentages are expressed relative to the total number of sites with high allelic richness, prioritisations which contain all of these sites – even if they include additional sites with low allelic richness – are assigned a 100% value. Each panel corresponds to a different species, and lines correspond to different prioritisations. Black lines correspond to prioritisations generated using measures of allelic richness. Red lines correspond to prioritisations generated using long-term environmental suitability. Blue lines show the average performance of prioritisations generated by randomly selecting sites. As a consequence, there is the greatest support for the (red) surrogate-based prioritisations performing well when they approximate the (black) genetic-based prioritisations and are different from the (blue) randomly generated prioritisations.

Appendix S86: Summary of the generalized linear mixed effects model used to describe the performance of different prioritization approaches for representing site-level genetic diversity. Specifically, this table contains the fixed effects. As described in the main text, the response variable for this model was the proportion of site-scale genetic diversity that a given prioritization secured. It was fit using a continuous predictor variable describing the cost of each prioritization ("cost" term), a categorical predictor variable describing the method used to generate each prioritization ("method" terms, with the following levels: "random" and "long-term environmental suitability"), and a predictor variable accounting for the interaction between the two previous variables ("cost:method" terms). The model intercept corresponds to prioritizations generated using genetic diversity directly. Note that the model was fit using a logit link when interpreting these coefficients.

Term	Estimate	Standard error
intercept	-2.857	0.958
cost	27.486	7.173
method (method = random)	0.245	1.037
method (method = long-term environmental suitability)	0.829	1.022
cost:method (method = random)	-22.084	7.205
cost:method (method = long-term environmental suitability)	-22.571	7.203

Appendix S87: Performance of prioritisations generated for site-level genetic diversity. Data show mean and standard deviation in performance values for each species. Specifically, performance values are expressed as the proportion of sites with high genetic diversity secured in a given prioritisation relative to those in optimal prioritisations based on genetic data with the same number of selected sites. The results presented in the main text were based on an 80th percentile threshold for defining sites with high site-level genetic diversity. To assess the sensitivity of these results to this particular threshold, data are also shown for two additional thresholds.

Species	Prioritisation approach	Threshold	Performance
Alytes cisternasii	genetic	70	100% ± 0
		80	100% ± 0
		90	100% ± 0
	random	70	59.38% ± 0.33
		80	57.07% ± 0.36
		90	55.36% ± 0.4
	long-term environmental suitability	70	55.3% ± 0.31
		80	65.15% ± 0.34
		90	63.64% ± 0.32
Alytes dickhilleni	genetic	70	100% ± 0
		80	100% ± 0
		90	100% ± 0
	random	70	56.36% ± 0.31
		80	54.07% ± 0.34
		90	52.85% ± 0.4
	long-term environmental suitability	70	74.25% ± 0.15
		80	79.17% ± 0.24
		90	55% ± 0.46
Alytes obstetricans	genetic	70	100% ± 0
		80	100% ± 0
		90	100% ± 0
	random	70	55.27% ± 0.29
		80	53.27% ± 0.31
		90	51.68% ± 0.35
	long-term environmental suitability	70	56.88% ± 0.24
		80	44.53% ± 0.26
		90	41.89% ± 0.33
Calotriton asper	genetic	70	100% ± 0
		80	100% ± 0
		90	100% ± 0
	random	70	56.38% ± 0.28
		80	53.87% ± 0.31
		90	52.22% ± 0.35
	long-term environmental suitability	70	35.48% ± 0.36
		80	26.27% ± 0.36
		90	27.42% ± 0.38
Chioglossa lusitanica	genetic	70	100% ± 0
		80	100% ± 0

Species	Prioritisation approach	Threshold	Performance
		90	100% ± 0
	random	70	57.14% ± 0.34
		80	55.67% ± 0.36
		90	54.35% ± 0.4
	long-term environmental suitability	70	48.08% ± 0.37
		80	43.59% ± 0.37
		90	26.92% ± 0.44
Emys orbicularis occidentalis	genetic	70	100% ± 0
		80	100% ± 0
		90	100% ± 0
	random	70	56.74% ± 0.34
		80	54.94% ± 0.36
		90	53.73% ± 0.4
	long-term environmental suitability	70	75.6% ± 0.27
		80	82.14% ± 0.23
		90	64.29% ± 0.41
Iberolacerta bonnali	genetic	70	100% ± 0
		80	100% ± 0
		90	100% ± 0
	random	70	57.69% ± 0.34
		80	55.9% ± 0.36
		90	54.9% ± 0.4
	long-term environmental suitability	70	64.1% ± 0.29
		80	67.95% ± 0.26
		90	42.31% ± 0.4
Iberolacerta monticola	genetic	70	100% ± 0
		80	100% ± 0
		90	100% ± 0
	random	70	56.74% ± 0.34
		80	55.28% ± 0.36
		90	54.08% ± 0.4
	long-term environmental suitability	70	79.17% ± 0.24
		80	86.9% ± 0.23
		90	92.86% ± 0.18
Pelobates cultripes	genetic	70	100% ± 0
		80	100% ± 0
		90	100% ± 0
	random	70	56.79% ± 0.31
		80	54.41% ± 0.34
		90	52.83% ± 0.4
	long-term environmental suitability	70	77.72% ± 0.2
		80	75.44% ± 0.26
		90	84.21% ± 0.24
Pleurodeles waltl	genetic	70	100% ± 0
		80	100% ± 0
		90	100% ± 0

Species	Prioritisation approach	Threshold	Performance
	random	70	56.39% ± 0.3
		80	54.3% ± 0.33
		90	52.86% ± 0.37
	long-term environmental suitability	70	90.85% ± 0.11
		80	92.25% ± 0.14
		90	83.33% ± 0.27

Appendix S88: One-sided Spearman's rank correlation tests between the allelic richness measured at a site and the long-term environmental suitability estimated for the same site.

Species	r	Statistic	P
Alytes cisternasii	-0.14	250.00	0.69
Alytes dickhilleni	0.40	795.79	0.079
Alytes obstetricans	-0.03	8674.00	0.87
Calotriton asper	-0.25	6194.62	0.18
Chioglossa lusitanica	-0.18	431.09	0.55
Emys orbicularis occidentalis	0.53	212.00	0.052
Iberolacerta bonnali	0.28	261.44	0.35
Iberolacerta monticola	0.20	364.90	0.50
Pelobates cultripes	0.37	720.00	0.12
Pleurodeles waltl	0.75	504.00	$<$ **0.001**

Appendix S89: Performance of prioritisations for representing site-level genetic diversity compared to randomly generated prioritisations. Data show mean and standard deviation in performance values for each species. Specifically, performance values are expressed as the proportion of sites with high genetic diversity secured in a given prioritisation relative to those in optimal prioritisations based on genetic data with the same number of selected sites. The results presented in the main text were based on an 80th percentile threshold for defining sites with high site-level genetic diversity. To assess the sensitivity of these results to this particular threshold, data are also shown for two additional thresholds.

Species	Prioritisation approach	Threshold	Performance
Alytes cisternasii	genetic	70	68.63% ± 0.9
		80	59.11% ± 0.77
		90	35.27% ± 0.48
	random	70	0% ± 0
		80	0% ± 0
		90	0% ± 0
	long-term environmental suitability	70	-3.73% ± 0.37
		80	12.85% ± 0.47
		90	2.55% ± 0.36
Alytes dickhilleni	genetic	70	94.68% ± 1.24
		80	82.56% ± 1.06
		90	34.16% ± 0.47
	random	70	0% ± 0
		80	0% ± 0
		90	0% ± 0
	long-term environmental suitability	70	38.33% ± 0.68
		80	40.97% ± 0.64
		90	-0.94% ± 0.52
Alytes obstetricans	genetic	70	117.48% ± 1.52
		80	125.7% ± 1.69
		90	86.48% ± 1.11
	random	70	0% ± 0
		80	0% ± 0
		90	0% ± 0
	long-term environmental suitability	70	8.6% ± 0.58
		80	-10.95% ± 0.42
		90	-18.2% ± 0.36
Calotriton asper	genetic	70	107.91% ± 1.38
		80	115.6% ± 1.54
		90	85.31% ± 1.1
	random	70	0% ± 0
		80	0% ± 0
		90	0% ± 0
	long-term environmental suitability	70	-44.39% ± 0.41
		80	-58.24% ± 0.44
		90	-47.83% ± 0.49
Chioglossa lusitanica	genetic	70	73.81% ± 0.96

Species	Prioritisation approach	Threshold	Performance
		80	60.75% ± 0.78
		90	34.93% ± 0.48
	random	70	0% ± 0
		80	0% ± 0
		90	0% ± 0
	long-term environmental suitability	70	-11.96% ± 0.46
		80	-19.38% ± 0.4
		90	-40.38% ± 0.54
Emys orbicularis occidentalis	genetic	70	76.42% ± 0.99
		80	61.4% ± 0.79
		90	34.18% ± 0.47
	random	70	0% ± 0
		80	0% ± 0
		90	0% ± 0
	long-term environmental suitability	70	26.37% ± 0.51
		80	33.94% ± 0.56
		90	8.17% ± 0.47
Iberolacerta bonnali	genetic	70	73.26% ± 0.95
		80	60.1% ± 0.78
		90	34.78% ± 0.48
	random	70	0% ± 0
		80	0% ± 0
		90	0% ± 0
	long-term environmental suitability	70	1.11% ± 0.36
		80	7.47% ± 0.38
		90	-20.08% ± 0.44
Iberolacerta monticola	genetic	70	76.64% ± 0.99
		80	61.79% ± 0.8
		90	34.36% ± 0.47
	random	70	0% ± 0
		80	0% ± 0
		90	0% ± 0
	long-term environmental suitability	70	34.7% ± 0.61
		80	42.97% ± 0.67
		90	29.65% ± 0.46
Pelobates cultripes	genetic	70	93.89% ± 1.23
		80	81.07% ± 1.05
		90	34.66% ± 0.48
	random	70	0% ± 0
		80	0% ± 0
		90	0% ± 0
	long-term environmental suitability	70	38.49% ± 0.62
		80	32.3% ± 0.58
		90	22.19% ± 0.43
Pleurodeles waltl	genetic	70	99.49% ± 1.31
		80	96.07% ± 1.25

Species	Prioritisation approach	Threshold	Performance
		90	63.55% ± 0.81
	random	70	0% ± 0
		80	0% ± 0
		90	0% ± 0
	long-term environmental suitability	70	73.81% ± 0.98
		80	76.13% ± 0.99
		90	47.32% ± 0.69

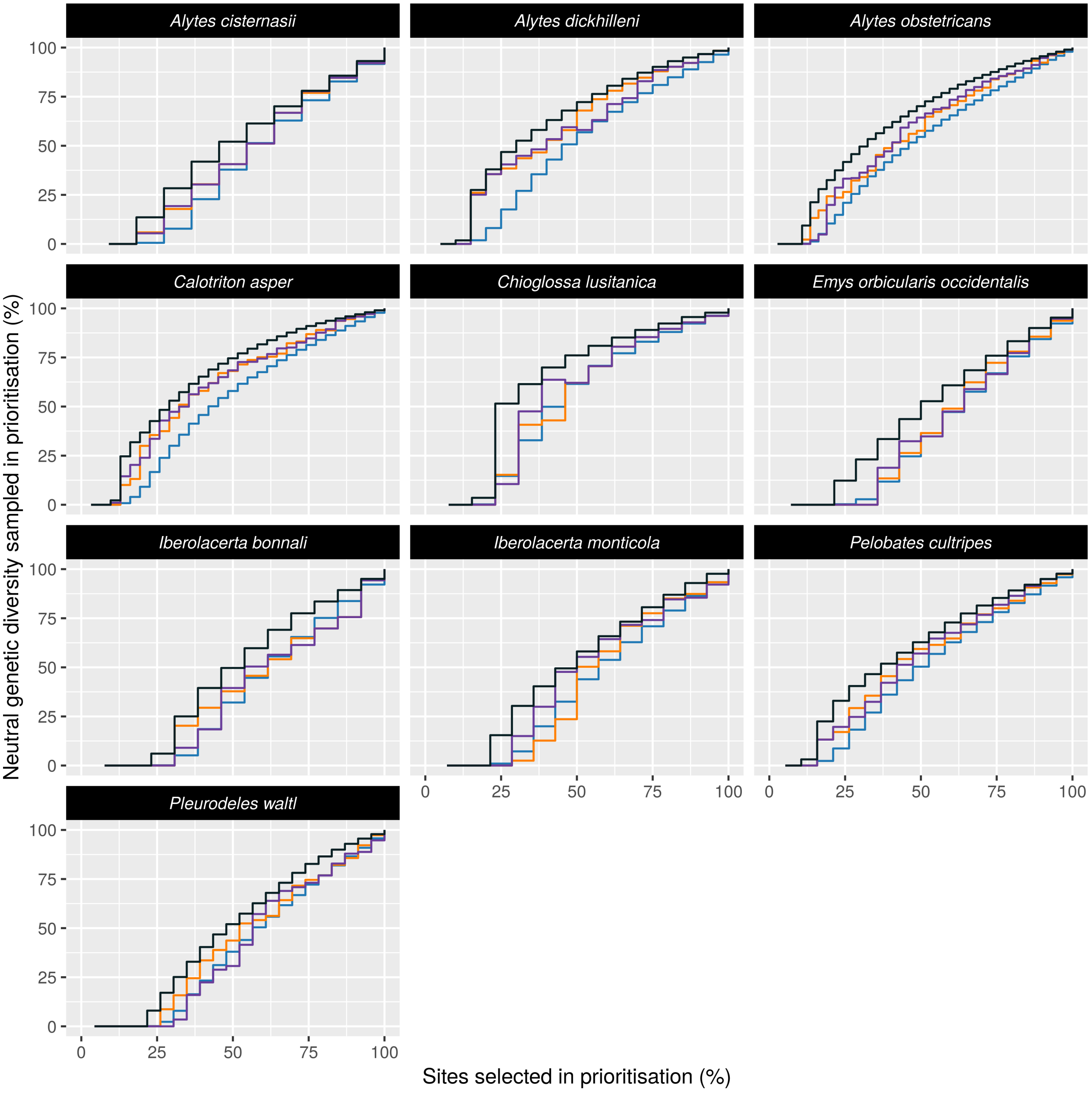

Appendix S90: The relationship between the percentage broad-scale neutral genetic diversity held in a prioritisation and the approach used to generate it. Broad-scale neutral genetic diversity was calculated using genetic distances (Jost's D) among sites and approaches 100% as the sites selected in a prioritisation secure a more representative sample of the diversity of allelic combinations measured across all sites. Lines show the prioritisations generated using (black) genetic distances, (orange) geographic distances, (purple) resistance distances, and (blue) the average performance of randomly selecting sites. As a consequence, there is the greatest support for the (orange and purple) surrogate-based prioritisations performing well when they approximate the (black) genetic-based prioritisations and are different from the (blue) randomly generated prioritisations.

Appendix S91: Summary of the generalized linear mixed effects model used to describe the performance of different prioritization approaches for representing broad-scale genetic diversity. Specifically, this table contains the fixed effects. As described in the main text, the response variable for this model was the proportion of broad-scale genetic diversity that a given prioritization secured. It was fit using continuous predictor variable describing the cost of each prioritization ("cost" term), a categorical predictor variable describing the method used to generate each prioritization ("method" terms, with the following levels: "random", "geographic distance", and "resistance distance"), and a predictor variable describing the interaction between these two variables ("cost:method" terms). The model intercept corresponds to prioritizations generated using genetic diversity directly. When interpreting these coefficients, note that the model was fit using a logit link.

Term	Estimate	Standard error
intercept	-2.705	0.455
cost	6.540	0.897
method (method = geographic distance)	-0.549	0.643
method (method = random)	-1.126	0.686
method (method = resistance distance)	-0.559	0.644
cost:method (method = geographic distance)	0.156	1.256
cost:method (method = random)	0.655	1.294
cost:method (method = resistance distance)	0.181	1.258

Appendix S92: Performance of prioritisations generated for broad-scale genetic diversity. Data show mean and standard deviation in performance values for each species. Specifically, performance values are expressed as the proportion of sites with high genetic diversity secured in a given prioritisation relative to those in optimal prioritisations based on genetic data with the same number of selected sites.

Species	Prioritisation approach	Performance
Alytes cisternasii	genetic distance	100% ± 0
	geographic distance	84.69% ± 0.19
	random	74.61% ± 0.34
	resistance distance	84.91% ± 0.19
Alytes dickhilleni	genetic distance	100% ± 0
	geographic distance	88.79% ± 0.22
	random	70.4% ± 0.33
	resistance distance	86.92% ± 0.22
Alytes obstetricans	genetic distance	100% ± 0
	geographic distance	85.02% ± 0.16
	random	75.01% ± 0.28
	resistance distance	83.75% ± 0.25
Calotriton asper	genetic distance	100% ± 0
	geographic distance	86.35% ± 0.21
	random	72.71% ± 0.3
	resistance distance	88.92% ± 0.13
Chioglossa lusitanica	genetic distance	98.41% ± 0.06
	geographic distance	77.62% ± 0.31
	random	76.91% ± 0.34
	resistance distance	80.05% ± 0.32
Emys orbicularis occidentalis	genetic distance	100% ± 0
	geographic distance	73.1% ± 0.36
	random	71.87% ± 0.34
	resistance distance	74.3% ± 0.34
Iberolacerta bonnali	genetic distance	100% ± 0
	geographic distance	79.79% ± 0.26
	random	73.43% ± 0.33
	resistance distance	74.98% ± 0.3
Iberolacerta monticola	genetic distance	100% ± 0
	geographic distance	74.49% ± 0.36
	random	75.4% ± 0.31
	resistance distance	84.73% ± 0.28
Pelobates cultripes	genetic distance	100% ± 0
	geographic distance	83.52% ± 0.24
	random	73.04% ± 0.33
	resistance distance	83.04% ± 0.25
Pleurodeles waltl	genetic distance	100% ± 0
	geographic distance	84.22% ± 0.22
	random	76.35% ± 0.29
	resistance distance	75.42% ± 0.32

Appendix S93: Maximum likelihood population effects (MLPE) model results for assessing the relationship between pairwise genetic distances (Jost's D) and surrogate distances between sites. Specifically, the two surrogate distances examined were geographic distances and resistance distances. Significance tests evaluate model performance relative to null models and had a single degree of freedom.

Species	Geographic distance			Resistance distance		
	χ^2	P	R^2	χ^2	P	R^2
Alytes cisternasii	18.4	**<0.001**	0.16 (0.03 – 0.36)	16.28	**<0.001**	0.07 (0 – 0.25)
Alytes dickhilleni	196.3	**<0.001**	0.67 (0.61 – 0.73)	169.35	**<0.001**	0.33 (0.23 – 0.44)
Alytes obstetricans	394.4	**<0.001**	0.44 (0.39 – 0.49)	387.03	**<0.001**	0.31 (0.26 – 0.37)
Calotriton asper	350.9	**<0.001**	0.43 (0.37 – 0.49)	430.54	**<0.001**	0.53 (0.47 – 0.58)
Chioglossa lusitanica	15.8	**<0.001**	0.11 (0.02 – 0.27)	9.24	**0.002**	0.06 (0 – 0.2)
Emys orbicularis occidentalis	19.8	**<0.001**	0.24 (0.11 – 0.39)	15.04	**<0.001**	0.14 (0.03 – 0.28)
Iberolacerta bonnali	9.9	**0.002**	0.09 (0.01 – 0.24)	11.34	**<0.001**	0.11 (0.01 – 0.26)
Iberolacerta monticola	43.2	**<0.001**	0.35 (0.21 – 0.49)	0.84	0.36	0.05 (0 – 0.4)
Pelobates cultripes	137.8	**<0.001**	0.5 (0.41 – 0.59)	173.36	**<0.001**	0.28 (0.18 – 0.39)
Pleurodeles waltl	86.4	**<0.001**	0.27 (0.19 – 0.37)	64.70	**<0.001**	0.1 (0.04 – 0.18)

Appendix S94: Performance of prioritisations for representing broad-scale genetic diversity compared to randomly generated prioritisations. Data show mean and standard deviation in performance values for each species.

Species	Prioritisation approach	Performance
Alytes cisternasii	genetic distance	98.97% ± 23.39
	geographic distance	59.34% ± 16.53
	random	0% ± 0
	resistance distance	60.62% ± 16.69
Alytes dickhilleni	genetic distance	121.3% ± 27.09
	geographic distance	102.96% ± 24.65
	random	0% ± 0
	resistance distance	101.45% ± 24.8
Alytes obstetricans	genetic distance	99.2% ± 22.86
	geographic distance	55.73% ± 14.88
	random	0% ± 0
	resistance distance	38.19% ± 9.77
Calotriton asper	genetic distance	94.64% ± 14.01
	geographic distance	61.38% ± 10.07
	random	0% ± 0
	resistance distance	63.92% ± 10.19
Chioglossa lusitanica	genetic distance	54.34% ± 5.73
	geographic distance	15.13% ± 2.55
	random	0% ± 0
	resistance distance	19.6% ± 3.3
Emys orbicularis occidentalis	genetic distance	82.38% ± 14.73
	geographic distance	12.38% ± 4.01
	random	0% ± 0
	resistance distance	20.11% ± 5.58
Iberolacerta bonnali	genetic distance	76.96% ± 7.95
	geographic distance	47.53% ± 6.29
	random	0% ± 0
	resistance distance	19.6% ± 3.1
Iberolacerta monticola	genetic distance	124.75% ± 32.37
	geographic distance	4.84% ± 4.36
	random	0% ± 0
	resistance distance	43.84% ± 10.77
Pelobates cultripes	genetic distance	91.18% ± 21.41
	geographic distance	52.33% ± 12.76
	random	0% ± 0
	resistance distance	50.57% ± 12.74
Pleurodeles waltl	genetic distance	64.09% ± 6.05
	geographic distance	31.46% ± 3.57
	random	0% ± 0
	resistance distance	1.61% ± 1.03
